# A Comprehensive Analysis of Small-Passerine Fatalities from Collision with Turbines at Wind Energy Facilities

**DOI:** 10.1371/journal.pone.0107491

**Published:** 2014-09-15

**Authors:** Wallace P. Erickson, Melissa M. Wolfe, Kimberly J. Bay, Douglas H. Johnson, Joelle L. Gehring

**Affiliations:** 1 Western EcoSystems Technology, Inc., Cheyenne, WY, United States of America; 2 U. S. Geological Survey, Northern Prairie Wildlife Research Center, Saint Paul, MN, United States of America; 3 Federal Communications Commission, Washington, DC, United States of America; University of Regina, Canada

## Abstract

Small passerines, sometimes referred to as perching birds or songbirds, are the most abundant bird group in the United States (US) and Canada, and the most common among bird fatalities caused by collision with turbines at wind energy facilities. We used data compiled from 116 studies conducted in the US and Canada to estimate the annual rate of small-bird fatalities. It was necessary for us to calculate estimates of small-bird fatality rates from reported all-bird rates for 30% of studies. The remaining 70% of studies provided data on small-bird fatalities. We then adjusted estimates to account for detection bias and loss of carcasses from scavenging. These studies represented about 15% of current operating capacity (megawatts [MW]) for all wind energy facilities in the US and Canada and provided information on 4,975 bird fatalities, of which we estimated 62.5% were small passerines comprising 156 species. For all wind energy facilities currently in operation, we estimated that about 134,000 to 230,000 small-passerine fatalities from collision with wind turbines occur annually, or 2.10 to 3.35 small birds/MW of installed capacity. When adjusted for species composition, this indicates that about 368,000 fatalities for all bird species are caused annually by collisions with wind turbines. Other human-related sources of bird deaths, (e.g., communication towers, buildings [including windows]), and domestic cats) have been estimated to kill millions to billions of birds each year. Compared to continent-wide population estimates, the cumulative mortality rate per year by species was highest for black-throated blue warbler and tree swallow; 0.043% of the entire population of each species was estimated to annually suffer mortality from collisions with turbines. For the eighteen species with the next highest values, this estimate ranged from 0.008% to 0.038%, much lower than rates attributed to collisions with communication towers (1.2% to 9.0% for top twenty species).

## Introduction

Wind energy production in the United States (US) and Canada has increased greatly in recent years. More so than for any other industry, monitoring the effects of wind turbines on wildlife has been an integral part of this development. For example, the US Fish and Wildlife Service (USFWS) provided guidelines to wind energy developers and identified the following species of concern that could be affected by development: “migratory birds; bats; bald and golden eagles and other birds of prey; prairie and sage grouse; and listed, proposed, or candidate endangered and threatened species” [1]. Research is on-going regarding the effect of wind turbines on bats, raptors, and grouse (e.g., [2]–[8]). In addition, several efforts have been made to broadly quantify the effects on birds [9]–[13], and statistical methods associated with these efforts have evolved.

In this paper we use new methods to quantify effects for birds known as passerines (sometimes referred to as songbirds or perching birds). Many passerine species are migratory and protected by the Migratory Bird Treaty Act (MBTA) [1]. Passerines are the most common type of bird observed both during surveys prior to construction and as fatalities resulting from collisions with turbines after construction [14]. The population-level effect for most small-passerine species may be smaller compared to other bird types, in part because they are shorter-lived and typically reproduce at a higher rate than other taxa, such as raptors [15], [16]. However, we are not aware of any existing comprehensive analyses specifically addressing the interactions of passerine species with wind turbines. This analysis will provide federal and state regulatory agency personnel, the wind industry, and other stakeholders with a better understanding of the overall rate of passerine fatalities from collisions with wind turbines and identify research and monitoring needs.

Our objectives for this evaluation were to 1) identify monitoring studies from wind energy facilities in the US and Canada that contained adequate information for evaluation of small-bird fatality rates; 2) derive estimates for rates of annual mortality for small birds in studies that did not include small-bird mortality rates but rather reported mortality rates for all birds combined; 3) adjust all small-bird rates for bias and derive biome-level and continent-wide rates; 4) determine the seasonal timing of fatalities for small passerines; and 5) estimate the population-level effect for species of small passerines in the US and Canada.

## Methods

“Fatality” refers to birds colliding with turbines unless otherwise indicated. While the term fatality is used throughout the paper, cause of death of carcass finds is typically not identified easily, especially for small birds. We defined small passerines as bird species belonging to the taxonomic order Passeriformes [17] but excluded birds that are 30.5 cm or greater in length according to the Sibley Guide to Birds [18]. This was done to decrease variability associated with bird size. Excluded types are larger species in the Corvidae family (crows, etc.). Because some monitoring data did not distinguish species of passerines from other species of small birds, we use the term “small bird” to define all bird species that belong in the same above-defined size class. We estimated rates of fatality for small birds, and although some fatalities were small but not passerines, most of the fatalities were passerines.

### Studies of Bird Fatality at Wind Energy Facilities

We used studies from the United States and Canada that were conducted after wind energy facilities were constructed; they report results of surveys conducted to monitor the number of birds killed by striking turbines and the resulting estimated annual fatalities rates. Some studies were conducted at wind energy facilities that were built in phases rather than being built all at once. Most studies also included results from experimental trials (hereafter bias trials) that assessed searcher efficiency and the influence of scavenger activities on detection of carcasses. Bias trials measured how effective observers were at finding dead birds on the ground and how long it would take for scavengers to remove the bird carcasses, which could occur before an observer had the opportunity to find it.

**Figure 1:**
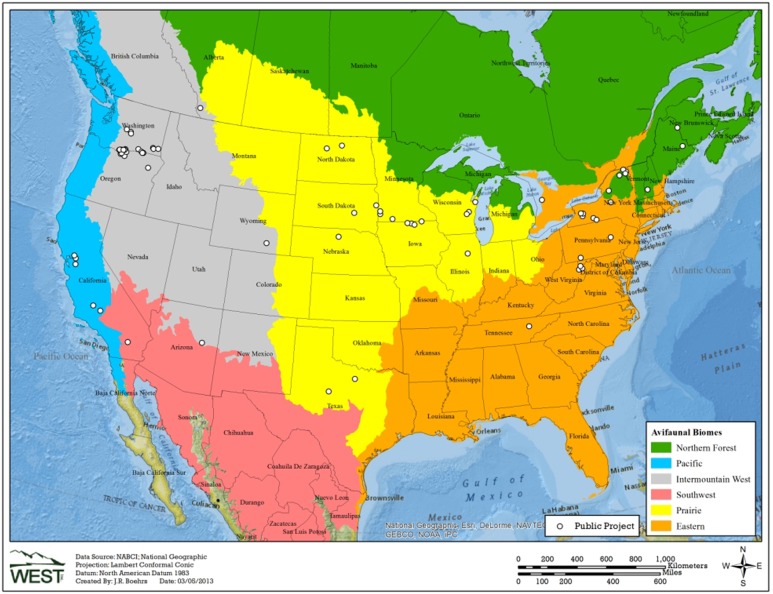
Locations of studies included for analysis of small-bird fatalities at wind energy facilities. The locations of wind energy facilities associated with 116 studies that were appropriate for inclusion in an analysis of fatalities of small passerines due to collisions with wind turbines. See details of studies in Appendix S1. Biomes adapted from Rich et al. [19] [58] [59] [60].

Data from 116 studies at more than 70 wind energy facilities were appropriate for analysis (see “Selection Process, Assumptions and Potential Biases” section below for details on how studies were chosen for inclusion). We identified, described, and mapped each study and its location with ArcGIS software; we further categorized each study according to regions called avifaunal biomes which are broad areas of ecologically similar lands (Figure 1) [19]. Biomes are made up of bird conservation areas (BCRs), defined as “ecologically distinct regions in North America with similar bird communities, habitats, and resource management issues.” [20]. Two related studies on bird fatalities due to communication towers in the eastern US used groupings of BCRs as an organizing unit [21], [22]. We organized our data by biomes to reach a better sample size, as the study locations were distributed across the United States and southern Canada and our focus was continent-wide. We gathered facility-specific information including number of turbines, turbine model, turbine tower height and blade length, nameplate megawatt (MW) capacity, and vegetation cover (Appendix S1). We obtained information about study methodology including duration, the interval between searches for carcasses, plot size, number and type of carcasses used in bias trials, and the type of estimator used to correct for bias. Study results included species composition and counts of fatalities, searcher efficiency, carcass removal rates, and other information regarding carcasses (e.g., date found, state of carcass, nearest turbine, evidence of scavenging, etc.). Although more than one study was included for some locations, each study represents independent searches and trials. For our calculations we used the rate of bird fatalities/MW/year provided in each report based on bird fatalities found during standardized searches. For our analysis of timing and taxonomic composition of bird fatalities we included bird fatalities found within the standardized search plots both during standardized searches and incidentally.

### Estimator Types

Scientists use mathematical equations referred to as estimators to adjust rates of fatality for detection and scavenging biases because not all birds killed by colliding with wind turbines can be detected during surveys. Most studies of turbine-related fatalities include standardized carcass searches that are conducted at regular intervals along transects at a subset of turbines. Searcher efficiency measures the proportion of carcasses present that are found by observers; searcher efficiency is usually is less than 1, because observers are typically unable to discover all carcasses. Carcass removal rates quanitify the rate at which carcasses are not detectable because of scavenging (e.g., an animal picks up the dead bird and carries it off), or cannot be detected because of weathering, decomposition, or other means (e.g., plowing in a field). Thus, the basic formula for estimators of fatality rates is equal to the observed mean number of carcasses found per turbine, divided by the estimated average probability that a carcass is both available to be found during a search (i.e., one minus the carcass removal rate) and is in fact found (i.e., searcher efficiency).

To date, the four most prevalent estimators used to calculate fatality-rate estimates are the Shoenfeld [23], Jain [24], Huso [25], and Naïve estimators [25], [26]. Seventy-four of our chosen studies reported estimates calculated using the Shoenfeld estimator, 22 studies used the Jain estimator, 10 used the Naïve estimator, nine used the Huso estimator, and one used the unique estimator developed by Environment Canada (Appendix S2). The Naïve estimator in Huso [25] was originally used in studies with long search intervals where bias was relatively small, but was later inappropriately applied to studies using methods that violated the assumptions of the method [14]. The Naïve estimator typically is not used in more-recent monitoring studies. The Jain estimator accounts for removal and searcher efficiency bias by dividing the observed-fatality rate by the product of the proportion of trial carcasses not removed after half the search interval, and the proportion of carcasses found by searchers. In contrast, both Huso and Shoenfeld estimators calculate the probability of availability and detection by use of equations involving the average removal time in days, based on an exponential distribution of carcass decay, the searcher efficiency proportion, and the average search interval.

The Shoenfeld and Huso estimators generally produce similar results when search intervals are long and carcass persistence times are short [25]. However, Shoenfeld and Huso estimates may be quite different when search intervals are short and carcass persistent times are long. In general, the Shoenfeld estimator tends to be biased low with respect to the true fatality rate and the Huso estimator tends to be biased high [9]. Due to the exponential component in each formula, both estimators are sensitive to changes in average removal time. The Huso estimator may also overestimate fatality rates unless methods for obtaining searcher-efficiency rates allow for multiple opportunities to find a carcass. However, most studies conducted to date have used a single search for searcher efficiency, which can inflate estimates calculated using Huso’s method. Because the Shoenfeld estimator assumes equal probability of detection for each search, multiple searches for searcher efficiency trials are not needed.

In a simulation study that compared accuracy of the Jain, Huso, and Shoenfeld estimators, results indicated the Jain estimator slightly underestimated rates of fatality, whereas the Huso estimator tended to overestimate rates but to a lesser extent when carcass removal was rapid and/or the efficiency of searchers declined substantially over time [27]. The Shoenfeld method underestimated fatality rates, especially with rapidly declining searcher efficiency over time, which is logical because constant searcher efficiency over time is one of its assumptions. For this analysis, we assumed that the Naïve estimator is about 20% worse than the Shoenfeld estimator, i.e., it underestimates or overestimates fatality rates even more than Shoenfeld.

### Selection Process, Assumptions and Potential Biases

We assumed that the results of studies available to us were representative of unsampled or unreported sites and the studies included in our analysis were standardized to the extent possible. We did not include some studies because they 1) lacked the capacity to be standardized, 2) used older methods, 3) used inappropriately long search intervals, or 4) were conducted at older-generation wind energy facilities that are not representative of current facilities. The data we used were selected from reports representing over 100,000 surveys at turbines. While only two Canadian facilities are included here, Bird Studies Canada maintains a Wind Energy Bird and Bat Monitoring database of fatality studies, of which summary data are available. We excluded reports for older generation turbines because they are not representative of current turbine design and the search interval for these studies tended to be longer (e.g., 30 days), decreasing the likelihood that a small bird would be detectable. Older generation turbines are included in the total MW of current generating capacity used to calculate our estimate of small-bird fatality rates for all biomes and the US and Canada combined, making these estimates more conservative. In addition, many of the turbines in the older facilities are being repowered with newer generation turbines.

Several biases might exist in our analyses. We assumed that all dead birds were observed on standardized search plots and deaths were caused by collision with turbines and not caused by other sources (i.e., background mortality). Additionally, scavenging levels may vary within season and from one season to the next, making it more difficult to measure true scavenging effects. Species typically used in searcher efficiency and carcass removal trials (e.g., house sparrows [*Passer domesticus*] and *Coturnix* species of quail) may not be representative of fatalities found (e.g., they differ in size or coloration). Estimation of species-specific fatality rates with the scaling method explained below assumes equal detection rates among the species of small passerines, which is unlikely. For example, a brightly colored male warbler likely has higher detectability than a drab-colored sparrow. This assumption may cause some bias, but it is likely not large enough to substantially detract from the value of these estimates. Finally, some bird fatalities fall outside of search plots.

### Deriving Small-Bird Fatality Rates from All-Bird Fatality Rates

We used the estimated fatality rate for small birds (number of fatalities/MW/year) reported by each study to calculate estimates of fatality rates for the US and Canada and for each avifaunal biome. Eighty-one studies specifically reported estimates for small-bird fatality rates, which ranged from zero to 7.67 birds/MW/year (Appendix S2). The remaining 35 studies reported estimated rates for all bird fatalities combined, regardless of size. To make all studies comparable, we derived a small-bird-only estimate from the reported estimate for all birds (see below).

Deriving estimates of small-bird fatality rates required data on the number of dead birds observed, the estimated searcher efficiency rate, the estimated carcass removal rate, and the average search interval. In addition to tallies of large- and small-bird fatalities, differences in searcher efficiency and carcass removal times between large and small birds need to be accounted for when attempting to determine the estimate of the small-bird fatality rate from an all-bird estimate. We derived the estimate of the small-bird fatality rate (*m*_SB_) from the all-bird fatality rate estimate (*m*_all birds_) with the following calculation, where *m* is the estimated average number of fatalities per turbine per year, adjusted for removal and searcher efficiency bias; %compLB and %compSB are the proportions of fatalities that are large birds and small birds, respectively; and 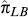 and 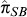 are the estimated probabilities that a large carcass and small carcass are both available to be found during a search and actually are found, as estimated from the removal trials and the searcher efficiency trials.
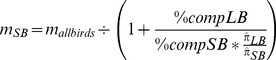
When some of the necessary information was not available, we used a general conversion method. When this was the case, available data for each biome were used to calculate an average regional (biome-specific) carcass removal time (range is 4.51–24.52 days) and searcher efficiency rate for small and large birds (range is 0.48–0.89), and average search interval (range is 1–28 days), and probability of detection (range is 0.08–0.97; Appendix S3). Prior to analysis, we tested the quality of the conversion equation by calculating estimated small-bird rates for studies containing both all-bird and small-bird rates. We compared the calculated value with the small-bird rate reported in the study, and the resulting R^2^ was 0.93 (Figure S1).

#### Searcher Efficiency Values

Searcher efficiency rates for small birds were available for 98 studies, and ranged from 16.6% to 86.6% (the percent of trial carcasses that are detected by searchers in the searcher efficiency trials; Appendix S4). Some studies using the Huso estimator calculated searcher efficiency based on placing and leaving a carcass in the survey area for several days, providing multiple opportunities for the carcass to be detected by an observer. When possible, searcher efficiency values estimated in this way were adjusted to reflect single-search values to make comparisons to other studies that provided values from a single search. When multiple years of study were conducted, all data were combined into a single searcher efficiency value estimate for that project. For projects that did not report searcher efficiency rates, we attempted to determine searcher efficiency from data in the report. All searcher efficiency values in each biome were combined to obtain regional searcher efficiency rates. Regional rates were used instead of a single continental rate to account for regional differences in searcher efficiency (and carcass removal, below) due to differences in topography and vegetation characteristics.

#### Carcass Removal Values

We estimated the average number of days for the removal of small-bird carcasses in 70 studies; this ranged from 1.64 to 27.8 days (Appendix S4). For projects that did not report mean removal rates, we attempted to determine the average duration of carcasses remaining from the data in the report. Regional carcass removal rates were calculated using all values for each avifaunal biome.

#### Probability of Detection

With the bias-trial values for each avifaunal biome, the probabilities of availability and detection were calculated for search intervals of 1, 3, 7, 14, and 28 days. The proportions of large- and small-bird fatalities in each biome were then combined with the probability of availability and detection to calculate a multiplier for each search interval. For each biome we averaged the results for the five search interval values to calculate a multiplier, which we used to convert all-bird estimates of fatality rates to small-bird estimates for all projects in that biome that did not include a small-bird rate estimate in their report (Appendix S5). Although this method probably overestimated small-bird fatality rates for projects with daily searches and underestimated fatality rates for projects with long search intervals (e.g., 14 days), it likely provides a better adjustment than the proportion of small birds alone as it accounted for differences in detectability and carcass removal rates between large and small birds.

### Bias Adjustments

After we calculated estimates of small-bird fatality rates for the 35 studies that only reported estimates for all birds, we adjusted small-bird rates of all studies for bias based on the type of estimator used [27]. Each study design was different (although studies conducted during different phases at the same wind energy facility tended to have similar methods), so we created a customized adjustment factor for each study based on 1) estimator method used, 2) search interval (e.g., weekly, bi-weekly, etc.), and 3) classification of both bias trial results (Appendix S6). If a project had a search interval different from any category then the two surrounding bias adjustment categories were used, e.g., if the project used 3-day search intervals, the 1-day and 7-day bias adjustments were investigated. It is not possible to determine the rate at which the efficiency of searchers may change over time at each project, e.g., between searcher efficiency trials. Therefore, searcher efficiency rates (proportion found) within each study were averaged and categorized as low (0–0.375), moderate (0.375–0.65), or high (0.65–1). We classified the overall average value for carcass removal as fast (0–10 days), moderate (11–23 days), or slow (24 or more days). For each combination of these four factors, we determined the lowest and highest bias adjustment values, based on trial simulations presented in Erickson et al. [27]. The bias adjustment value was relative to 1: if the value was equal to 1 no adjustment was made, indicating no bias in the estimator; for other values, the further they were from one, either higher or lower, the more they adjusted the original estimate. These two bias adjustments were multiplied by the estimated small-bird fatality rate for each project, resulting in two fatality rates, one using the lowest bias correction and one using the highest. For each biome the project rates were then averaged and resulting values were further multiplied by the current operating capacity to generate an estimate of the number of small bird fatalities using both bias correction values.

### Estimation of Species-Specific Numbers for Small Passerines

For each species in each biome its proportion of the total small passerine fatalities was multiplied by the total number of small-bird fatalities estimated using adjustments with the lowest and highest values. The results were summed across biomes. To adjust for the absence of data from the southwest biome, all estimates for species were multiplied by seven percent, which is the proportion of continent-wide operating capacity that the southwest biome represents. Finally, the number of continent-wide fatalities estimated for each species was compared to the overall population size estimated for each species.

An *a posteriori* examination of the timing of fatalities (see Timing of Small-Passerine Fatalities Results below) and range maps of individual species [18] reflected that several species, especially warblers, consist of populations with a distinctive migration pattern. Thirty-three species have ranges that extend from eastern to western Canada and in autumn the more western individuals of several species travel east first and then south, in essence retracing the route of their hypothesized evolutionary breeding-range expansion [28]. Therefore many more individuals migrate through the eastern US than actually breed within biomes in this area or directly to the north in eastern Canada. Consequently, it was more logical to compare effects of turbine-related fatality on individual species for continent-wide populations than to those associated with individual biomes.

### Estimation of Bird Population Sizes

Estimates of population sizes for species of small passerines were obtained from the Partners in Flight (PIF) Land Bird Population Estimates Database [29], which is based on annual Breeding Bird Surveys (BBS) coordinated by the US Geological Survey and Canadian Wildlife Service. Breeding Bird Surveys are roadside counts designed to “estimate population trends and relative abundances at various geographic scales” [30], and the PIF database can be used to estimate population size for a specific species across the continent or at a biome level [31]. The PIF population estimates were based on data collected between 1998 and 2007, and raw data are adjusted for factors such as assumed average detection distances, pair occurrence, and time of day [31], [32].

Some population estimates have relatively large standard errors associated with the BBS count average rate, due to high variance in counts and/or small numbers of BBS routes surveyed [31]. In addition, several potential sources of bias exist in these estimates. Potential sources of bias include non-random sampling of landscapes. Also, species detected near habitats altered by roads may not represent the species composition of areas away from roads. For example, traffic may affect the presence of birds or detectability, and some naturally secretive species may be present but not detectable [31] [33], [34]. However, McCarthy et al. [35] evaluated species distribution models from unpaved roadside counts similar to BBS counts in a control-impact study, finding that roadside counts do provide adequate model accuracy compared to the off-road data if an adequate range of environmental gradients is sampled. Reasonable concerns regarding bias exist, however, a measure of population sizes for small passerines is required to discuss the effects of small-passerine fatalities and the PIF population database currently provides the best estimates of breeding bird population sizes in the US and Canada.

The following describes migration strategies relevant to this analysis: resident birds are present year-round in a particular location or region; breeding birds reproduce in a given location or region; wintering birds remain in a location or region for an extended period of time during the winter months and travel elsewhere to breed; migrant birds are birds in the process of traveling between breeding and wintering locations, typically at regular times, and often over long distances; and nocturnal migrants are birds that migrate at night, often over long distances. Most small passerines that breed in the US and Canada are nocturnal migrants that spend the winter in more southern latitudes [16].

## Results

Most of the 116 available monitoring studies that were appropriate for our analyses were conducted in the northern third of the contiguous United States (Figure 1). Agriculture was the only land use identified in 30 studies (Table 1). Ninety-seven monitoring studies occurred in either agricultural, grassland, or forested land cover, or some combination thereof. The remaining 19 studies reported land covers of desert, shrub-steppe, or rocky embankments (Table 1).

**Table 1:** The land cover type associated with studies of collisions of birds with wind turbines at wind energy facilities in geographically distinct avifaunal biomes, for studies that reported small-bird and all-bird only estimates of fatality rates.

Avifaunalbiome	Land cover type	#Projects withsmall-bird estimates	#Projects withall-bird estimates only
**Eastern**	Agriculture	1	3
	Agriculture/forest	3	3
	Forest	3	4
	Forest/pasture/grassland	0	1
	Grassland	0	2
**Intermountain** **West**	Agriculture	4	2
	Agriculture/grassland	13	1
	Desert grassland/forested	2	0
	Grassland	4	1
	Grassland &shrub steppe	3	1
	Grassland/shrub steppe & agriculture	6	1
	Grassland/shrub steppe, agriculture & forest	0	1
	Shrub steppe & agriculture	2	0
**Northern** **Forest**	Agriculture	1	0
	Agriculture/forest	6	0
	Forest	1	5
	Grassland, forest, rocky embankments	1	1
**Pacific**	Agriculture	1	0
	Agriculture/grassland	1	2
	Desert	1	0
	Grassland	1	0
	Shrub/scrub & grassland	1	0
	No habitat listed	1	0
**Prairie**	Agriculture	13	5
	Agriculture/forest	1	0
	Agriculture/grassland	6	2
	Forest	1	0
	Grassland	4	0
**Total**	**116 projects**	**81**	**35**

### Fatality Rate Estimates for Small Birds

We calculated estimates of fatality rates for small birds for 35 studies in which only estimates combining all birds were reported. The resulting calculated values ranged from 0.18 to 9.65 fatalities/MW/year (Table 2). These were derived using the small-bird multiplier values, which ranged from 0.67 to 0.88 depending on region (Appendix S5).

**Table 2:** Estimated fatality rate (birds/megawatt(MW)/year) and confidence interval calculated for small birds in studies of bird collisions at wind energy facilities that provided all-bird estimates only in their report, along with estimator, all-bird rate and confidence interval, and multiplier value^a^.

Project name byavifaunal biome	Estimatorused	All-bird fatalityrate estimate(MW/year)	All-birdConfidenceinterval	Multiplier	Calculated small-birdfatality estimate(MW/year)	Calculated small-birdConfidenceinterval
**Eastern Biome**						
Buffalo Mountain (2000–2003)	Naïve	11.02		0.88	9.65	
Buffalo Mountain (2005)	Naïve	1.10		0.88	0.98	
Casselman (2008)	Shoenfeld	1.51	0.90–4.00	0.88	1.33	0.53–2.34
Casselman (2009)	Shoenfeld	2.88	2.67–6.44	0.88	2.52	1.56–3.76
Cohocton/Dutch Hill (2009)	Jain	1.39		0.88	1.20	
Cohocton/Dutch Hills (2010)	Jain	1.32		0.88	0.69	
Locust Ridge II (2009)	Shoenfeld	0.84		0.88	0.74	
Locust Ridge II (2010)	Shoenfeld	0.76		0.88	0.66	
Mountaineer (2003)	Shoenfeld	2.69	2.41–8.33	0.88	2.36	1.41–4.87
Munnsville (2008)	Jain	1.48		0.88	1.30	
Ripley (2008)	Environment Canada	3.09		0.88	2.70	
Sheldon (2010)	Shoenfeld	1.76	1.66–3.88	0.88	1.54	0.97–2.27
Sheldon (2011)	Shoenfeld	1.57	1.46–3.36	0.88	1.38	0.85–1.96
**Intermountain West Biome**						
Big Horn	Huso	2.54	2.59–7.54	0.78	1.97	1.34–3.90
Harvest Wind (2010–2012)	Huso	2.94	4.93–10.00	0.78	2.28	1.66–3.37
Leaning Juniper	Huso	6.66	6.19–15.66	0.78	5.17	3.20–8.10
Pebble Springs	Huso	1.93	2.34–8.89	0.78	1.50	0.86–3.29
Summerview (2006)	Environment Canada	1.06		0.78	0.82	
Tuolumne (Windy Point I)	Shoenfeld	3.20	4.89–11.57	0.78	2.49	1.72–4.08
White Creek (2007–2011)	Huso	4.05	7.64–12.12	0.78	3.14	2.58–4.09
**Northern Forest Biome**						
Lempster (2009)	Shoenfeld	3.38	3.75–9.78	0.81	2.73	1.52–3.96
Mars Hill (2007)	Jain	1.67		0.81	1.33	
Mars Hill (2008)	Jain	1.76		0.81	1.43	
Stetson Mountain I (2009)	Jain	2.68		0.81	2.17	
Stetson Mountain I (2011)	Jain	1.18	1.54–1.99	0.81	0.96	0.83–1.07
Stetson Mountain II (2010)	Jain	1.42	1.91–2.37	0.81	1.15	1.03–1.28
**Pacific Biome**						
High Winds (2004)	Shoenfeld	1.62		0.67	1.08	
High Winds (2005)	Shoenfeld	1.10		0.67	0.73	
**Prairie Biome**						
Barton I and II	Shoenfeld	5.50	8.00–16.09	0.68	3.73	2.71–5.46
Kewaunee County	Shoenfeld	1.95		0.68	1.33	
Moraine II	Shoenfeld	5.59	3.58–15.22	0.68	3.79	1.62–6.88
Pioneer Prairie I (phase II)	Shoenfeld	0.27	0–1.35	0.68	0.18	0–0.55
Prairie WindsND1/Minot (2010)	Shoenfeld	1.48	1.74–3.33	0.68	1.04	0.82–1.56
Top of Iowa 2003	Shoenfeld	0.42		0.68	0.29	
Top of Iowa 2004	Shoenfeld	0.81		0.68	0.57	

a References for the studies listed in this table may be found in Appendix S1.

### Bias-Corrected Estimates of Fatality Rates for Small Birds

After we determined values for all studies, we adjusted estimates of fatality rates for small birds for bias based on the type of estimator used. Values used for bias adjustment ranged from 0.39 to 2.77, resulting in revised rates from 0 to 18.54 small-bird fatalities/MW/year (Appendix S7).

### Composition of Fatalities by Bird Type, Passerine Family, and Small-passerine Species

Small passerines accounted for 62.5% of the 4,975 observed fatalities at wind energy facilities; this included birds found incidentally, outside of standardized surveys (Table 3). Upland game birds (8.2%) and diurnal raptors (7.8%) were the next most commonly found bird types. Each of the other identifiable bird types accounted for less than 4% of all bird fatalities (Table 3). Some avifaunal biomes are represented by more studies than others (Figure 1), so the fatality composition for all biomes combined may not reflect that of individual biomes.

**Table 3:** Observed number of fatalities (including incidental finds) and percent composition of total for each bird type and passerine sub-type (family) for 116 studies at wind energy facilities in the United States and Canada.

Bird type	#Fatalities	% Composition
Passerines	3,110	62.5
Larks (Alaudidae)	681	13.7
Wood-warblers (Parulidae)	536	10.8
Vireos (Vireonidae)	322	6.5
Blackbirds/Orioles (Icteridae)	302	6.1
Sparrows (Emberizidae)	299	6.0
Kinglets (Regulidae)	221	4.4
Unidentified Passerines	126	2.5
Thrushes (Turdidae)	122	2.5
Starlings (Sturnidae)	103	2.1
Flycatchers (Tyrannidae)	79	1.6
Swallows (Hirundinidae)	69	1.4
Wrens (Troglodytidae)	61	1.2
Tanagers/Grosbeaks/Cardinals (Thraupidae/Cardinalidae)	32	0.6
Finches/Crossbills (Fringillidae)	30	0.6
Small Corvids (Corvidae)	25	0.5
Mimids (Mimidae)	23	0.5
Nuthatches (Sittidae)	23	0.5
Old World Sparrows (Passeridae)	15	0.3
Waxwings (Bombycillidae)	15	0.3
Creepers (Certhiidae)	10	0.2
Shrikes (Laniidae)	6	0.1
Longspurs/Buntings (Calcariidae)	5	0.1
Titmice/Chickadees (Paridae)	4	0.1
Gnatcatchers (Polioptilidae)	1	<0.1
Upland Game Birds	407	8.2
Diurnal Raptors	386	7.8
Unidentified Birds	260	5.2
Doves/Pigeons	192	3.9
Waterfowl	133	2.7
Vultures	71	1.4
Owls	62	1.2
Rails/Coots	54	1.1
Woodpeckers	52	1.0
Shorebirds	49	1.0
Large Cuckoos	45	0.9
Large Corvids	38	0.8
Swifts/Hummingbirds	37	0.7
Goatsuckers	25	0.5
Gulls/Terns	24	0.5
Loons/Grebes	18	0.4
Waterbirds	9	0.2
Kingfishers	3	0.1
**Overall**	**4,975**	**100**

At the level of passerine families, six of 24 taxa made up about half (47.5%) of all bird fatalities from wind energy developments in our analysis (Table 3): Alaudidae (larks; 13.7% of all fatalities), Parulidae (wood-warblers; 10.8%), Vireonidae (vireos; 6.5%), Icteridae (blackbirds/orioles; 6.1%), Emberizidae (sparrows; 6.0%) and Regulidae (kinglets; 4.4%). These taxa combined also made up over three-quarters of all small-passerine fatalities. All other families of passerines each made up 2.5% or less of the total number of turbine-related fatalities.

The studies documented fatalities of 246 identifiable avian species, of which 156 were unique species of small passerines (Appendices S8, S9). The most common species of small passerine found as a fatality was horned lark (*Eremophila alpestris*; 21.9% of small passerines), followed by red-eyed vireo (*Vireo olivaceus*; 8.5%), western meadowlark (*Sturnella neglecta*; 5.1%), and golden-crowned kinglet (*Regulus satrapa*; 5.1%; Table 4).

**Table 4:** Number of fatalities caused by collision with wind turbines and percent of all small-passerine fatalities (n = 3,110) for the 25 most commonly found species of small passerines in 116 studies conducted at 71 wind energy facilities from 1996–2012.

Common name^a^	Species	#Fatalities^a^	% Small-passerine fatalities
horned lark	*Eremophila alpestris*	681	21.9
red-eyed vireo	*Vireo olivaceus*	265	8.5
western meadowlark	*Sturnella neglecta*	159	5.1
golden-crowned kinglet	*Regulus satrapa*	158	5.1
unidentified passerine		120	3.9
European starling	*Sturnus vulgaris*	103	3.3
red-winged blackbird	*Agelaius phoeniceus*	70	2.3
magnolia warbler	*Setophaga magnolia*	60	1.9
yellow-rumped warbler	*Setophaga coronata*	57	1.8
ruby-crowned kinglet	*Regulus calendula*	55	1.8
dark-eyed junco	*Junco hyemalis*	52	1.7
blackpoll warbler	*Setophaga striata*	50	1.6
Townsend’s warbler	*Setophaga townsendi*	38	1.2
savannah sparrow	*Passerculus sandwichensis*	37	1.2
white-crowned sparrow	*Zonotrichia leucophrys*	37	1.2
tree swallow	*Tachycineta bicolor*	34	1.1
unidentified warbler		34	1.1
American robin	*Turdus migratorius*	28	0.9
black-throated blue warbler	*Setophaga caerulescens*	27	0.9
Wilson's warbler	*Cardellina pusilla*	27	0.9
common yellowthroat	*Geothlypis trichas*	26	0.8
unidentified sparrow		25	0.8
wood thrush	*Hylocichla mustelina*	25	0.8
Brewer’s blackbird	*Euphagus cyanocephalus*	24	0.8
bobolink	*Dolichonyx oryzivorus*	22	0.7
ovenbird	*Seiurus aurocapilla*	22	0.7
house wren	*Troglodytes aedon*	20	0.6
red-breasted nuthatch	*Sitta canadensis*	20	0.64

a Unidentified small-passerine types are also included in order of abundance. A full list of species is provided in Appendix S9.

### Timing of Small-Passerine Fatalities

Seventy-nine fatality studies provided the date for each bird fatality identified; all of these studies reported data collection in spring, summer, and fall for at least one year. For some studies data were not collected for a short time in the summer or winter, and we considered this acceptable for inclusion here. A peak in fatalities of small passerines occurred in fall, and a smaller peak occurred in spring (Figure 2). The fewest fatalities were found in December and January. Timing of fatalities for small-passerine families and species of interest to this analysis generally follow this same temporal pattern, but to a lesser extent when fewer individual fatalities comprised the group (Figures S2–S27).

**Figure 2:**
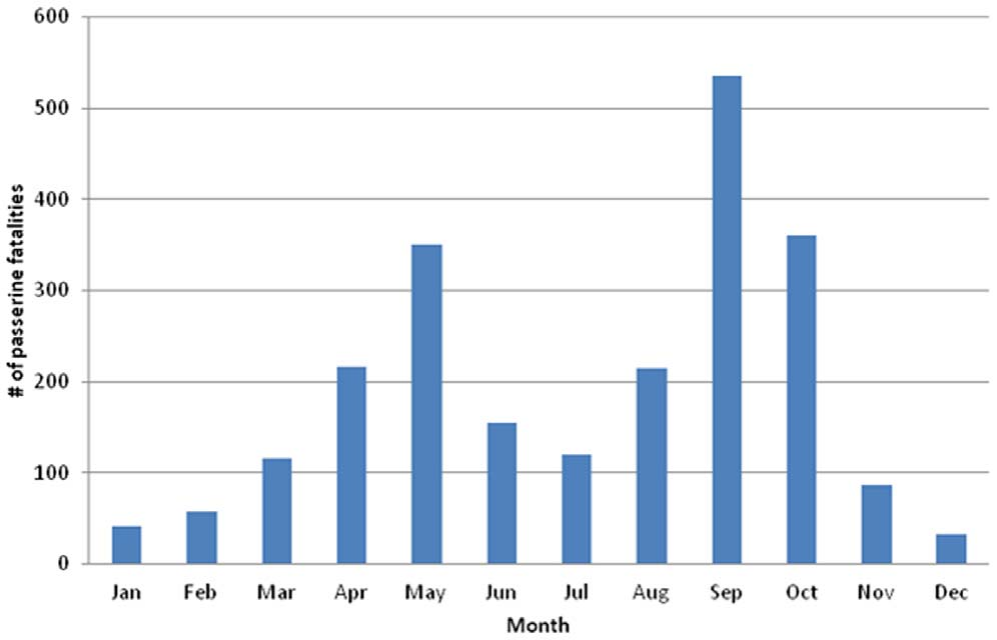
Monthly timing of small-passerine fatalities caused by collision with turbines and documented in 79 studies. The date for collision was provided in 79 studies on mortality of birds at wind energy facilities. A total of 2,285 fatalities for species of small passerines (less than 30.5 cm in size) were included and are sorted by month.

### Continent-wide and Biome-level Fatality Rates based on Operating Capacity

Across all biomes, the yearly fatality rate estimated for small birds was about 3.35/MW installed capacity when adjusted by the bias value that was the lowest (Table 5). The rate was 2.10 when adjusted by the bias value that was the highest. The studies in our analysis represent about 15% of the 63,023 MW of installed wind energy across all avifaunal biomes that have both wind energy development and data herein (as of January 2013) [36], [37]. At the biome level, studies in this analysis represented from about 18%–40% of the current operating capacity for all biomes except for the prairie and southwest biomes (7% and zero percent, respectively; Table 5). The average fatality rate calculated for small birds was lowest for the northern forest biome (1.43 birds/MW/year) and highest for the prairie biome (3.96 birds/MW/year), based on the most conservative bias adjustment. The most conservative rate for the eastern biome (3.83) was similar to that of the prairie biome.

**Table 5:** Comparison of the average fatality rate (birds/megawatt [MW]/year) for small birds for each associated avifaunal biome and all biomes combined, total MW produced in each biome, proportion of total MW represented by wind energy facilities with available fatality monitoring reports, and estimated number of small-bird fatalities annually.

Avifaunalbiome	Unadjusted averagesmall-bird estimate(MW/Year)	Averageestimate(MW/year)adjusted bylowest biasvalue	Average estimate(MW/year) adjusted byhighest bias value	Sum ofMW foravailabledata	Total MWinbiome^a^	Percent oftotal MWrepresented byavailable data	Number ofestimated annualfatalitiesadjusted bylowest biasvalue^b^	Number ofestimated annualfatalities adjustedby highestbias value^b^
Eastern	2.34	3.83	2.58	1,139.48	6,523.85	17.47	25,010	16,853
IntermountainWest	2.12	3.35	2.09	3,799.80	9,500.93	39.99	31,871	19,896
Northern Forest	1.56	1.43	1.15	854.25	3,694.00	23.13	5,293	4,257
Pacific^c^	2.44	3.27	2.55	686.46	1,857.32	36.96	6,082	4,743
Prairie	2.29	3.96	2.15	2,513.31	37,027.83	6.79	146,477	79,478
Southwest^c^					4,419.13			
**All biomes**	**2.15**	**3.35**	**2.10**	**8,993.30**	**63,023.05**	**14.27**		

a Provided by [36], [37].

b See values for all biomes combined in Appendix S9.

c The Dillon Project was the only project in the southwest biome represented by a fatality report that was available. Due to its singularity and since it is located very close to the Pacific biome; it was combined with the Pacific biome data for these estimates. See project characteristics in Appendix S1.

### Effects on Bird Populations

Using the biome-specific rates, the number of fatalities for each species was calculated and summed (Appendix S8). These values were then multiplied by 7% to account for operating capacity in the southwest biome for a total of 229,765 small-passerine fatalities using the bias adjustment value that was lowest and 133,993 with the bias adjustment value that was the highest (Appendix S9), indicating a range of about 134,000 to 230,000 small-passerine fatalities that occur annually in the US and Canada from collisions with turbines.

Using the most conservative estimates, we determined the continent-wide effect from collisions with turbines for each species to be much less than one percent annually, ranging from less than 0.001% to 0.043% (Appendix S10). This means that less than one-tenth of one percent of the continent-wide population for each species is estimated to be killed annually by collisions with wind turbines (Table 6, Appendix S10). For about 20% of all species of small passerines in our study this value was less than 0.001%.

**Table 6:** The percentage and number of individuals of the continental population estimated to be killed by collisions with turbines each year for the top 20 species of small passerines, in comparison to their estimated population sizes, derived from 116 studies of bird collisions at wind energy facilities in the United States and Canada.

Species	Scientific name	#found	% composition	Ave est(lowest value)^b^	Ave est(highest value)^c^	Pop est forNorth America^d^	% pop affected(lowest value)^e^	% pop affected(highest value)^f^
black-throated blue warbler	*Setophaga caerulescens*	27	0.87	895	610	2,100,000	0.043	0.029
tree swallow	*Tachycineta bicolor*	34	1.09	7,390	4,102	17,000,000	0.043	0.024
horned lark	*Eremophila alpestris*	681	21.9	30,591	18,029	80,000,000	0.038	0.023
brown thrasher	*Toxostoma rufum*	3	0.10	1,722	935	4,900,000	0.035	0.019
yellow-throated vireo	*Vireo flavifrons*	4	0.13	1,218	670	3,500,000	0.035	0.019
spotted towhee	*Pipilo maculatus*	7	0.23	716	402	2,200,000	0.033	0.018
sedge wren	*Cistothorus platensis*	3	0.10	1,722	935	6,200,000	0.028	0.015
bushtit	*Psaltriparus minimus*	1	0.03	574	312	2,300,000	0.025	0.014
western meadowlark	*Sturnella neglecta*	159	5.11	6,147	3,790	30,000,000	0.020	0.013
rose-breasted grosbeak	*Pheucticus ludovicianus*	9	0.29	826	486	4,100,000	0.020	0.012
American tree sparrow	*Spizella arborea*	7	0.23	4,019	2,181	20,000,000	0.020	0.011
purple martin	*Progne subis*	3	0.10	1,183	647	6,000,000	0.020	0.011
field sparrow	*Spizella pusilla*	7	0.23	1,324	741	7,600,000	0.017	0.010
grasshopper sparrow	*Ammodramus savannarum*	5	0.16	2,322	1,262	14,000,000	0.017	0.009
brown creeper	*Certhia americana*	10	0.32	1,356	767	8,500,000	0.016	0.009
Bell’s vireo	*Vireo bellii*	1	0.03	574	312	3,600,000	0.016	0.009
barn swallow	*Hirundo rustica*	11	0.35	5,222	2,844	33,000,000	0.016	0.009
Cape May warbler	*Setophaga tigrina*	13	0.42	996	595	7,000,000	0.014	0.009
Le Conte’s sparrow	*Ammodramus leconteii*	2	0.06	1,148	623	8,000,000	0.014	0.008
European starling	*Sturnus vulgaris*	103	3.31	7,892	4,563	57,000,000	0.014	0.008

a includes carcasses found during scheduled carcass searches and incidentally.

b Average estimated number of fatalities each year adjusted by the bias value that was the lowest and for operating capacity (see text and Appendix S9).

c Average estimated number of fatalities each year adjusted by the bias value that was the highest and for operating capacity (see text and Appendix S9).

d Population estimates obtained from the Partners in Flight Landbird Population Estimates Database [29].

e Percent of population affected annually, adjusted by the bias value that was the lowest.

f Percent of population affected annually, adjusted by the bias value that was the highest.

Two values are presented for both percentage and number, representing the extreme values of the range of adjustments applied for each species. Also included are the number of dead birds found and the percent composition by species for the 116 studies. Results for all species are presented in Appendix S10 ^a^.

### An Example

Two fatalities of Acadian flycatcher (*Empidonax virescens*) were documented in all 116 studies; both were found in the eastern biome and represented about 0.26% of all fatalities (762) in that biome (Appendix S8). This composition was multiplied by the two values (based on the adjustments using the bias value that was lowest and the bias value that was highest) for total number of fatalities estimated for the eastern biome based on operating capacity (25,010, and 16,853, respectively; Table 5) to generate two estimates of the annual number of fatalities for Acadian flycatcher from collisions with wind turbines, 66 and 44, respectively. If fatalities of Acadian flycatchers were found in other biomes, values for all biomes would be summed for a continent-wide estimate. To adjust for the absence of data from the southwest biome, all cumulative estimates for species were multiplied by seven percent, which is the proportion of continent-wide operating capacity that the southwest biome represents. This results in estimates of 70 and 47 annual fatalities of Acadian flycatchers, respectively. Finally, the proportion of the North American population of Acadian flycatchers (4.5 million) that these two estimates represent was determined: 0.002% and 0.001%, respectively.

## Discussion

There are factors that make our analysis different from other estimates of rates of bird fatality at wind energy facilities. First, we calculated estimates of small-bird fatality rates for studies where only all-bird estimates were presented. Second, we attempted to remove bias in fatality rate estimation associated with four different estimators, further increasing the accuracy of the continent-wide small-bird fatality rates. Third, our analysis drew upon a much larger set of monitoring efforts at wind energy facilities compared to other similar reviews. Finally, no other study of this type presented fatality rates for small birds both continent-wide and for avifaunal biome regions. All the studies that we included covered the periods of migration for most passerines and used relatively uniform methods of data collection. While we did not incorporate more-specific variables to further refine the effects on bird populations (e.g., population growth rate), we think the population effects we calculated allow comparisons to be made among species of small passerines. Some amount of uncertainty is inherent in all studies on bird mortality related to human activity and it is unclear to what extent these fatalities compensate for deaths from sources not related to humans [38]. Other considerations for assessing the effect of turbine-related mortality include: 1) small passerines most likely make up an even larger percentage of actual fatalities because they are harder to detect than larger birds; 2) as mentioned earlier, passerine species tend to be shorter-lived with higher rates of reproduction than other bird taxa [15], [16]; and 3) even though the location and timing of fatalities may suggest which particular populations for a given species are being affected, this information may be confounded because migrants, breeders, wintering birds, and year-round residents may overlap in their presence at a wind energy facility, particularly for species with large geographical distributions, distinctive migration patterns, and/or unique life history characteristics. Ideally, we would like to address the effects of turbine-related mortality at a more-targeted, regional level. For example, Loss et al. [39] describes regions of catchment as “the portion of a species’ breeding range that is represented by mortalities at a site, assuming straight north-to-south migration” which may be evaluated through geolocator and mark-recapture data. We are not aware of the current availability of this type of data for small passerines in general.

### Other Analyses of Collisions with Turbines and Unknown Factors

By combining the lowest and highest bias-adjusted rates estimated in our analysis for the annual number of small birds killed by turbines (133,993 and 229,765; Table 5) with the proportion of all fatalities that were passerines (62.5%), we calculated that about 214,000 to 368,000 turbine-related deaths occur each year for all birds. These numbers are less than those presented by Smallwood [12] and Manville [10], 573,093 and 440,000, respectively, but slightly more than the values presented by Loss et al. [13], which averaged 234,000 (range: 140,000 and 328,000). Our study differs from that of Smallwood [12] because we used the fatality rate provided in individual studies as opposed to independently recalculating these rates from the raw data using a single common estimator. In addition, we did not use national averages for bias adjustments. We eliminated studies that we believed were inadequate for estimating fatality. Also, we did not correct results based on turbine tower height or search radius in part because there are some confounding factors between the heights of the turbines and the size of the rotor swept area. We were unable to determine how Manville [10] calculated his estimate.

There are factors that may influence any per year estimate of bird fatalities. First, taller turbines may be related to an increase in bird mortality, as suggested by Loss et al. [13]. We found no linear correlation between turbine tower height and the fatality rates we estimated, but other factors such as geographic location or turbine age may confound the effect of tower height. Second, the size of the search plot may influence how many actual fatalities are found by searchers. Guidelines provided by the USFWS recommend that search plots for birds have a width that is twice the length of the turbine tower height to the ground [1], while another recommendation is that the length of the radius of search plots for birds match the height of the highest point of the rotor swept area of the turbine (the area that the blades pass over when moving), which may be about 90–120 m or more for modern turbines [14]. Not all plot sizes in studies for our analysis conformed to these guidelines, and some studies may underestimate the number of bird fatalities found due to the bird carcasses landing outside the search plot. Smallwood [12] and Loss et al. [13] both adjusted their estimate of bird fatalities per year based on the proportion of all fatalities found for classes of turbine tower height paired with plot size derived by Smallwood [12] from raw data contained in previous studies. While we recognize this as a potential bias, we also recognize that background mortality (evidence of carcasses found that are not caused by collision with wind turbines) is likely an important positive bias, and limited studies suggest this bias may partially or even completely offset any bias associated with plot size. Background mortality is an issue with these studies because observers may incorrectly identify a carcass found within the survey area as a fatality caused by colliding with a turbine when it actually may have died from another cause. We found two studies on this phenomenon (one conducted in Tennessee and one in Minnesota) in which plots away from turbines were monitored simultaneously with regular plots at turbines. For these, researchers determined the rate of background mortality as 0.22 and 1.10 birds/reference plot/year for circular plots with a 50-m radius and 126 m by 126 m plots, respectively [40], [41], which accounted for about 4% or 33% of the total estimated fatality. Additional evidence of background mortality was found during a study in Montana for a proposed wind energy facility, where four dead birds were found along linear transects totaling a distance of about 290 km over a two-year period [42]. The extent of background mortality, while not known, is likely an important bias in estimating turbine collision fatalities and probably varies according to location. In addition, assuming background mortality is proportional to area searched; larger plots that are searched would be expected to have more mortality associated with factors independent of collision. This suggests the plot size models of Smallwood [12] may have an increasing bias with increasing plot size. Zimmerling et al. [43] estimated 23,300 bird deaths/year in Canada (8.2±1.4 fatalities/turbine/year) based on 2,955 operating turbines from studies at 43 wind projects in eight provinces in Canada. The authors stated that in Canada passerines typically comprise 80% of all fatalities at turbines and they found population effects of less than 0.01% for species of small passerines that made up the most overall fatalities. Unlike other analyses discussed here, the authors appear to have applied a single set of correction factors to all carcasses regardless of size and search conditions, making comparisons to other studies questionable (M. Huso, personal communication).

To explore the potential greater effect of older-generation facilities on small-passerine fatalities [44] we estimated potential fatalities from older generation turbines that are currently in operation, based on our overall estimate. We know of three current facilities with older generation turbines: Altamont Pass, San Gorgonio, and Tehachapi, all located in California. About 317 MW of 454 total MW at Altamont Pass are from older turbines [45]. At San Gorgonio about 300 MW out of about 550 total MW are from older turbines [46], and about 800 MW of capacity from a total of over 3,000 MW are estimated to be derived from older turbines at Tehachapi [47]. The combined total, 1,417 MW, is about 2.25% of the continent-wide operating capacity in our analysis (Table 5). Smallwood and Karas [48] compared fatality rates at Altamont between modernized (repowered) and older turbines, finding notable reductions at the repowered facilities compared to older generation. For horned lark, loggerhead shrike, and western meadowlark, the authors noted reductions of 83%, 44%, and 44%, respectively. Applying the highest reduction in their study to the 2.25% of capacity from older generation turbines in our study results in a fatality rate of roughly 30,400 small-passerine fatalities per year. Adding this value to our most conservative rate of 225,000 (after adjusting for the remaining 97.75% of total capacity from modern turbines) results in a total of 255,000 total small-passerine fatalities per year. When extrapolated to all birds this results in a total of 408,000 all-bird fatalities per year.

### Comparison to Other Sources of Avian Fatalities

Longcore et al. [22] reported on species composition for birds that collide with communication towers. They documented over a quarter-million bird fatalities consisting of 239 bird species, of which about 97% were passerines (corvids made up 0.01% of all fatalities while our study excluded large corvids), and over half of these were warblers. These fatalities were found at 107 communication tower locations in central and eastern North America. In a similar study, Longcore et al. [21] stated that 6.8 million birds die annually from collisions with communication towers in the United States and Canada. The authors derived this number from 38 studies of communication towers such as cellular towers, television towers, radio towers, microwave towers, and public safety communication system towers. Adjustments for searcher efficiency and scavenger bias were made. Combining these findings indicates that about 6.6 million passerines are killed by communication towers per year. Annual mortality from communication towers was estimated to be at least one percent of the total population size for 26 passerine species. The estimated effect on populations from wind turbines is far less than for communication towers, which ranged up to nine percent/year and was at least one percent for 29 species of birds [22]. In our analysis two of the top 20 species for population effects were warblers (Table 6), while about half of the top 20 species were warblers in the other study.

An estimated 1.4 to 3.7 billion bird fatalities per year were attributed to cats in the contiguous US [49]. Two-thirds of these fatalities were linked to cats that were not owned by people as pets (e.g., barn cats and feral cats). In Canada 100–350 million bird deaths were estimated to be caused by predation by cats [50].

In Canada 269 million bird deaths each year were estimated to be from human-related sources; over 95% of these were attributed to predation by cats and collisions with road vehicles, houses, and transmission lines, while annual mortality from wind turbines was estimated to be 23,300 birds [51], [43].

### Passerine Species

Our analysis shows the species composition for small passerines whose populations will most likely be affected by collisions with turbines (Table 6, Appendix S10). In general, a species with a small population size and high numbers of casualties from collisions with turbines would be affected the most, and a species with a large population would be influenced much less, particularly when the number of casualties is small.

In our study the continent-wide population of the black-throated blue warbler (*Dendroica caerulescens*) was affected the most in comparison to other species of small passerines (0.029%–0.043% annual loss due to collisions with turbines). In comparison, the effect from communications towers presented by Longcore et al. [22] for this species was 4.9%, more than two orders of magnitude higher. Interestingly, Arnold and Zink [52] identified the black-throated blue warbler as the one that collides most frequently with towers and buildings. However, these authors have been criticized for concluding that bird population trends were not affected by collisions because the authors did not adjust for other population factors and had a limited dataset [53], [54], [22]. In contrast, horned lark comprised the highest proportion of small-passerine fatalities in our data, at least twice as many fatalities as any other species, but the estimated population effect ranged from 0.023% to 0.038%, less than that of the black-throated blue warbler, because of the large size of the continental population for horned lark.

### Passerine Families

Of the passerine families with the most fatalities, a single species was sometimes disproportionally represented, even though several species are members of the taxa in North America. For example, about 82.3% of collisions in Vireonidae were red-eyed vireos, and 52.6% of found fatalities of Icteridae were western meadowlarks. Regulidae is comprised of two species in North America, and of these the golden-crowned kinglet made up 71.5% of fatalities for that family (Table 3 and Appendix S9). Parulidae comprised a much smaller percent of overall fatalities with wind turbines (10.8%) as compared to communication towers (58.4%;), while a similar percent of fatalities from Emberizidae were killed by turbines (6.0%) compared to communication towers (5.8%) [22].

### Timing of Fatalities

Fatalities for most small-passerine families – notably Hirundinidae (swallows**)**, Turdidae (thrushes), Vireonidae, and Parulidae - followed a pattern where most fatalities occurred in fall followed by a smaller peak in spring (Figures S2–S27). For Emberizidae and Alaudidae combined, a bird grouping typically assessed in biological studies for wind energy facilities, spring was the time of most collisions overall. Male horned larks sing while flying at heights up to 250 meters during courtship displays in spring [55] and could encounter blades from wind turbines during their performance. This demonstrates how effects to small passerines cannot be generalized, but perhaps should be assessed on a species level or by the suite of species associated with the type of habitat present, depending on the study.

### Species of Conservation Concern

Species of conservation concern [56] identified as fatalities in our analysis included bay-breasted warbler (*Setophaga castanea*), Bell’s vireo (*Vireo bellii*), Bewick’s wren (*Thryomanes bewickii*), blue-winged warbler (*Vermivora cyanoptera*), Canada warbler (*Cardellina canadensis*), cerulean warbler (*Setophaga cerulea*), dickcissel (*Spiza americana*), gray vireo (*Vireo vicinior*), Kentucky warbler (*Geothlypis formosa*), loggerhead shrike (*Lanius ludovicianus*), prairie warbler (*Setophaga discolor*), tricolored blackbirds (*Agelaius tricolor*) and wood thrush (*Hylocichla mustelina*). Continental populations for all of these species were estimated to be affected by 0.016% or less (Appendix S10).

### Biome-level Rates

Adjusted fatality rates for small birds at wind energy facilities for each biome ranged from 1.43 birds/MW/year in the northern forest biome to 3.96 in the prairie biome, based on the most conservative bias-adjusted rates (Table 5). The ability to discuss turbine-related fatalities at a biome level may contribute to coordinated, cooperative conservation planning and management efforts, at the very minimum by providing an understanding of the actual measured and bias-corrected effect of wind turbines on populations of small passerines. Comparison of rates between regions may not be as useful due to differences in the availability of habitats and the quantity and geographic location of studies among regions. In many cases it is also not possible to determine whether an individual killed by a turbine was breeding in that area, migrating through it, or wintering there.

Our analysis indicated that fatalities from collisions with turbines are fewer than fatalities from other anthropogenic sources, including individual and cumulative effects to listed sensitive species of small passerines. Those species protected by the Migratory Bird Treaty Act [57], which includes passerine species, are likely to continue to be of concern to federal and state regulatory agencies, the wind industry, and other stakeholders.

### Further Research

Our work is a major advance in assessing the accumulated data available from many individual fatality monitoring studies, and provides preliminary insight into the effects of wind energy turbines on populations of small passerines. As more studies are conducted and their results made available, they will help refine the current understanding overall and at the level of avifaunal biomes, which will contribute to a better ability to make decisions about effects, turbine siting, and possible avoidance and mitigation strategies. This analysis and future analyses of these data can provide information that could be used to better predict small passerine mortality on future projects, and focus monitoring efforts on more specific unanswered questions or possibly avoidance and mitigation. Our analysis suggests overall mortality of small passerines from wind energy is minor compared to passerine mortality from other anthropogenic sources, and development of solutions for reducing mortality from those other sources may provide more benefit to passerine populations than concentrating efforts on reducing impacts of wind energy. Efforts towards understanding the consequence of wind energy on small passerines should focus primarily on understanding effects on listed species as well as other species of concern such as birds that breed in grasslands.

All appropriate data were included in our analysis, but for some areas of the continent only limited data were available. For example, only 7% of the installed capacity of the prairie biome was included in this study. As mentioned above, only one report was available to us that represented the southwest biome, even though this region contains over 4,400 MW of operating capacity. Additional insight could be gained on the effect of wind energy on small passerines in this biome as more studies are conducted and become available.

## Supporting Information

Figure S1 **Regression between calculated estimates of small-bird fatality rates and reported small-bird rates for studies at wind energy facilities in the United States and Canada for which both all-bird and small-bird estimates are provided.**
(TIF)

Figure S2 **The monthly timing of fatalities for blackbirds and orioles (Icteridae) from 79 fatality studies for which the date when each fatality was found was provided.** Timing of fatalities for small passerine families (presented alphabetically), unidentified passerines, and species of interest for 79 of 116 fatality monitoring studies in the United States and Canada for which dates were provided.
(TIF)

Figure S3 **The monthly timing of fatalities for small corvids (Corvidae) from 79 fatality studies for which the date when each fatality was found was provided.** Timing of fatalities for small passerine families (presented alphabetically), unidentified passerines, and species of interest for 79 of 116 fatality monitoring studies in the United States and Canada for which dates were provided.
(TIF)

Figure S4 **The monthly timing of fatalities for creepers and nuthatches (Certhiidae and Sittidae) from 79 fatality studies for which the date when each fatality was found was provided.** Timing of fatalities for small passerine families (presented alphabetically), unidentified passerines, and species of interest for 79 of 116 fatality monitoring studies in the United States and Canada for which dates were provided.
(TIF)

Figure S5 **The monthly timing of fatalities for finches and crossbills (Fringillidae) from 79 fatality studies for which the date when each fatality was found was provided.** Timing of fatalities for small passerine families (presented alphabetically), unidentified passerines, and species of interest for 79 of 116 fatality monitoring studies in the United States and Canada for which dates were provided.
(TIF)

Figure S6 **The monthly timing of fatalities for flycatchers (Tyrannidae) from 79 fatality studies for which the date when each fatality was found was provided.** Timing of fatalities for small passerine families (presented alphabetically), unidentified passerines, and species of interest for 79 of 116 fatality monitoring studies in the United States and Canada for which dates were provided.
(TIF)

Figure S7 **The monthly timing of fatalities for gnatcatchers and kinglets (Polioptilidae and Regulidae) from 79 fatality studies for which the date when each fatality was found was provided.** Timing of fatalities for small passerine families (presented alphabetically), unidentified passerines, and species of interest for 79 of 116 fatality monitoring studies in the United States and Canada for which dates were provided.
(TIF)

Figure S8 **The monthly timing of fatalities for grassland species and sparrows (Alaudidae/Emberizidae) from 79 fatality studies for which the date when each fatality was found was provided.** Timing of fatalities for small passerine families (presented alphabetically), unidentified passerines, and species of interest for 79 of 116 fatality monitoring studies in the United States and Canada for which dates were provided.
(TIF)

Figure S9 **The monthly timing of fatalities for mimids (Mimidae) from 79 fatality studies for which the date when each fatality was found was provided.** Timing of fatalities for small passerine families (presented alphabetically), unidentified passerines, and species of interest for 79 of 116 fatality monitoring studies in the United States and Canada for which dates were provided.
(TIF)

Figure S10 **The monthly timing of fatalities for shrikes (Laniidae) from 79 fatality studies for which the date when each fatality was found was provided.** Timing of fatalities for small passerine families (presented alphabetically), unidentified passerines, and species of interest for 79 of 116 fatality monitoring studies in the United States and Canada for which dates were provided.
(TIF)

Figure S11 **The monthly timing of fatalities for swallows (Hirundinidae) from 79 fatality studies for which the date when each fatality was found was provided.** Timing of fatalities for small passerine families (presented alphabetically), unidentified passerines, and species of interest for 79 of 116 fatality monitoring studies in the United States and Canada for which dates were provided.
(TIF)

Figure S12 **The monthly timing of fatalities for tanagers, grosbeaks, and cardinals (Cardinalidae), from 79 fatality studies for which the date when each fatality was found was provided.** Timing of fatalities for small passerine families (presented alphabetically), unidentified passerines, and species of interest for 79 of 116 fatality monitoring studies in the United States and Canada for which dates were provided.
(TIF)

Figure S13 **The monthly timing of fatalities for thrushes (Turdidae) from 79 fatality studies for which the date when each fatality was found was provided.** Timing of fatalities for small passerine families (presented alphabetically), unidentified passerines, and species of interest for 79 of 116 fatality monitoring studies in the United States and Canada for which dates were provided.
(TIF)

Figure S14 T**he monthly timing of fatalities for titmice and chickadees (Paridae) from 79 fatality studies for which the date when each fatality was found was provided.** Timing of fatalities for small passerine families (presented alphabetically), unidentified passerines, and species of interest for 79 of 116 fatality monitoring studies in the United States and Canada for which dates were provided.
(TIF)

Figure S15 **The monthly timing of fatalities for vireos (Vireonidae) from 79 fatality studies for which the date when each fatality was found was provided.** Timing of fatalities for small passerine families (presented alphabetically), unidentified passerines, and species of interest for 79 of 116 fatality monitoring studies in the United States and Canada for which dates were provided.
(TIF)

Figure S16 **The monthly timing of fatalities for warblers (Parulidae) from 79 fatality studies for which the date when each fatality was found was provided.** Timing of fatalities for small passerine families (presented alphabetically), unidentified passerines, and species of interest for 79 of 116 fatality monitoring studies in the United States and Canada for which dates were provided.
(TIF)

Figure S17 **The monthly timing of fatalities for waxwings (Bombycillidae) from 79 fatality studies for which the date when each fatality was found was provided.** Timing of fatalities for small passerine families (presented alphabetically), unidentified passerines, and species of interest for 79 of 116 fatality monitoring studies in the United States and Canada for which dates were provided.
(TIF)

Figure S18 **The monthly timing of fatalities for wrens (Troglodytidae) from 79 fatality studies from which the date when each fatality was found was provided.** Timing of fatalities for small passerine families (presented alphabetically), unidentified passerines, and species of interest for 79 of 116 fatality monitoring studies in the United States and Canada for which dates were provided.
(TIF)

Figure S19 T**he monthly timing of fatalities for all unidentified passerines from 79 fatality studies for which the date when each fatality was found was provided.** Timing of fatalities for small passerine families (presented alphabetically), unidentified passerines, and species of interest for 79 of 116 fatality monitoring studies in the United States and Canada for which dates were provided.
(TIF)

Figure S20 **The monthly timing of fatalities for the bay-breasted warbler (** ***Setophaga castanea*** **) from 79 fatality studies for which the date when each fatality was found was provided.** Timing of fatalities for small passerine families (presented alphabetically), unidentified passerines, and species of interest for 79 of 116 fatality monitoring studies in the United States and Canada for which dates were provided.
(TIF)

Figure S21 **The monthly timing of fatalities for the black-throated blue warbler (** ***Setophaga caerulescens*** **) from 79 fatality studies for which the date when each fatality was found was provided.** Timing of fatalities for small passerine families (presented alphabetically), unidentified passerines, and species of interest for 79 of 116 fatality monitoring studies in the United States and Canada for which dates were provided.
(TIF)

Figure S22 **The monthly timing of fatalities for the blue-winged warbler (** ***Vermivora cyanoptera*** **) from 79 fatality studies for which the date when each fatality was found was provided.** Timing of fatalities for small passerine families (presented alphabetically), unidentified passerines, and species of interest for 79 of 116 fatality monitoring studies in the United States and Canada for which dates were provided.
(TIF)

Figure S23 **The monthly timing of fatalities for the Canada warbler (** ***Cardellina canadensis*** **) from 79 fatality studies for which the date when each fatality was found was provided.** Timing of fatalities for small passerine families (presented alphabetically), unidentified passerines, and species of interest for 79 of 116 fatality monitoring studies in the United States and Canada for which dates were provided.
(TIF)

Figure S24 **The monthly timing of fatalities for the golden-crowned kinglet (** ***Regulus satrapa*** **) from 79 fatality studies for which the date when each fatality was found was provided.** Timing of fatalities for small passerine families (presented alphabetically), unidentified passerines, and species of interest for 79 of 116 fatality monitoring studies in the United States and Canada for which dates were provided.
(TIF)

Figure S25 **The monthly timing of fatalities for the horned lark (** ***Eremophila alpestris*** **) from 79 fatality studies for which the date when each fatality was found was provided.** Timing of fatalities for small passerine families (presented alphabetically), unidentified passerines, and species of interest for 79 of 116 fatality monitoring studies in the United States and Canada for which dates were provided.
(TIF)

Figure S26 **The monthly timing of fatalities for the red-eyed vireo (** ***Vireo olivaceus*** **) from 79 fatality studies for which the date when each fatality was found was provided.** Timing of fatalities for small passerine families (presented alphabetically), unidentified passerines, and species of interest for 79 of 116 fatality monitoring studies in the United States and Canada for which dates were provided.
(TIF)

Figure S27 **The monthly timing of fatalities for the western meadowlark (** ***Sturnella neglecta*** **) from 79 fatality studies for which the date when each fatality was found was provided.** Timing of fatalities for small passerine families (presented alphabetically), unidentified passerines, and species of interest for 79 of 116 fatality monitoring studies in the United States and Canada for which dates were provided.
(TIF)

Appendix S1 **Description of fatality studies conducted at wind energy facilities, including project location, number of turbines, size (megawatts [MW]), and height of turbines; and post-construction study information, divided into avifaunal biome regions for the United States and Canada.** Blank spaces indicate that data was not available.
(DOCX)

Appendix S2 **Bird fatality monitoring studies at wind energy facilities with estimates of fatality rates for small birds, confidence intervals (CI) and estimator used, if known, for avifaunal biomes in the United States and Canada.** Blank spaces indicate that data was not available.
(DOCX)

Appendix S3 **Variables used to calculate a multiplier value used to determine estimates of small-bird rates of fatality for wind energy fatality studies providing only all-bird estimates, in the United States and Canada, grouped by avifaunal biome.**
(DOCX)

Appendix S4 **Percent of trial carcasses found in experimental searcher efficiency (SEEF) bias trials and average removal time (days) in carcass removal (CRT) bias trials for small birds and all birds for post-construction fatality monitoring studies conducted at wind energy facilities in the United States and Canada, categorized by avifaunal biome.** Blank spaces indicate that data was not available.
(DOCX)

Appendix S5 **Additional variables used to calculate a multiplier value used to determine estimated rates of fatality for small birds and the calculated multiplier for wind energy fatality studies providing only all-bird estimates for each associated avifaunal biome in the United States and Canada.** See equation in methods section of main document.
(DOCX)

Appendix S6 **Variables used to determine which bias adjustment factors to apply to the fatality estimate for small birds in 116 studies of bird collisions at wind energy facilities conducted in the United States and Canada.** Search interval, percent of small birds found during searcher efficiency trials and categorization [low (0–0.375), medium (0.375–0.65), and high (0.65–1)], average carcass removal times (days) and carcass removal classification [fast (0–10 days), moderate (11–23 days), and slow (24 or more days)].
(DOCX)

Appendix S7 **Fatality studies conducted at wind energy facilities in the United States and Canada, sorted by avifaunal biome region, with associated small-bird fatality rate estimates (birds/megawatt/year), for which the estimator type is identified, the applied low and high bias adjustment factors, followed by the resulting low and high bias-adjusted fatality rates for small birds.**
(DOCX)

Appendix S8 **The number and percent composition of fatalities of small passerines found during 116 studies of bird collisions with wind energy turbines, geographically separated into avifaunal biomes, along with the estimated annual number of fatalities using the lowest and highest bias adjustment value.** Blank spaces indicate no fatalities were recorded. See Appendix S10 for scientific names of each species. Column A = number of fatalities found in studies in biome, B = % composition in biome, C = estimated number of fatalities each year adjusted by the bias value that was the lowest, D = estimated number of fatalities each year adjusted by the bias value that was the highest.
(DOCX)

Appendix S9 The cumulative values for analysis of small-passerine fatalities in 116 studies at wind energy facilities from all associated avifaunal biomes* in the United States and Canada. Total number and percent composition of fatalities of small-passerines, estimated number of fatalities using for the lowest and highest value of bias and the biome-specific rate (bird fatalities/megawatt/year) from studies and total megawatts of operating capacity, and the adjusted number of fatalities by species.
(DOCX)

Appendix S10 **Annual fatality rates compared to population sizes for species of small passerines found as fatalities in 116 available studies conducted at wind energy facilities in the United States and Canada.** The estimated average number and percent of population killed each year after low and high bias adjustments were applied and adjusted for operating capacity (see Appendix S9); estimated population size, and the proportion of the population that these estimates represent. Based on actual fatalities found both during scheduled searches and incidentally.
(DOCX)
