# Supplementary figures and images for: A Comprehensive Analysis of Small-Passerine Fatalities from Collision with Turbines at Wind Energy Facilities
Source: PLoS One. 2014 Sep 15;9(9):e107491. doi: 10.1371/journal.pone.0107491 (PMC4164633; doi:10.1371/journal.pone.0107491)

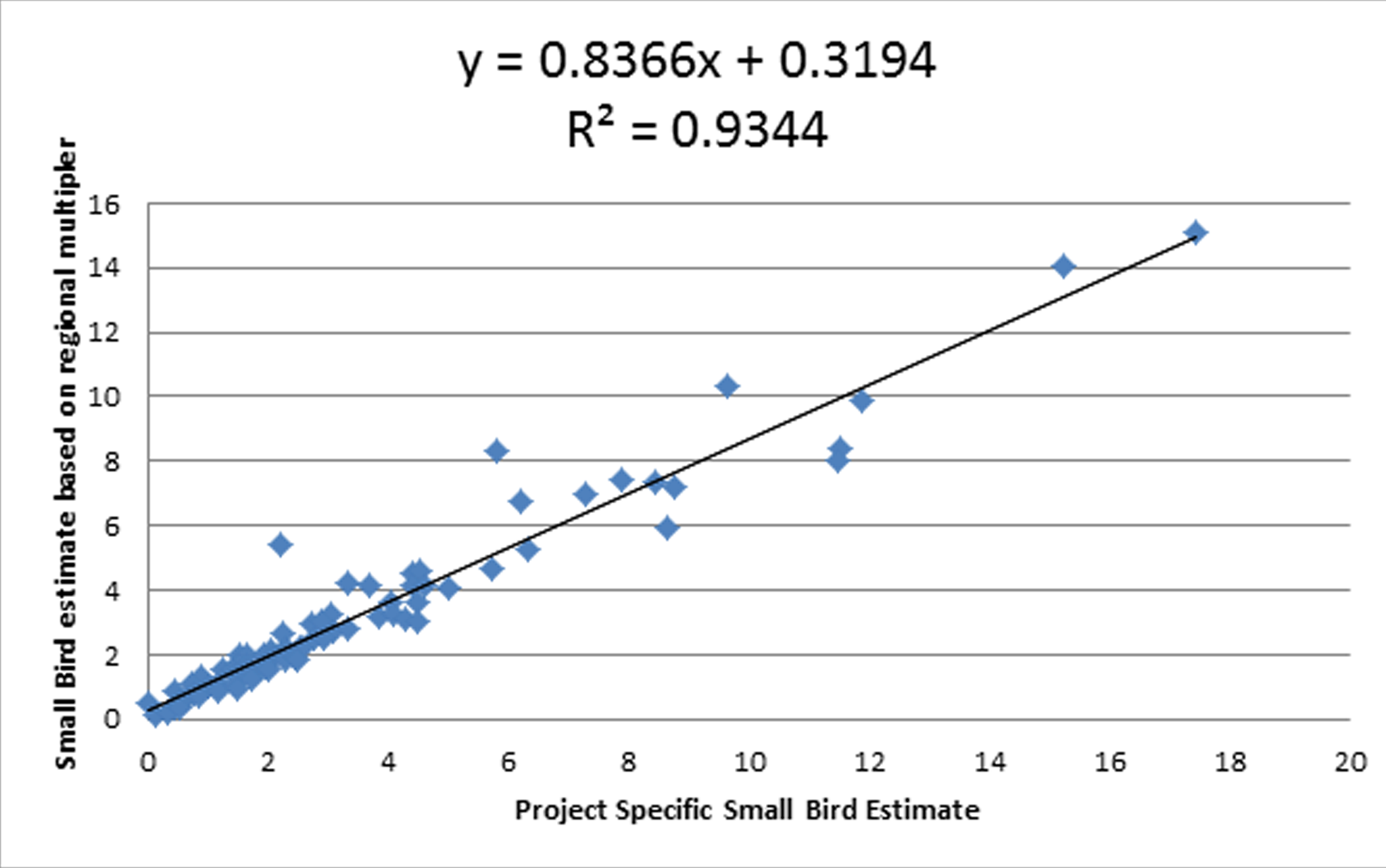

Supplement: Figure S1 — Regression between calculated estimates of small-bird fatality rates and reported small-bird rates for studies at wind energy facilities in the United States and Canada for which both all-bird and small-bird estimates are provided. (TIF) [file pone.0107491.s001.tif]

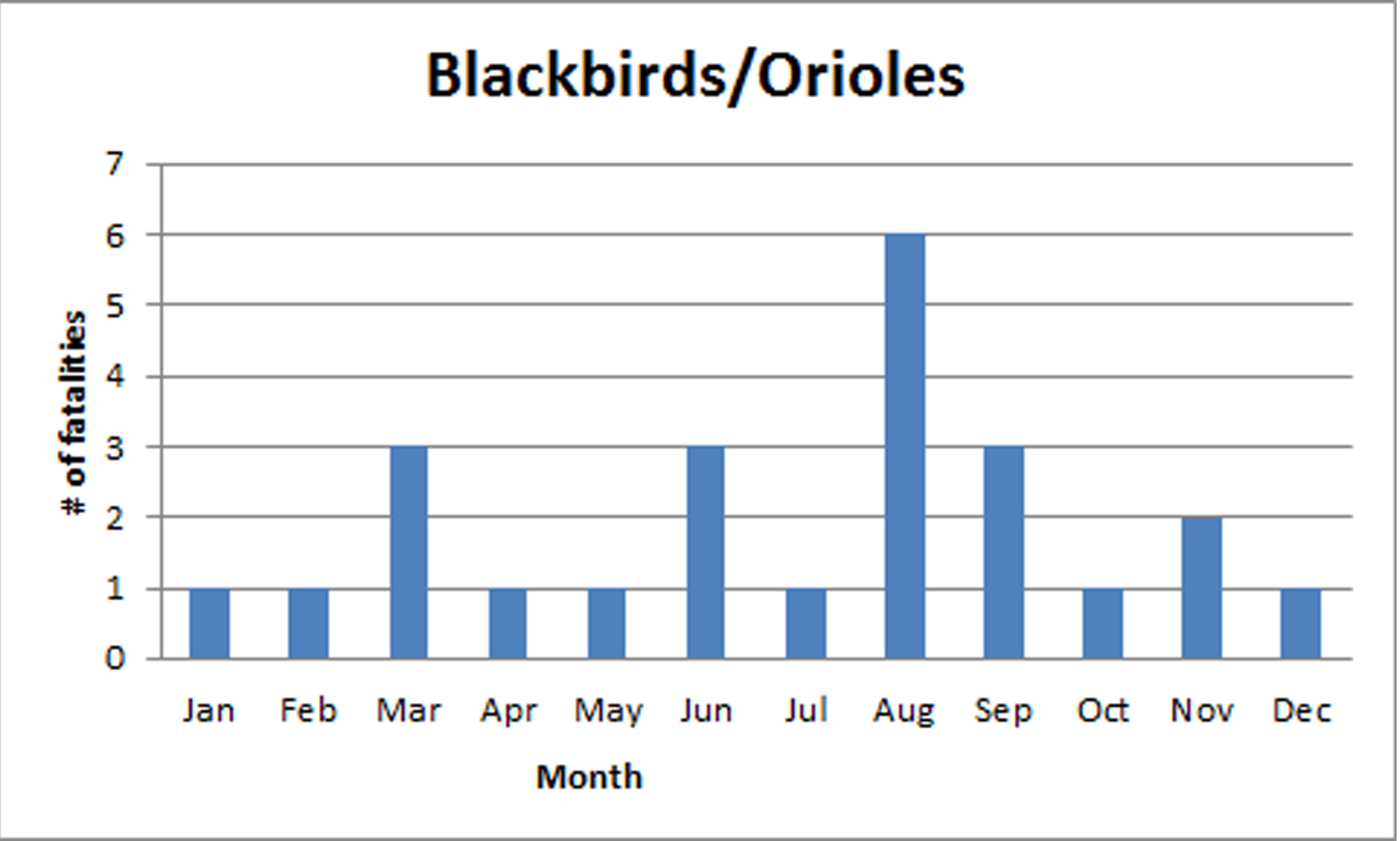

Supplement: Figure S2 — The monthly timing of fatalities for blackbirds and orioles (Icteridae) from 79 fatality studies for which the date when each fatality was found was provided. Timing of fatalities for small passerine families (presented alphabetically), unidentified passerines, and species of interest for 79 of 116 fatality monitoring studies in the United States and Canada for which dates were provided. (TIF) [file pone.0107491.s002.tif]

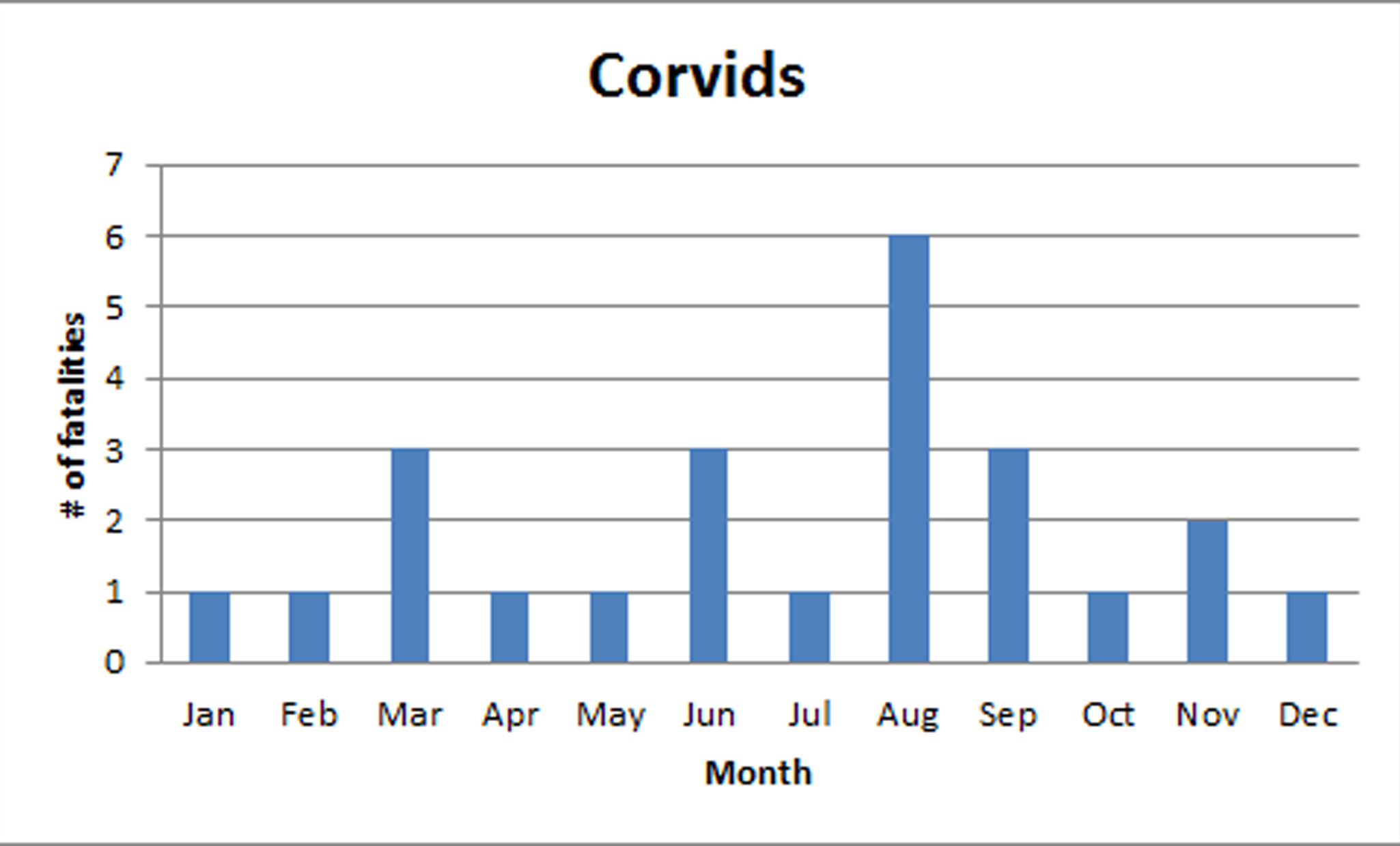

Supplement: Figure S3 — The monthly timing of fatalities for small corvids (Corvidae) from 79 fatality studies for which the date when each fatality was found was provided. Timing of fatalities for small passerine families (presented alphabetically), unidentified passerines, and species of interest for 79 of 116 fatality monitoring studies in the United States and Canada for which dates were provided. (TIF) [file pone.0107491.s003.tif]

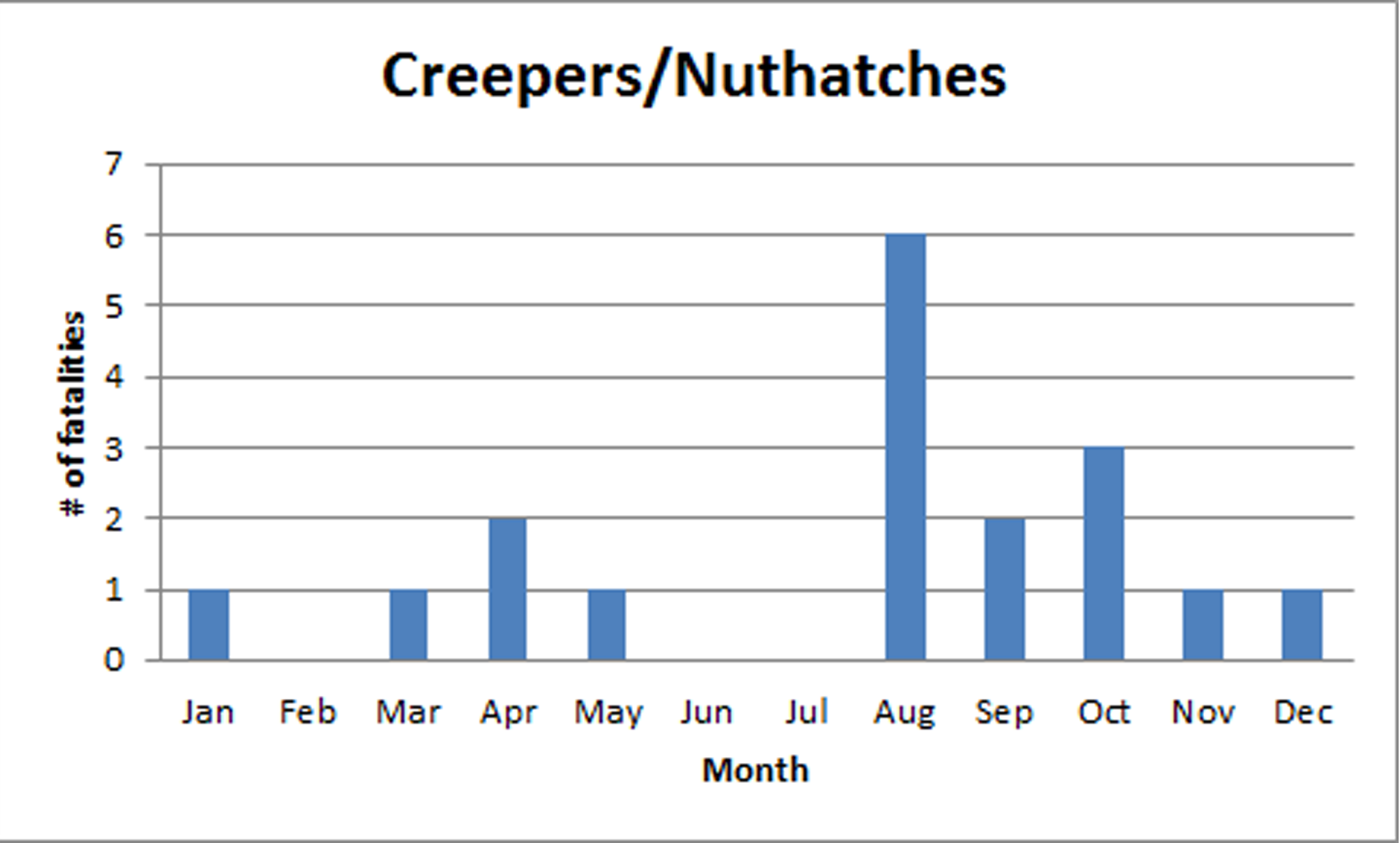

Supplement: Figure S4 — The monthly timing of fatalities for creepers and nuthatches (Certhiidae and Sittidae) from 79 fatality studies for which the date when each fatality was found was provided. Timing of fatalities for small passerine families (presented alphabetically), unidentified passerines, and species of interest for 79 of 116 fatality monitoring studies in the United States and Canada for which dates were provided. (TIF) [file pone.0107491.s004.tif]

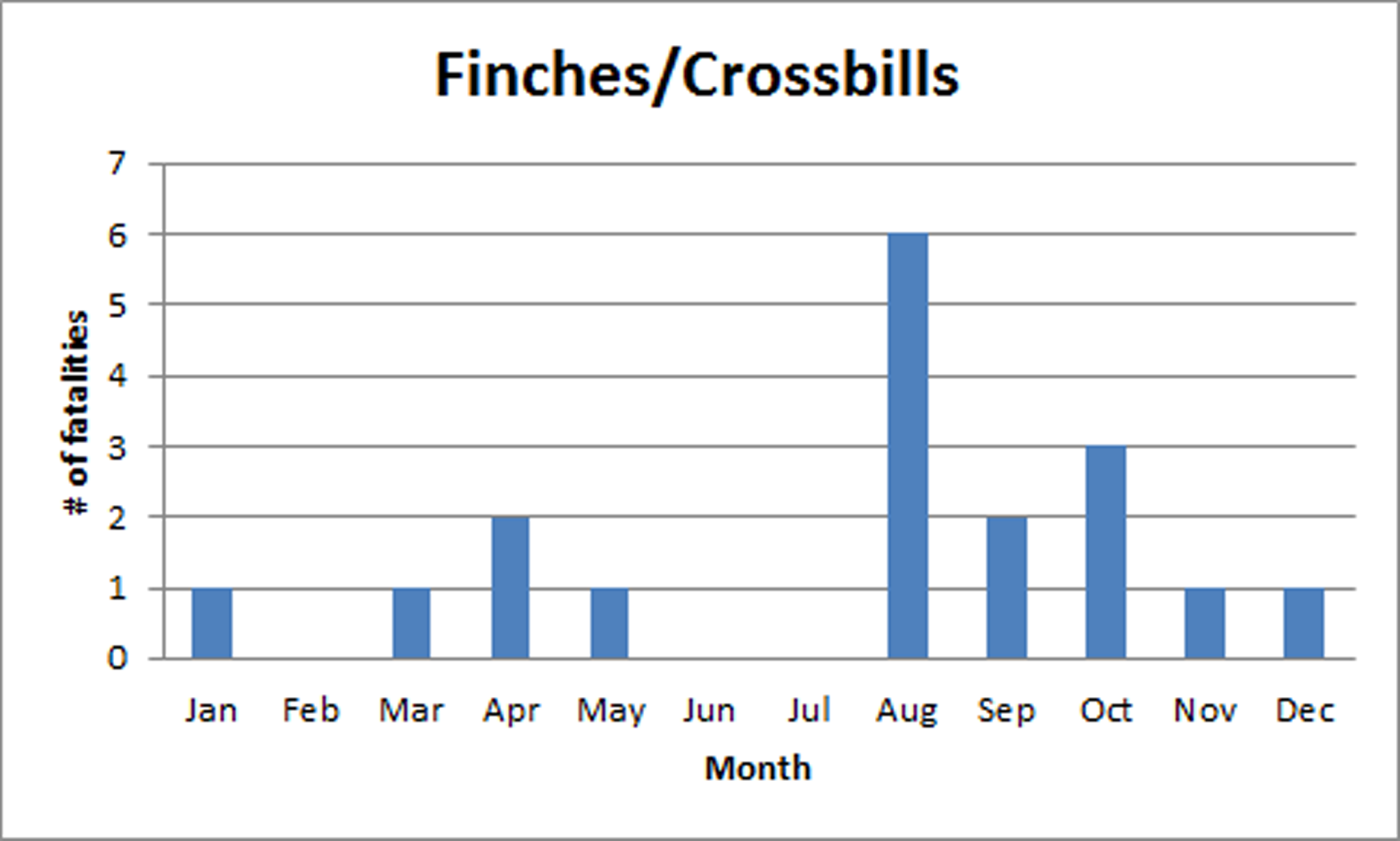

Supplement: Figure S5 — The monthly timing of fatalities for finches and crossbills (Fringillidae) from 79 fatality studies for which the date when each fatality was found was provided. Timing of fatalities for small passerine families (presented alphabetically), unidentified passerines, and species of interest for 79 of 116 fatality monitoring studies in the United States and Canada for which dates were provided. (TIF) [file pone.0107491.s005.tif]

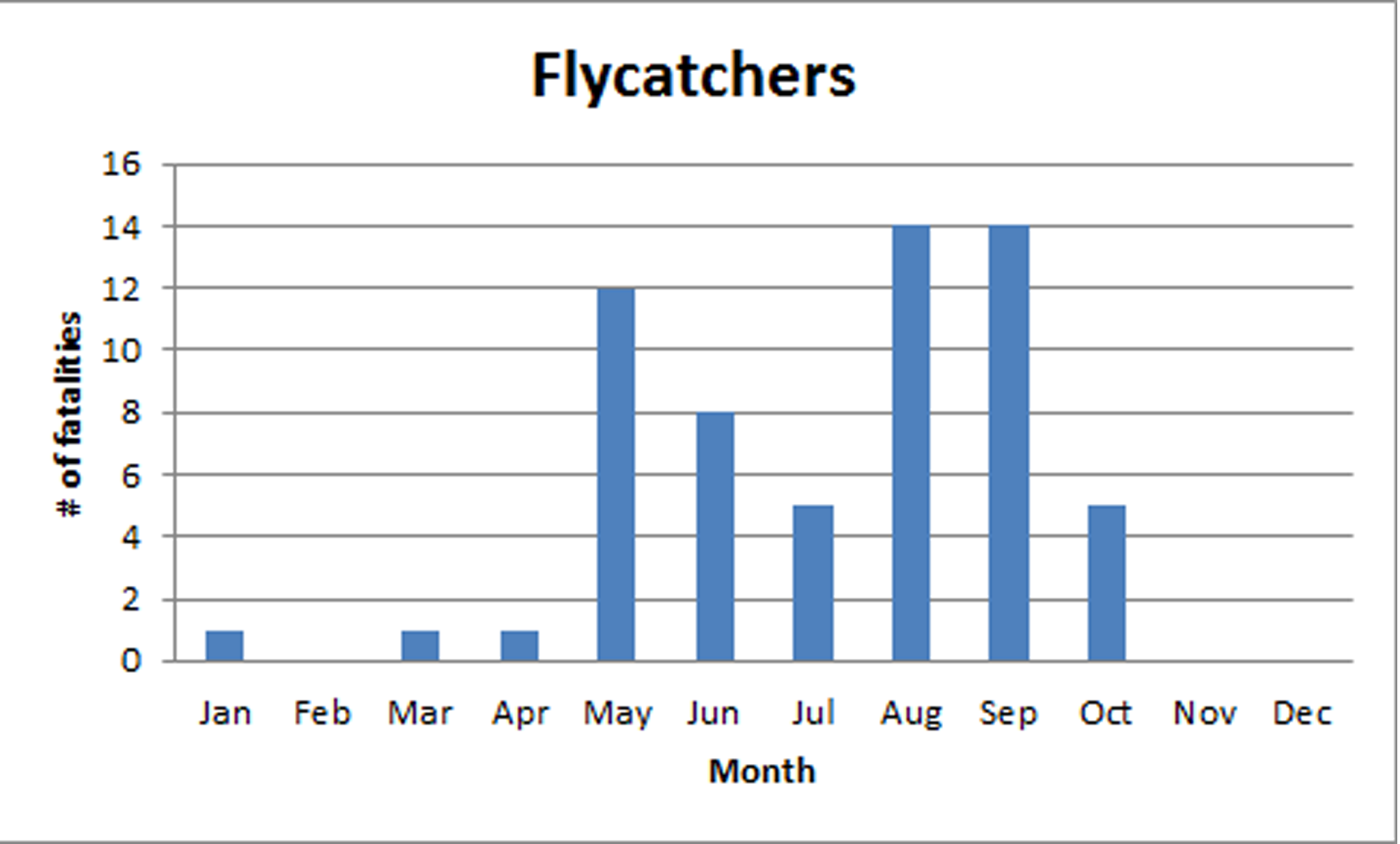

Supplement: Figure S6 — The monthly timing of fatalities for flycatchers (Tyrannidae) from 79 fatality studies for which the date when each fatality was found was provided. Timing of fatalities for small passerine families (presented alphabetically), unidentified passerines, and species of interest for 79 of 116 fatality monitoring studies in the United States and Canada for which dates were provided. (TIF) [file pone.0107491.s006.tif]

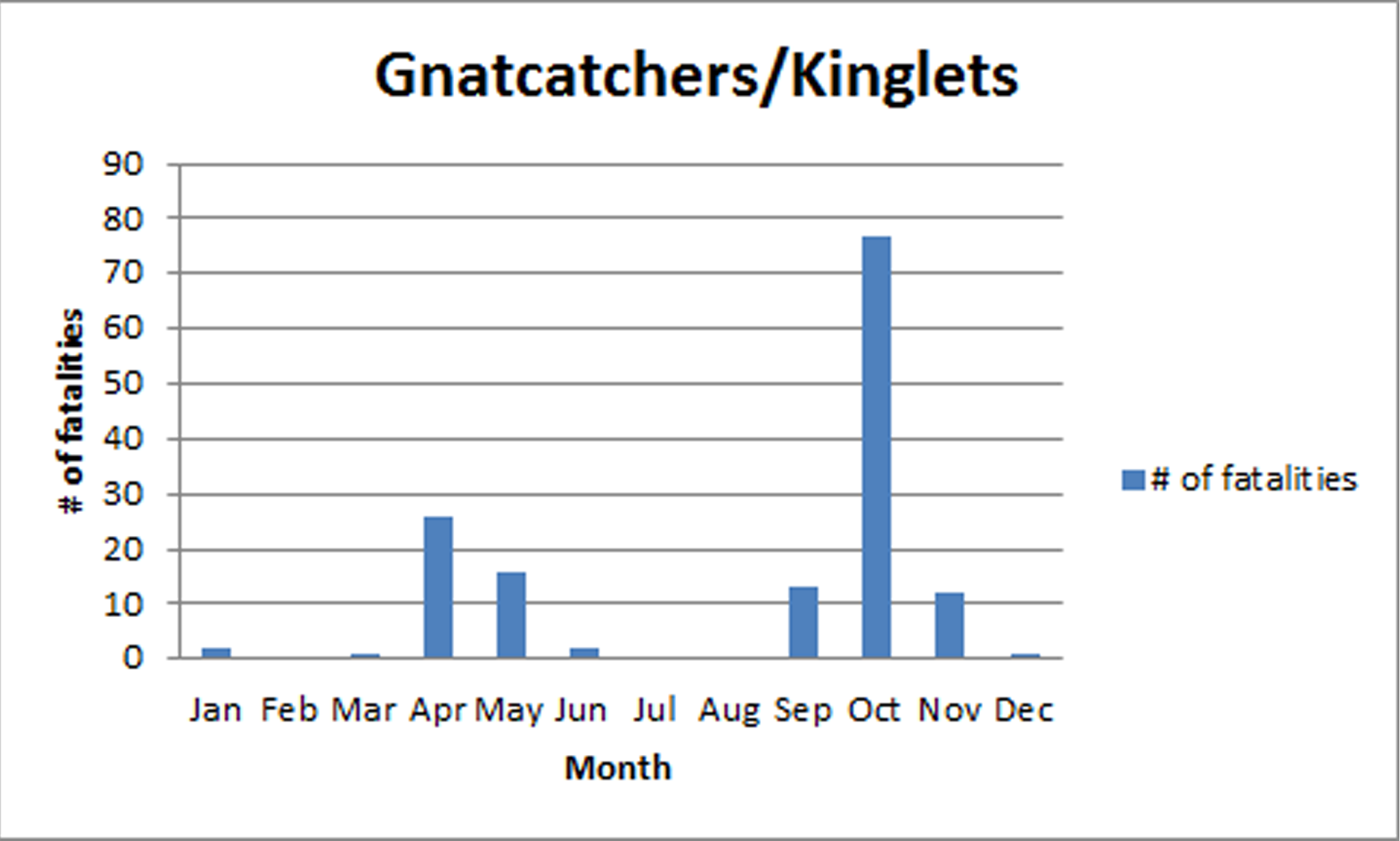

Supplement: Figure S7 — The monthly timing of fatalities for gnatcatchers and kinglets (Polioptilidae and Regulidae) from 79 fatality studies for which the date when each fatality was found was provided. Timing of fatalities for small passerine families (presented alphabetically), unidentified passerines, and species of interest for 79 of 116 fatality monitoring studies in the United States and Canada for which dates were provided. (TIF) [file pone.0107491.s007.tif]

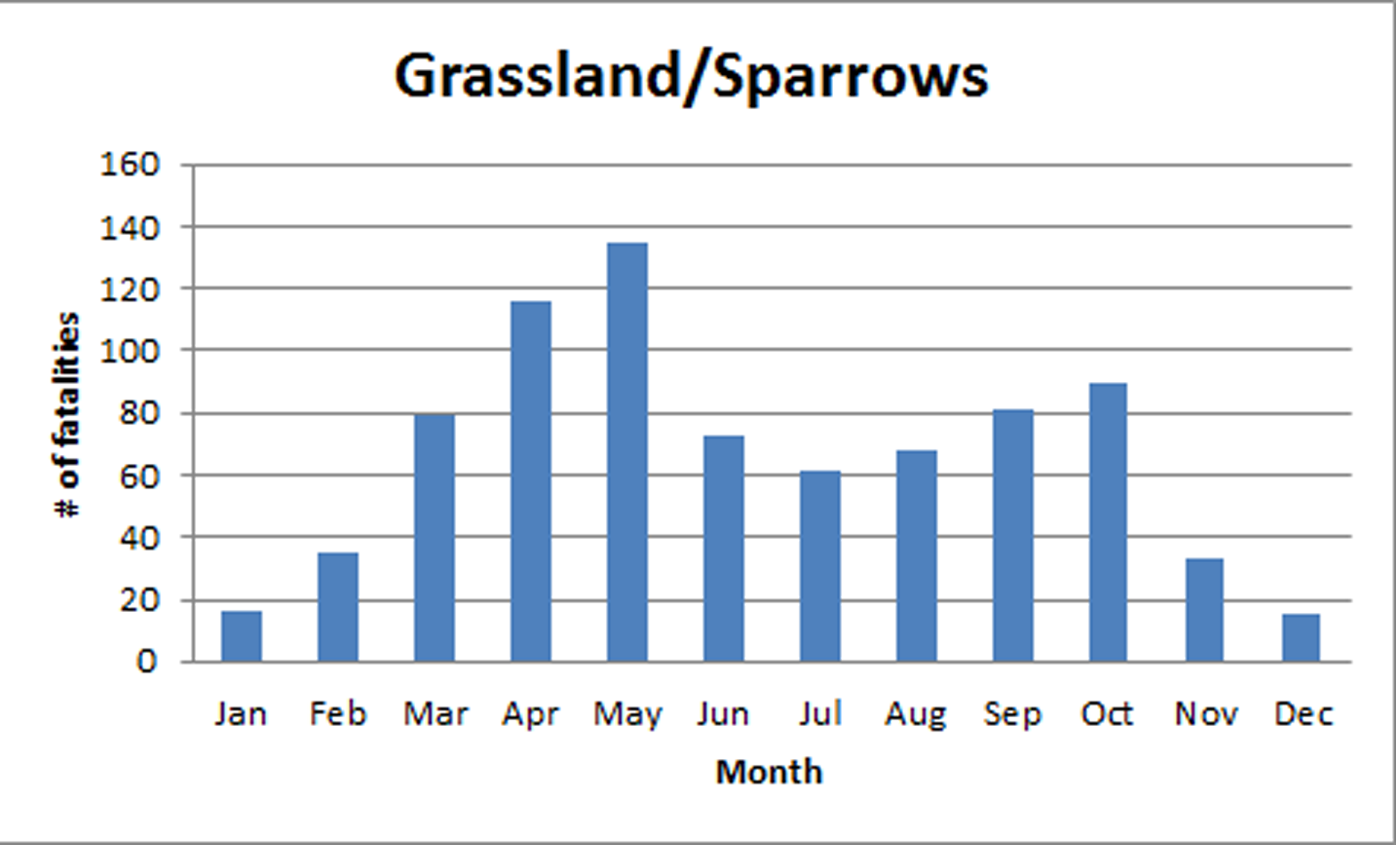

Supplement: Figure S8 — The monthly timing of fatalities for grassland species and sparrows (Alaudidae/Emberizidae) from 79 fatality studies for which the date when each fatality was found was provided. Timing of fatalities for small passerine families (presented alphabetically), unidentified passerines, and species of interest for 79 of 116 fatality monitoring studies in the United States and Canada for which dates were provided. (TIF) [file pone.0107491.s008.tif]

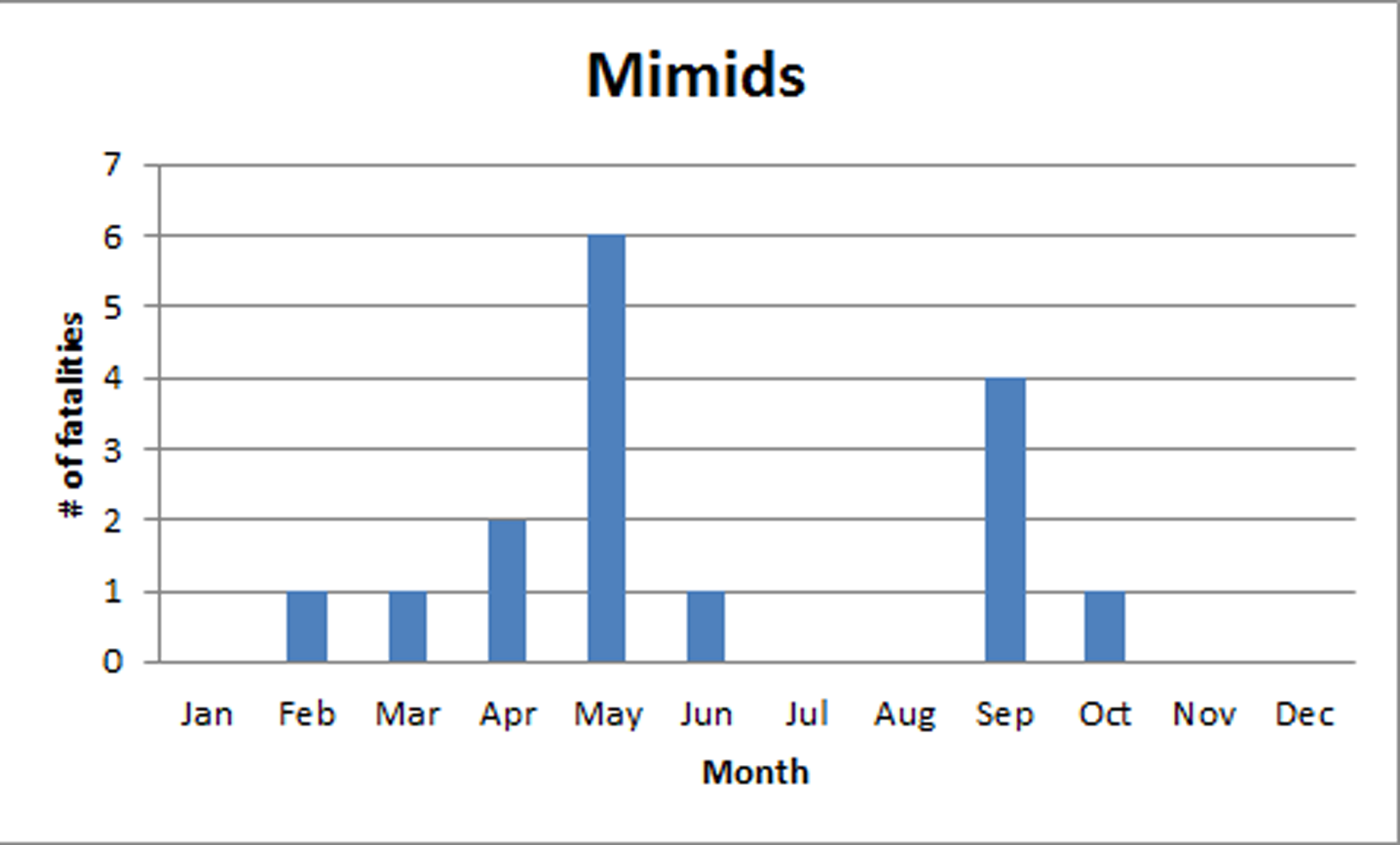

Supplement: Figure S9 — The monthly timing of fatalities for mimids (Mimidae) from 79 fatality studies for which the date when each fatality was found was provided. Timing of fatalities for small passerine families (presented alphabetically), unidentified passerines, and species of interest for 79 of 116 fatality monitoring studies in the United States and Canada for which dates were provided. (TIF) [file pone.0107491.s009.tif]

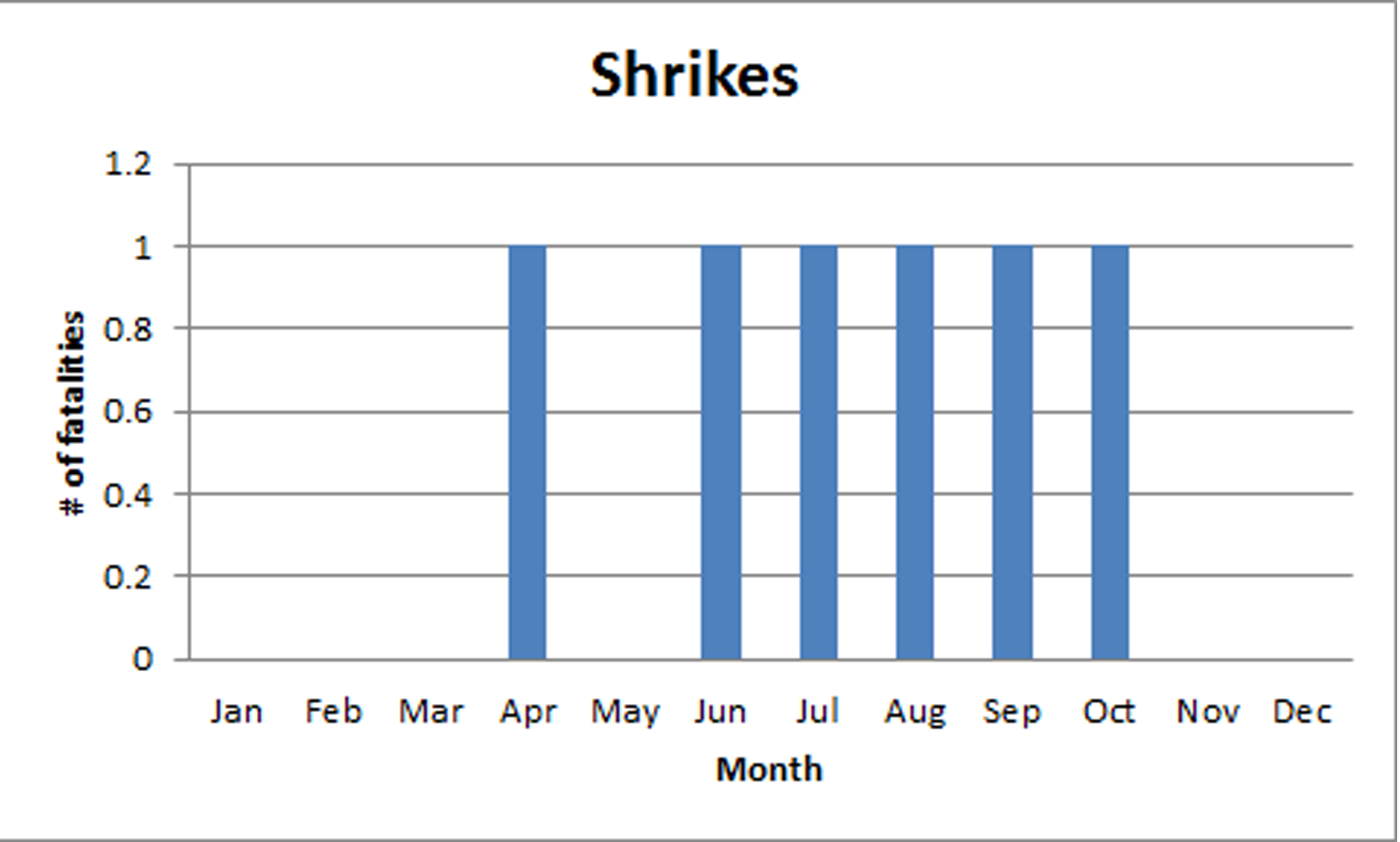

Supplement: Figure S10 — The monthly timing of fatalities for shrikes (Laniidae) from 79 fatality studies for which the date when each fatality was found was provided. Timing of fatalities for small passerine families (presented alphabetically), unidentified passerines, and species of interest for 79 of 116 fatality monitoring studies in the United States and Canada for which dates were provided. (TIF) [file pone.0107491.s010.tif]

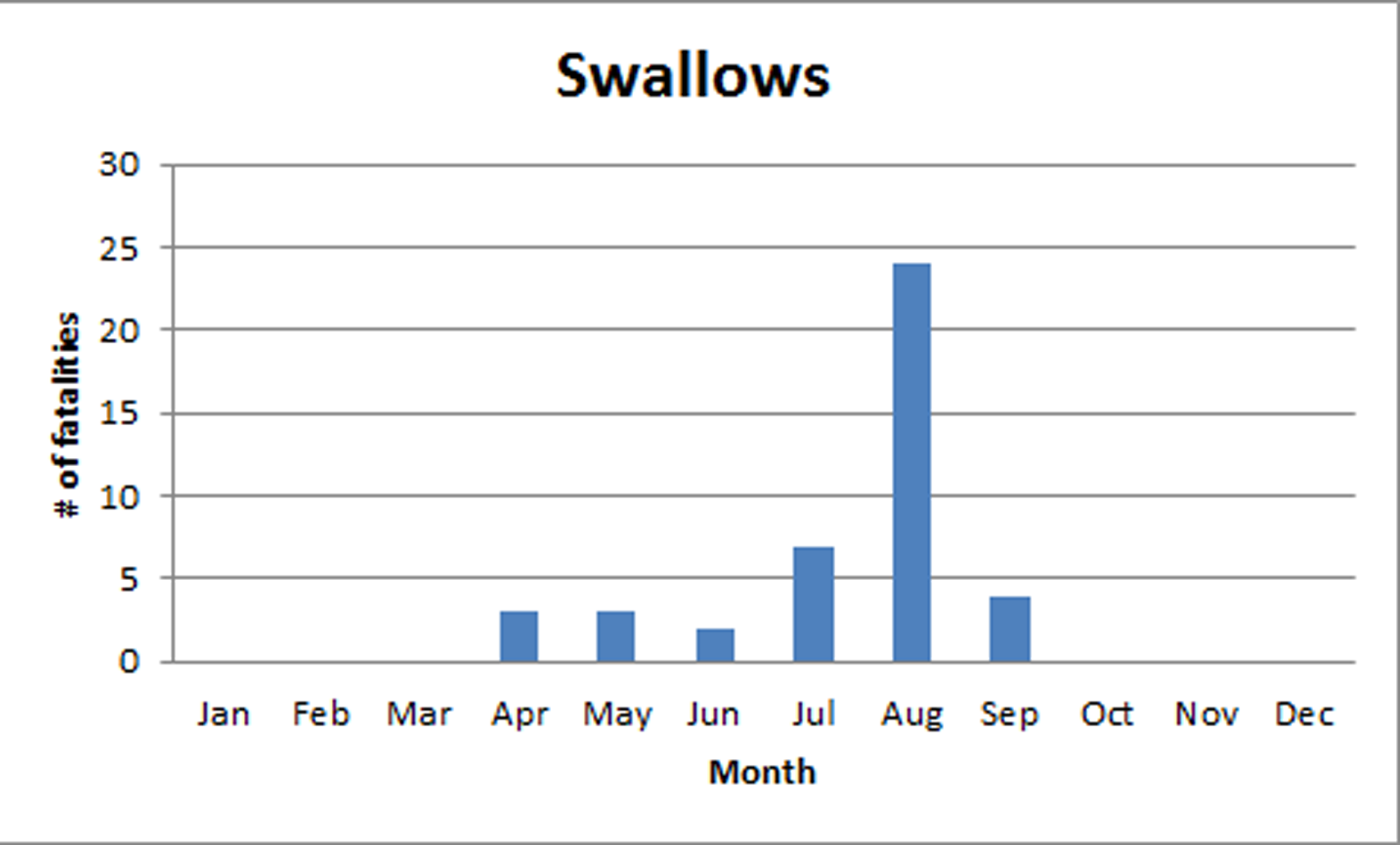

Supplement: Figure S11 — The monthly timing of fatalities for swallows (Hirundinidae) from 79 fatality studies for which the date when each fatality was found was provided. Timing of fatalities for small passerine families (presented alphabetically), unidentified passerines, and species of interest for 79 of 116 fatality monitoring studies in the United States and Canada for which dates were provided. (TIF) [file pone.0107491.s011.tif]

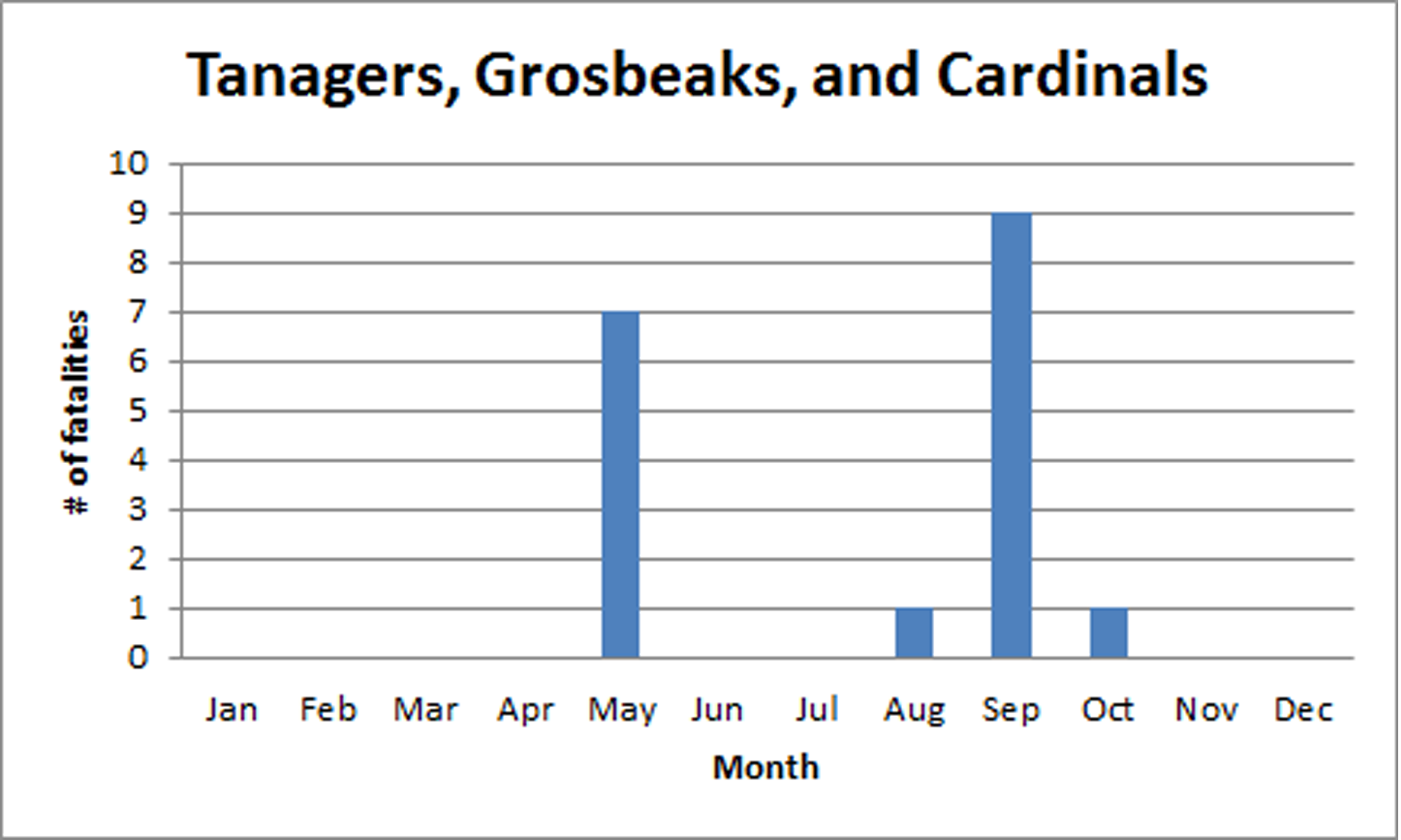

Supplement: Figure S12 — The monthly timing of fatalities for tanagers, grosbeaks, and cardinals (Cardinalidae), from 79 fatality studies for which the date when each fatality was found was provided. Timing of fatalities for small passerine families (presented alphabetically), unidentified passerines, and species of interest for 79 of 116 fatality monitoring studies in the United States and Canada for which dates were provided. (TIF) [file pone.0107491.s012.tif]

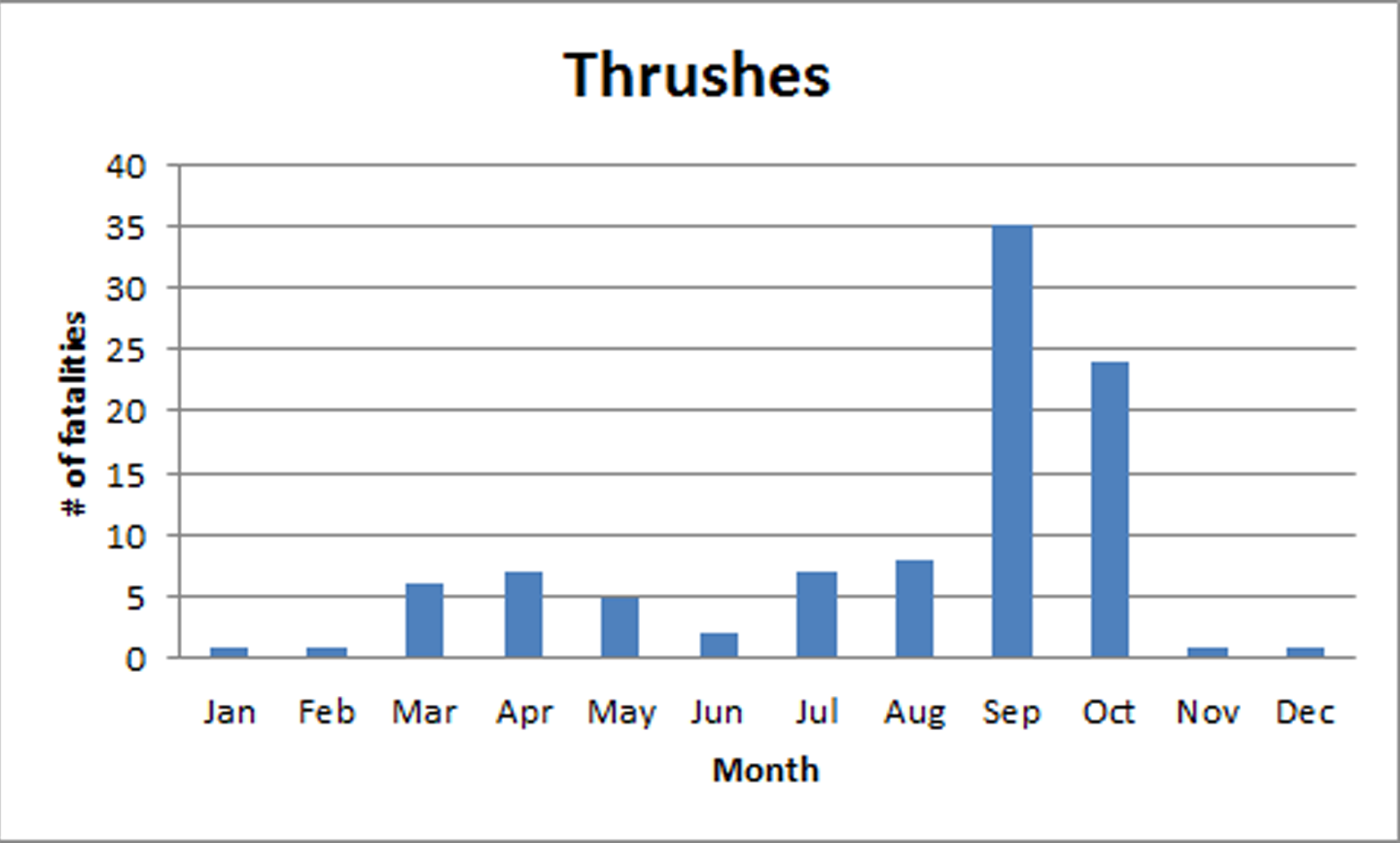

Supplement: Figure S13 — The monthly timing of fatalities for thrushes (Turdidae) from 79 fatality studies for which the date when each fatality was found was provided. Timing of fatalities for small passerine families (presented alphabetically), unidentified passerines, and species of interest for 79 of 116 fatality monitoring studies in the United States and Canada for which dates were provided. (TIF) [file pone.0107491.s013.tif]

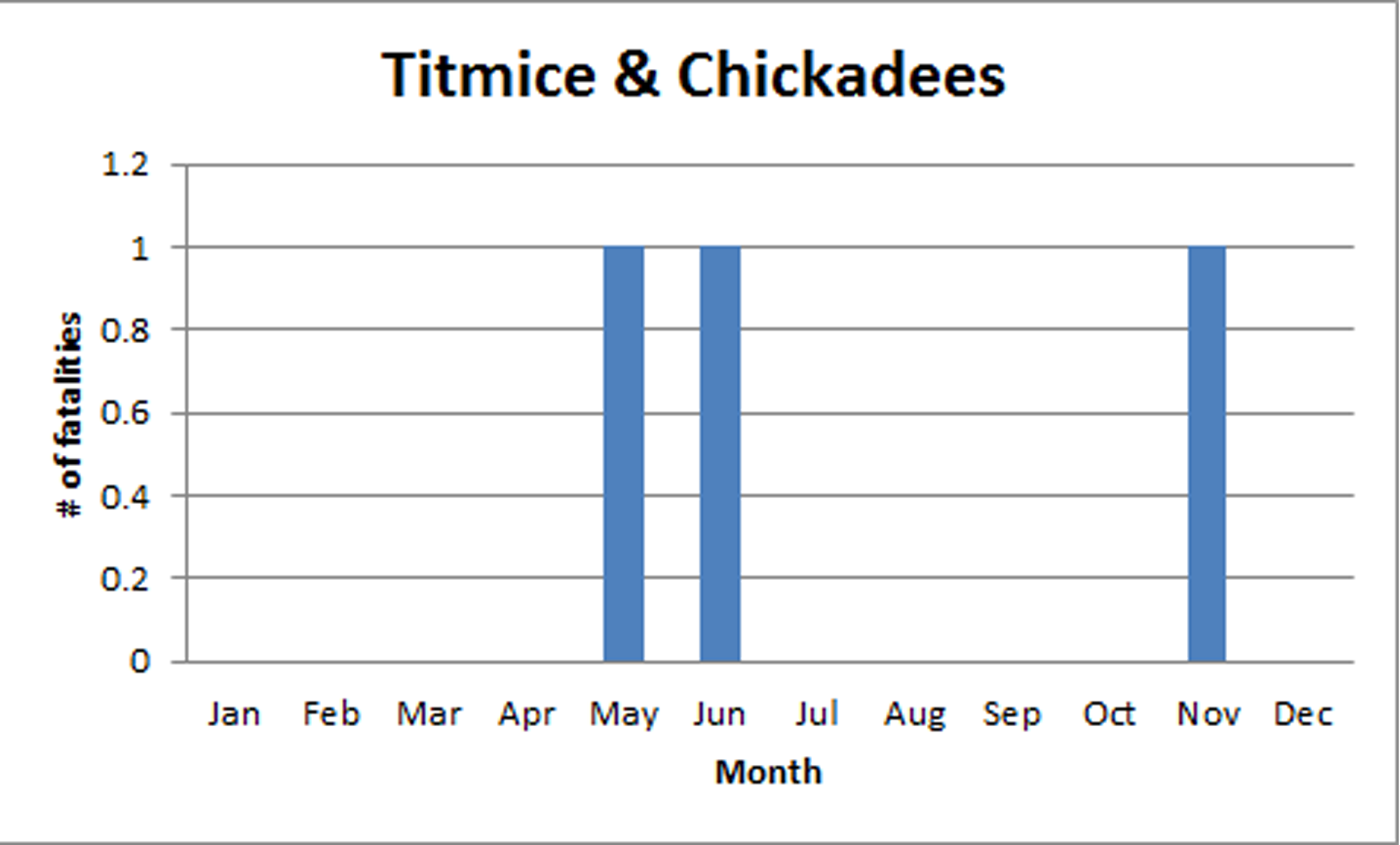

Supplement: Figure S14 — The monthly timing of fatalities for titmice and chickadees (Paridae) from 79 fatality studies for which the date when each fatality was found was provided. Timing of fatalities for small passerine families (presented alphabetically), unidentified passerines, and species of interest for 79 of 116 fatality monitoring studies in the United States and Canada for which dates were provided. (TIF) [file pone.0107491.s014.tif]

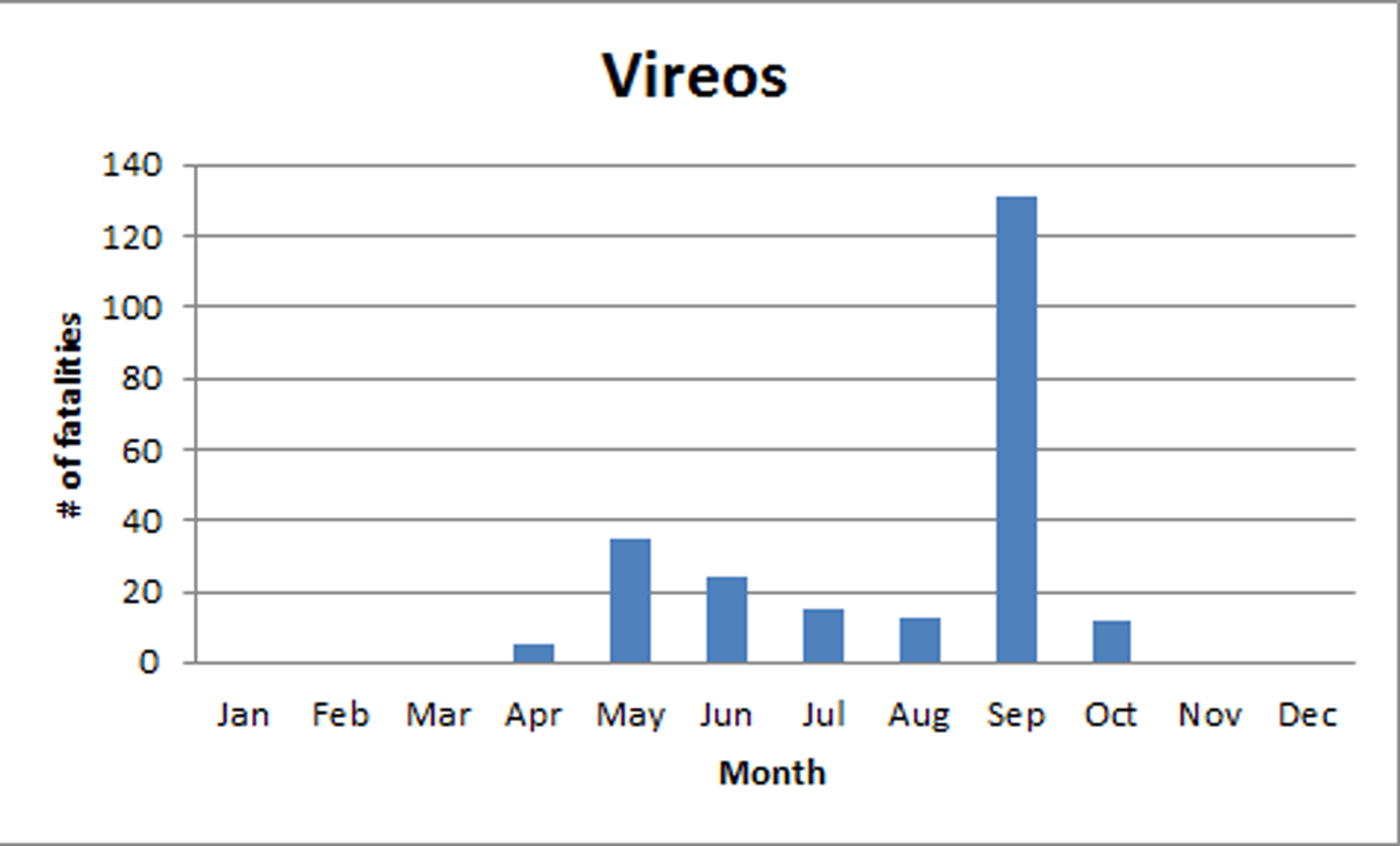

Supplement: Figure S15 — The monthly timing of fatalities for vireos (Vireonidae) from 79 fatality studies for which the date when each fatality was found was provided. Timing of fatalities for small passerine families (presented alphabetically), unidentified passerines, and species of interest for 79 of 116 fatality monitoring studies in the United States and Canada for which dates were provided. (TIF) [file pone.0107491.s015.tif]

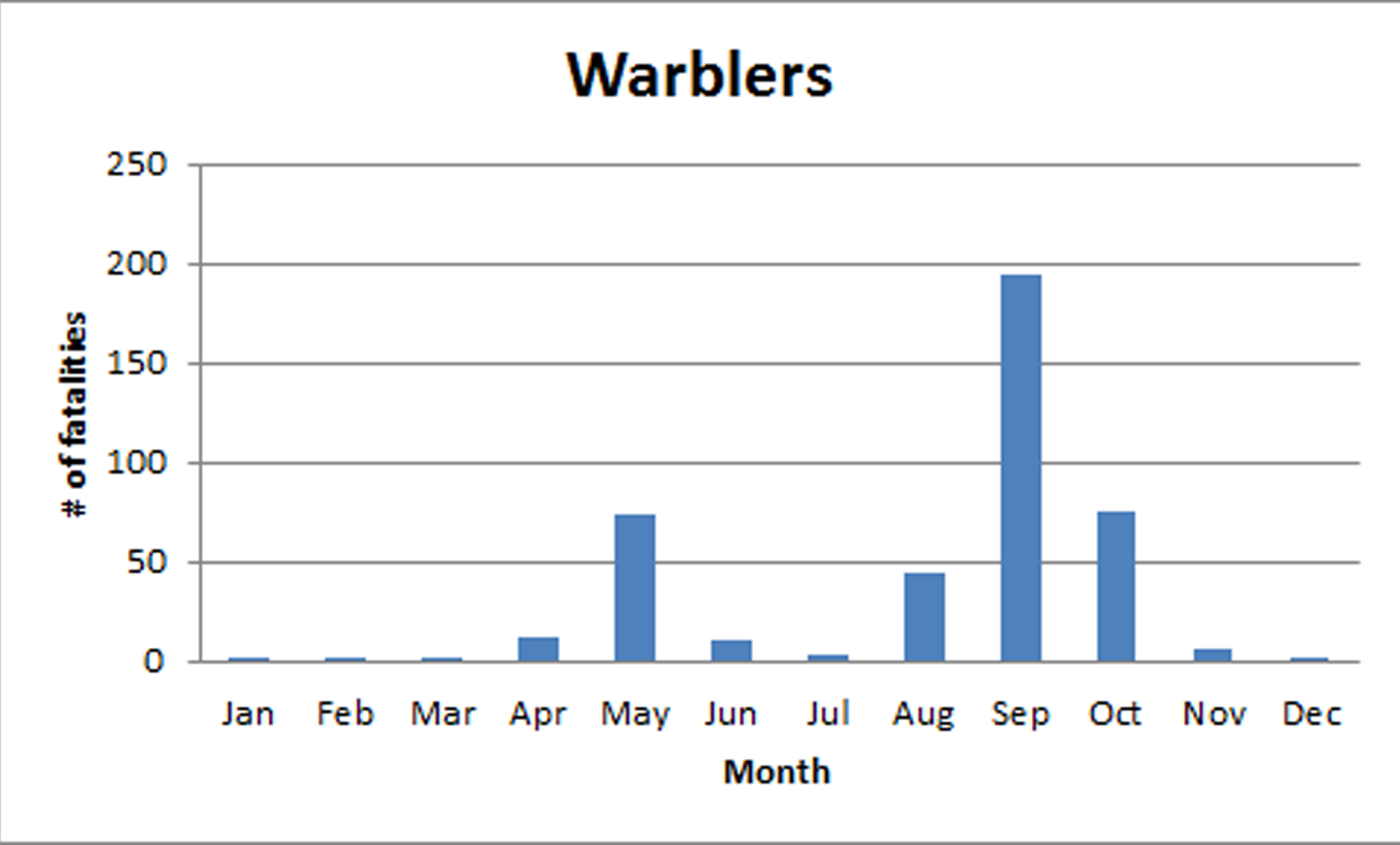

Supplement: Figure S16 — The monthly timing of fatalities for warblers (Parulidae) from 79 fatality studies for which the date when each fatality was found was provided. Timing of fatalities for small passerine families (presented alphabetically), unidentified passerines, and species of interest for 79 of 116 fatality monitoring studies in the United States and Canada for which dates were provided. (TIF) [file pone.0107491.s016.tif]

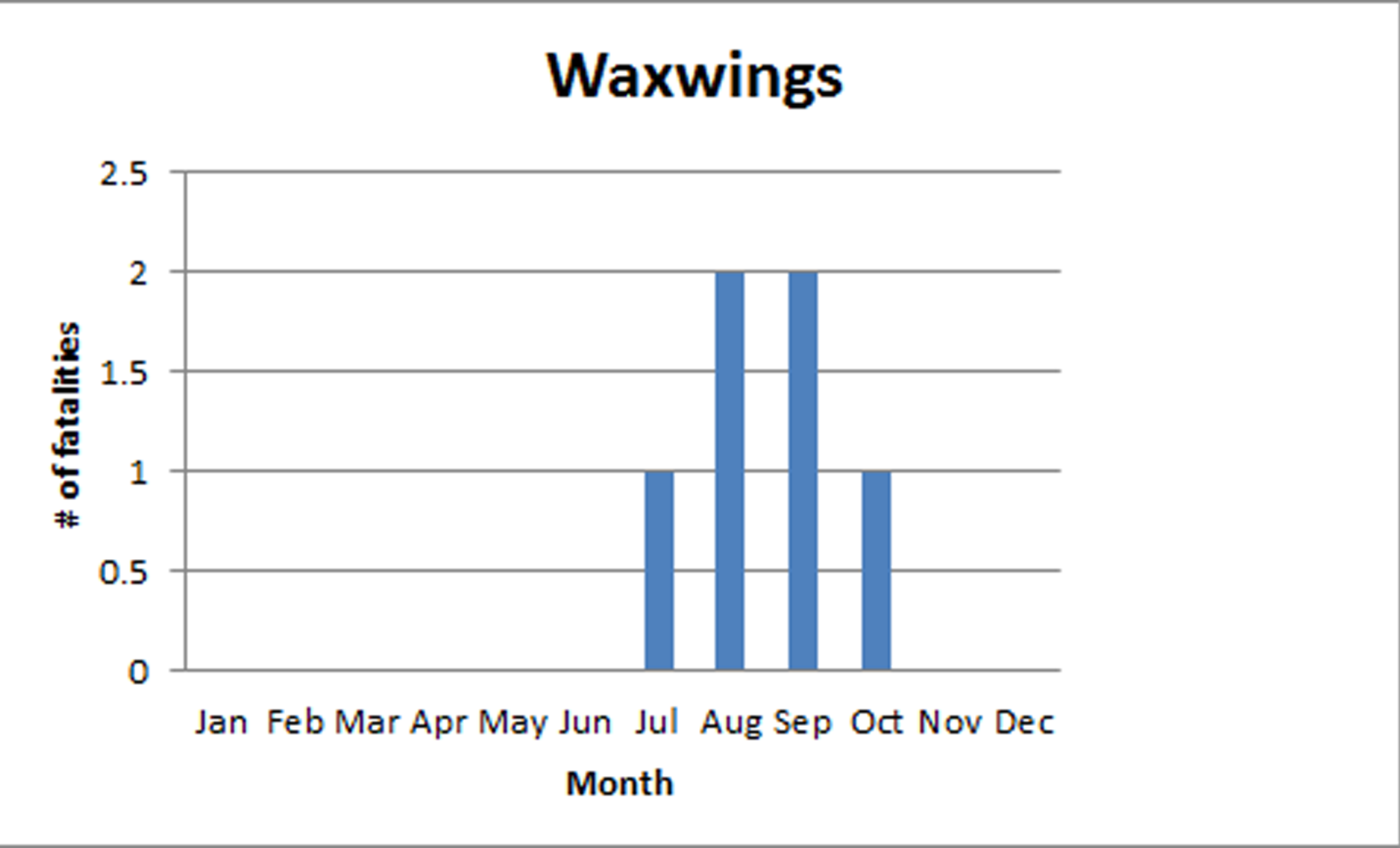

Supplement: Figure S17 — The monthly timing of fatalities for waxwings (Bombycillidae) from 79 fatality studies for which the date when each fatality was found was provided. Timing of fatalities for small passerine families (presented alphabetically), unidentified passerines, and species of interest for 79 of 116 fatality monitoring studies in the United States and Canada for which dates were provided. (TIF) [file pone.0107491.s017.tif]

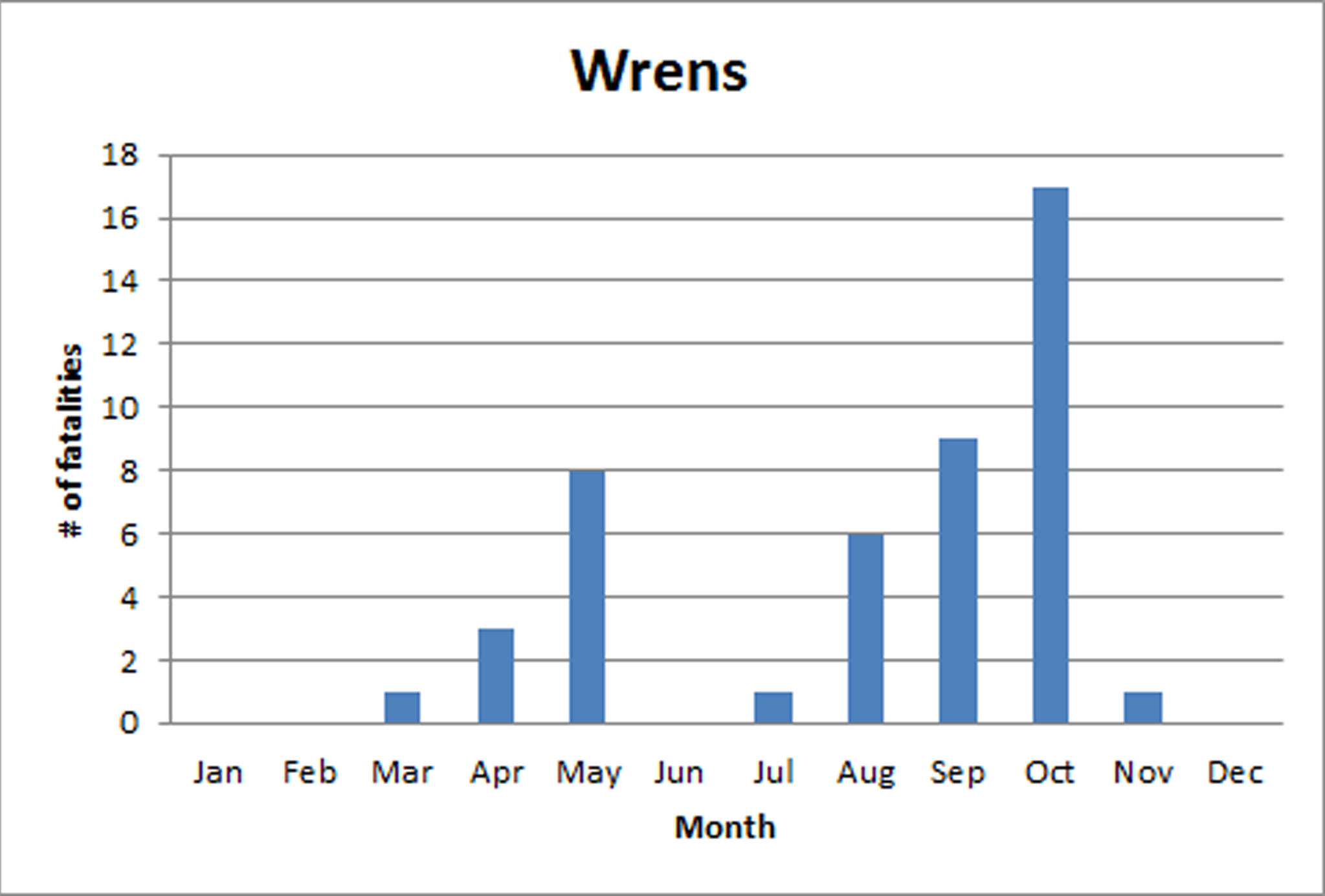

Supplement: Figure S18 — The monthly timing of fatalities for wrens (Troglodytidae) from 79 fatality studies from which the date when each fatality was found was provided. Timing of fatalities for small passerine families (presented alphabetically), unidentified passerines, and species of interest for 79 of 116 fatality monitoring studies in the United States and Canada for which dates were provided. (TIF) [file pone.0107491.s018.tif]

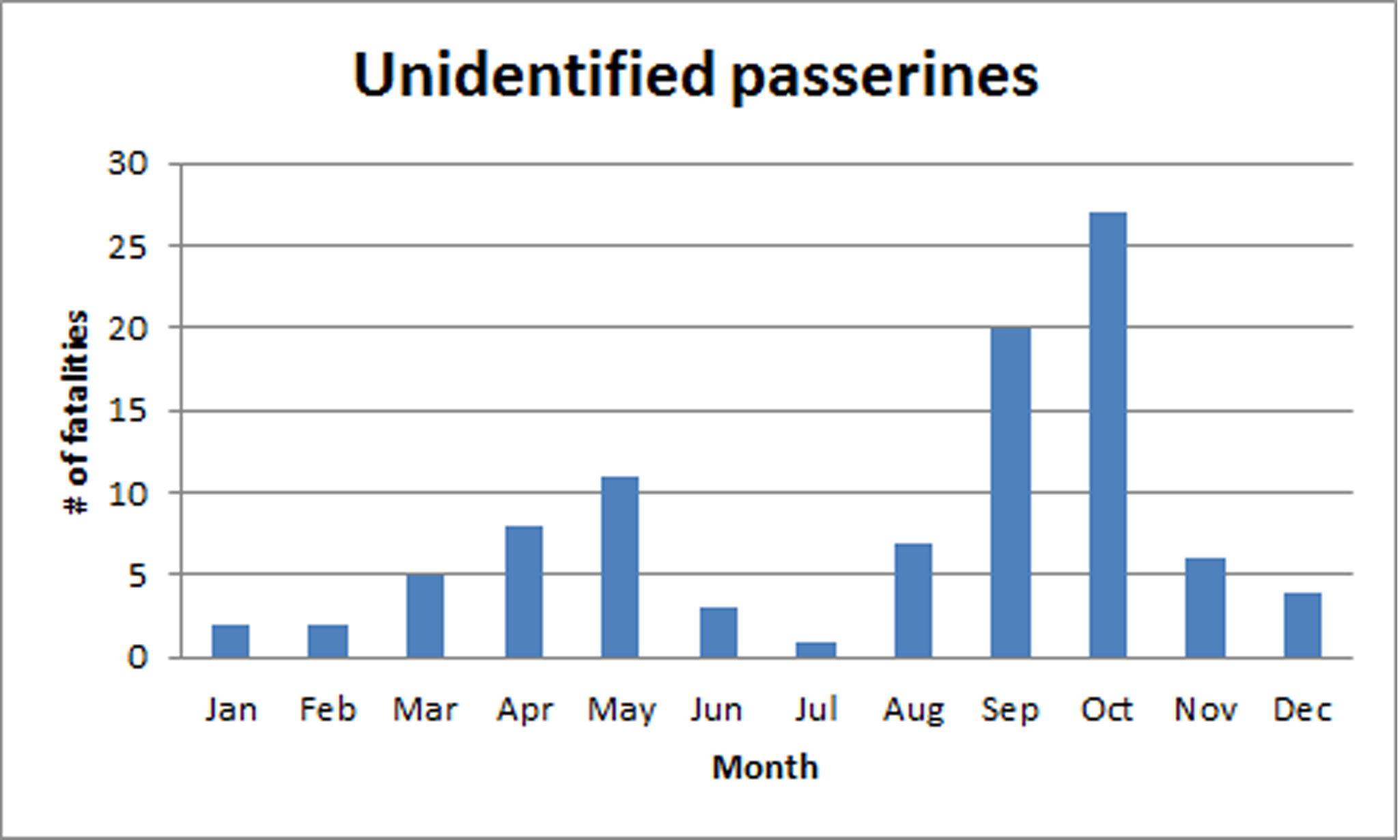

Supplement: Figure S19 — The monthly timing of fatalities for all unidentified passerines from 79 fatality studies for which the date when each fatality was found was provided. Timing of fatalities for small passerine families (presented alphabetically), unidentified passerines, and species of interest for 79 of 116 fatality monitoring studies in the United States and Canada for which dates were provided. (TIF) [file pone.0107491.s019.tif]

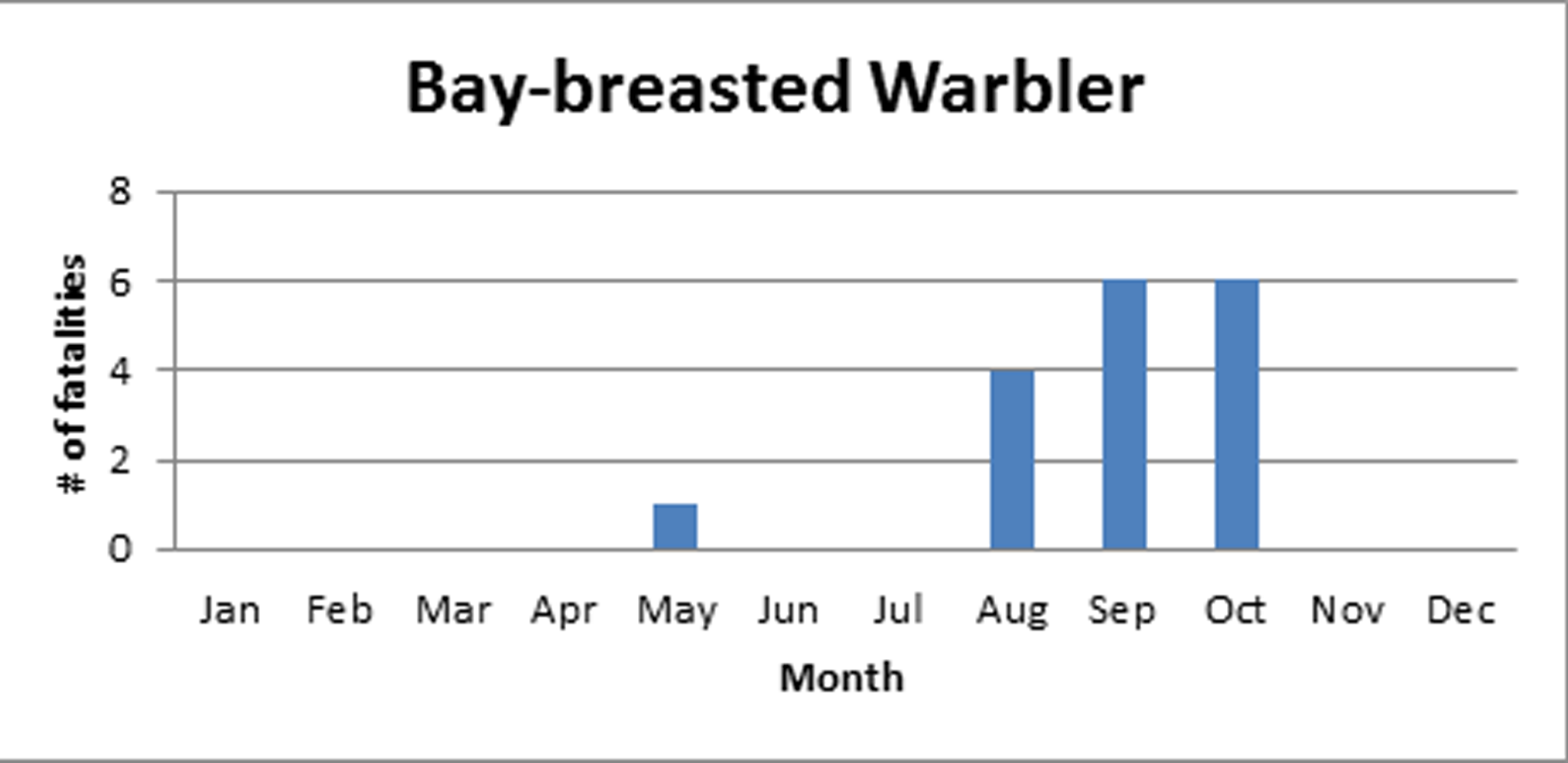

Supplement: Figure S20 — The monthly timing of fatalities for the bay-breasted warbler ( Setophaga castanea ) from 79 fatality studies for which the date when each fatality was found was provided. Timing of fatalities for small passerine families (presented alphabetically), unidentified passerines, and species of interest for 79 of 116 fatality monitoring studies in the United States and Canada for which dates were provided. (TIF) [file pone.0107491.s020.tif]

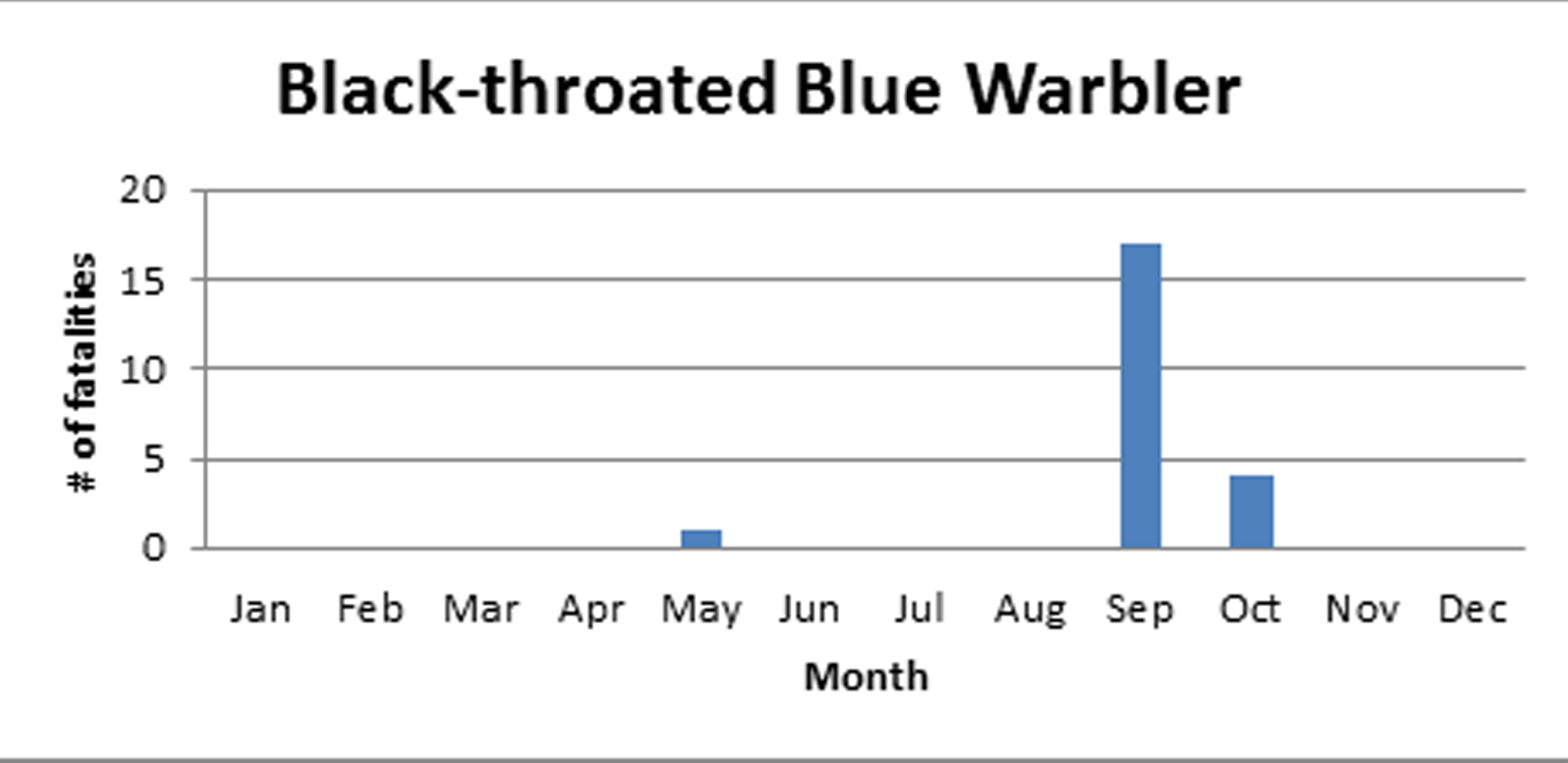

Supplement: Figure S21 — The monthly timing of fatalities for the black-throated blue warbler ( Setophaga caerulescens ) from 79 fatality studies for which the date when each fatality was found was provided. Timing of fatalities for small passerine families (presented alphabetically), unidentified passerines, and species of interest for 79 of 116 fatality monitoring studies in the United States and Canada for which dates were provided. (TIF) [file pone.0107491.s021.tif]

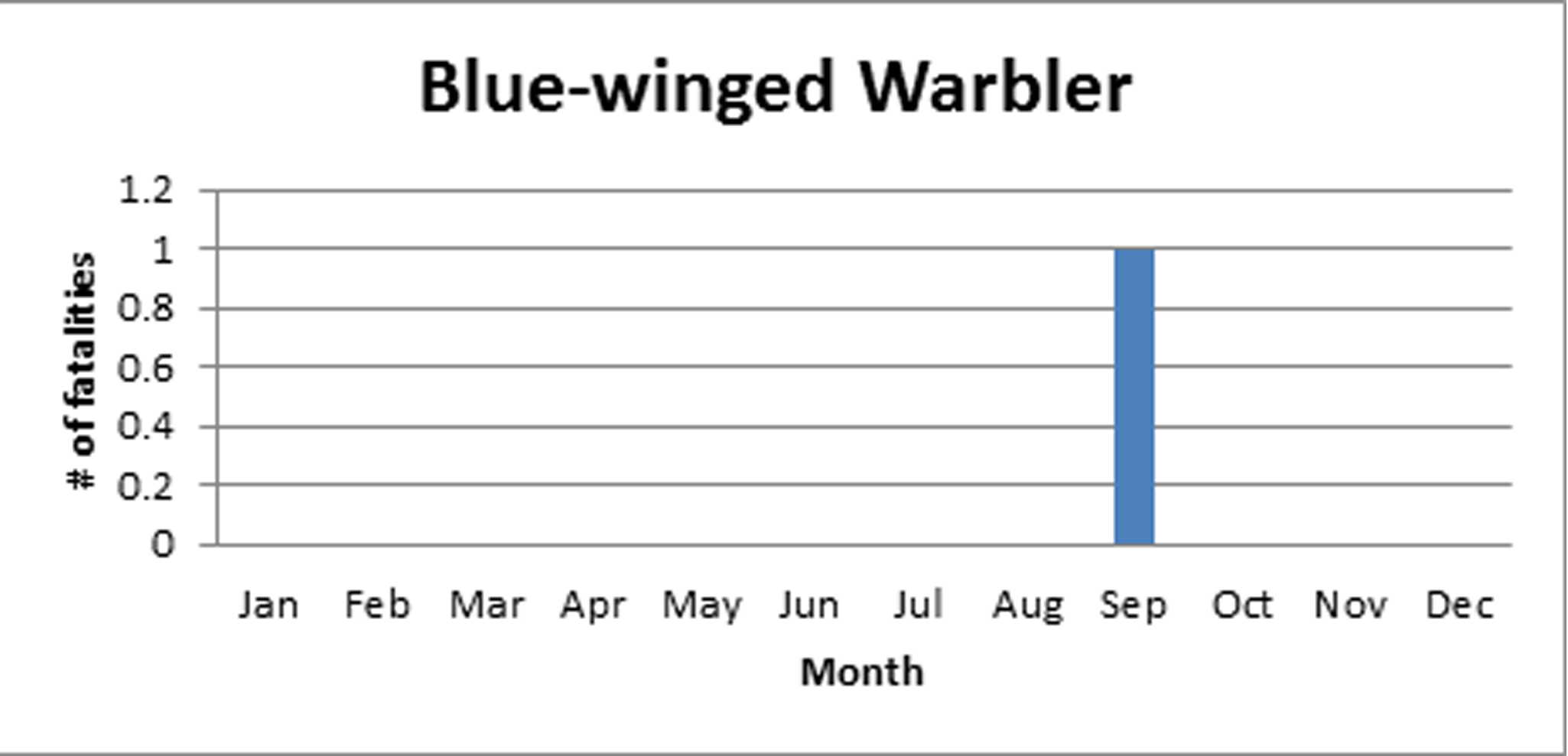

Supplement: Figure S22 — The monthly timing of fatalities for the blue-winged warbler ( Vermivora cyanoptera ) from 79 fatality studies for which the date when each fatality was found was provided. Timing of fatalities for small passerine families (presented alphabetically), unidentified passerines, and species of interest for 79 of 116 fatality monitoring studies in the United States and Canada for which dates were provided. (TIF) [file pone.0107491.s022.tif]

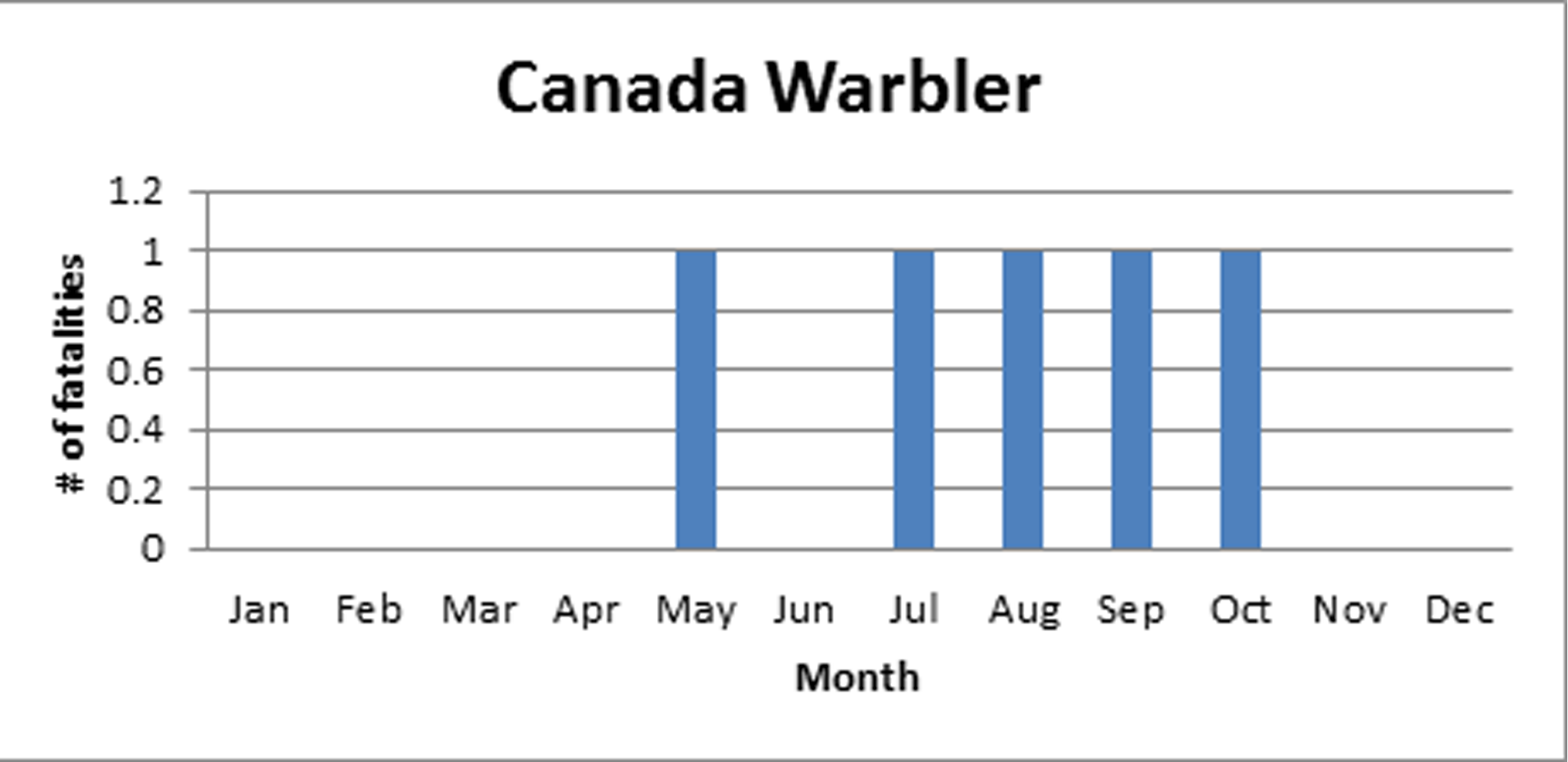

Supplement: Figure S23 — The monthly timing of fatalities for the Canada warbler ( Cardellina canadensis ) from 79 fatality studies for which the date when each fatality was found was provided. Timing of fatalities for small passerine families (presented alphabetically), unidentified passerines, and species of interest for 79 of 116 fatality monitoring studies in the United States and Canada for which dates were provided. (TIF) [file pone.0107491.s023.tif]

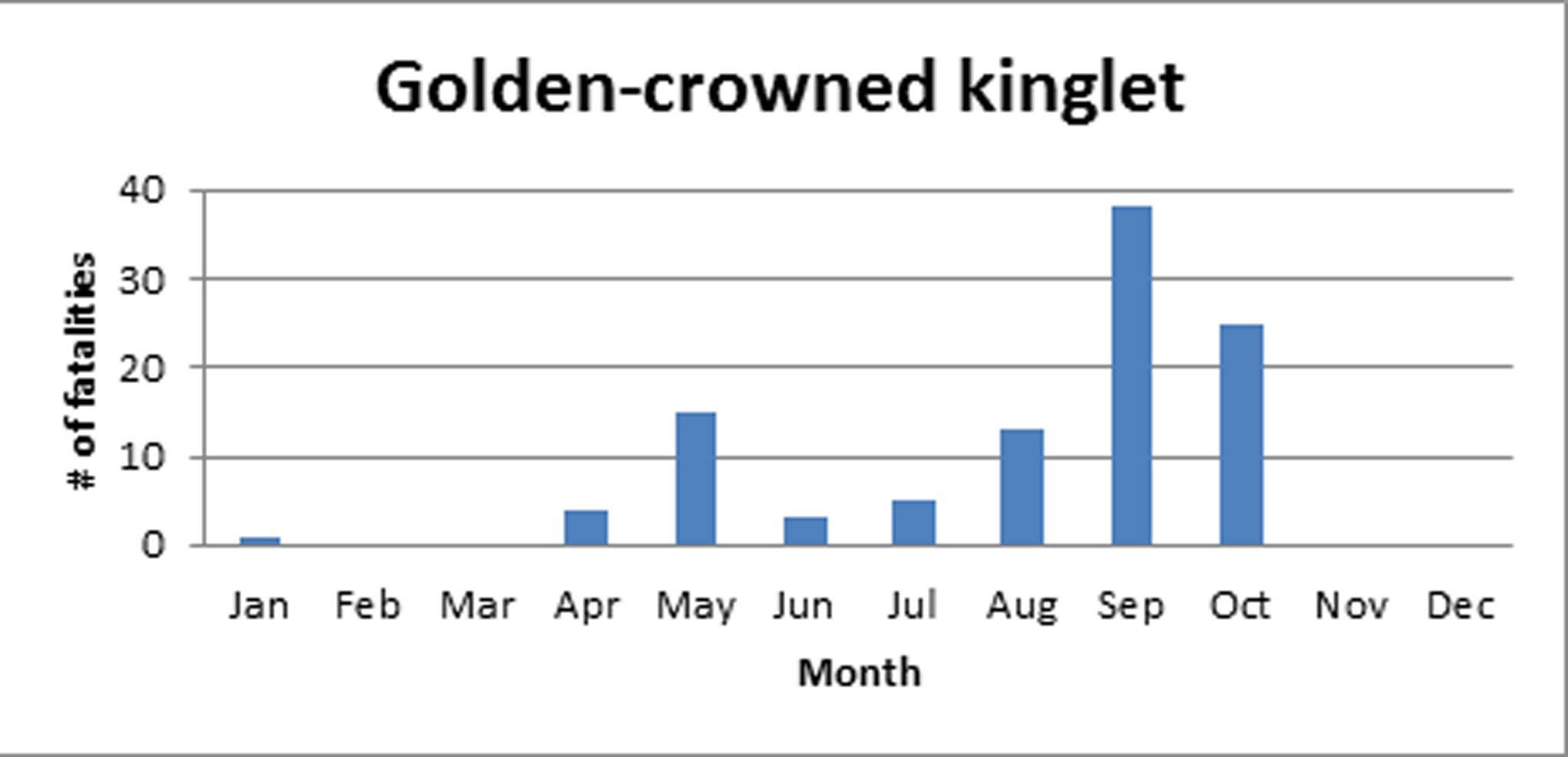

Supplement: Figure S24 — The monthly timing of fatalities for the golden-crowned kinglet ( Regulus satrapa ) from 79 fatality studies for which the date when each fatality was found was provided. Timing of fatalities for small passerine families (presented alphabetically), unidentified passerines, and species of interest for 79 of 116 fatality monitoring studies in the United States and Canada for which dates were provided. (TIF) [file pone.0107491.s024.tif]

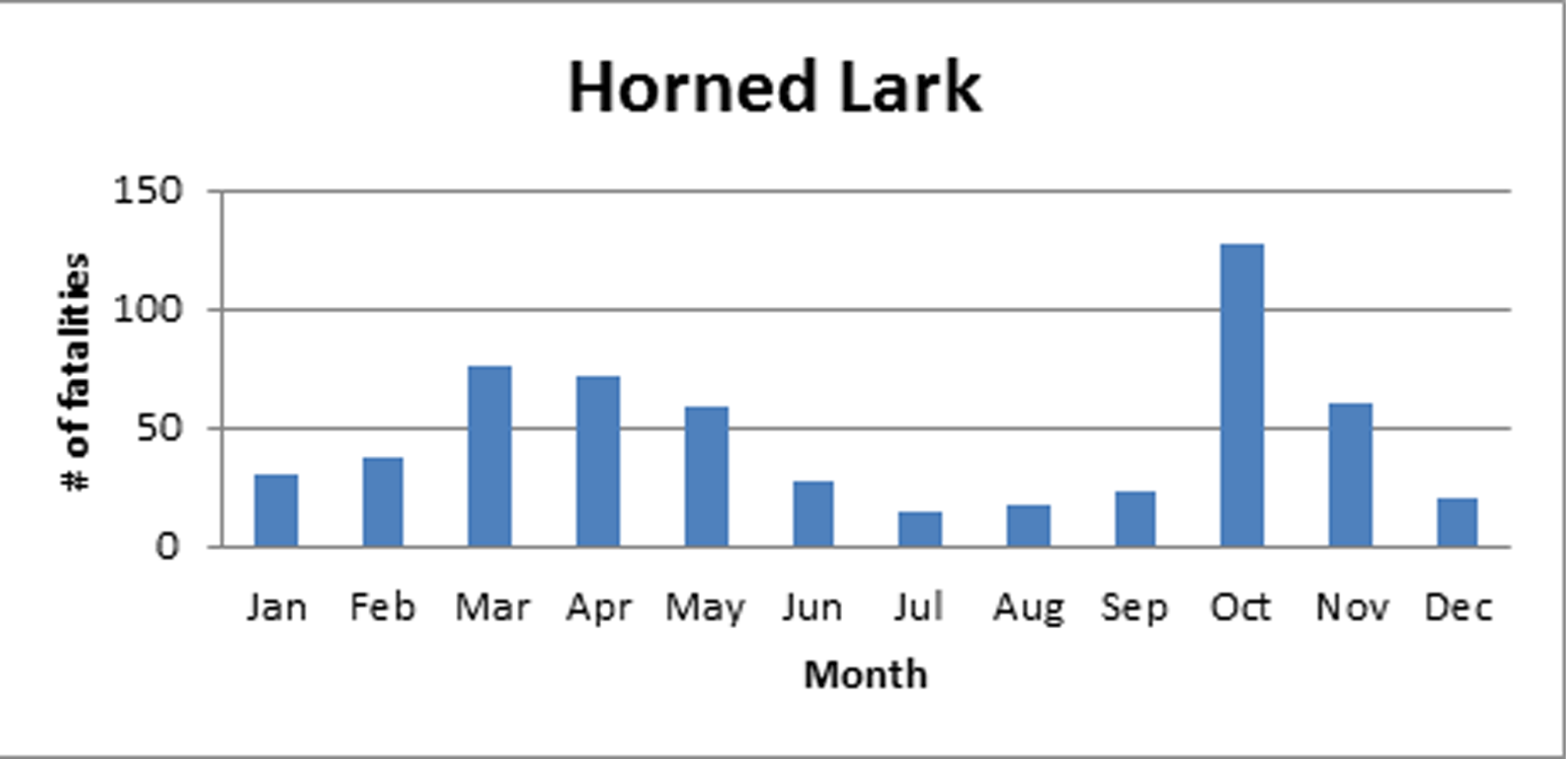

Supplement: Figure S25 — The monthly timing of fatalities for the horned lark ( Eremophila alpestris ) from 79 fatality studies for which the date when each fatality was found was provided. Timing of fatalities for small passerine families (presented alphabetically), unidentified passerines, and species of interest for 79 of 116 fatality monitoring studies in the United States and Canada for which dates were provided. (TIF) [file pone.0107491.s025.tif]

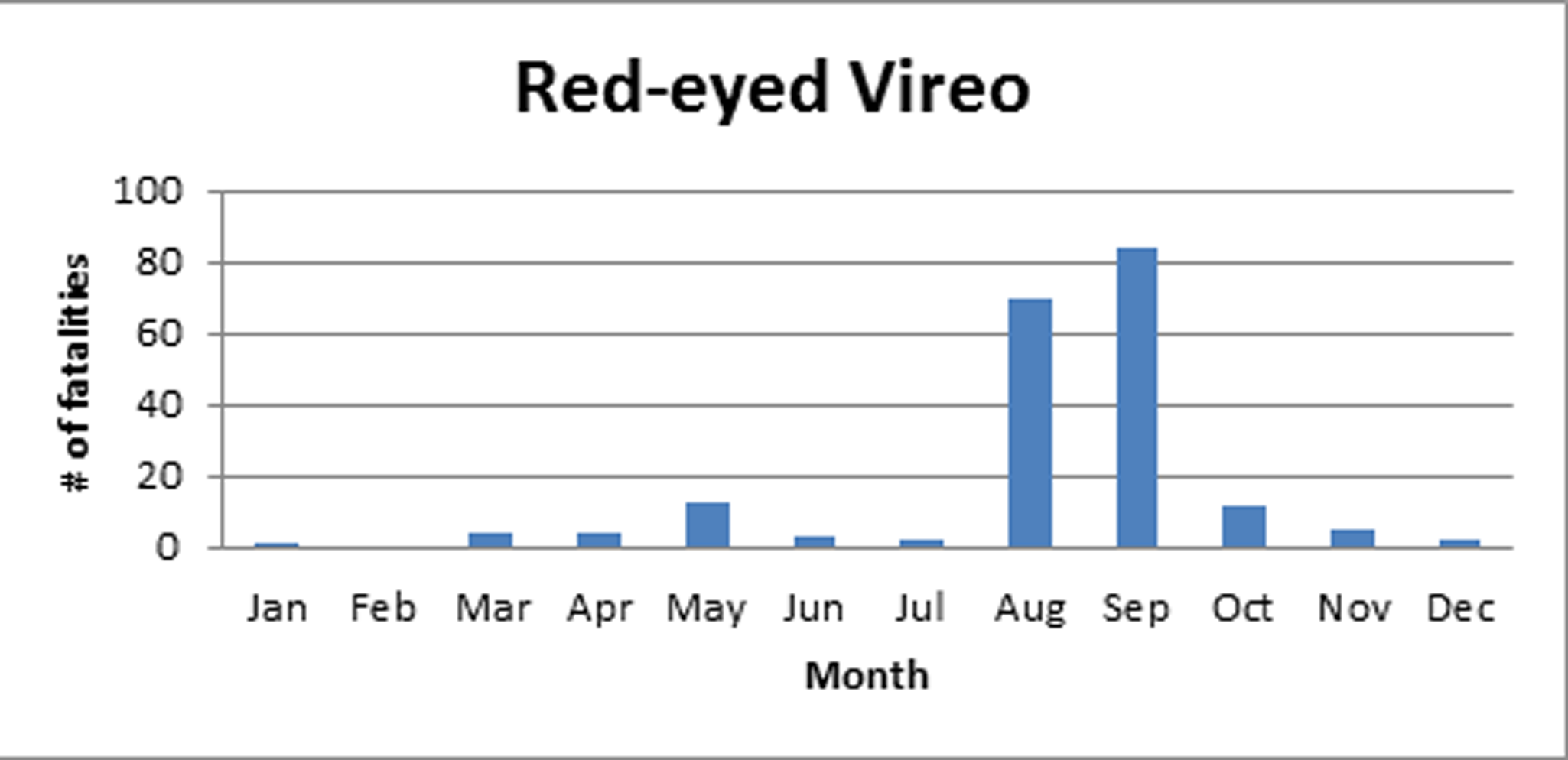

Supplement: Figure S26 — The monthly timing of fatalities for the red-eyed vireo ( Vireo olivaceus ) from 79 fatality studies for which the date when each fatality was found was provided. Timing of fatalities for small passerine families (presented alphabetically), unidentified passerines, and species of interest for 79 of 116 fatality monitoring studies in the United States and Canada for which dates were provided. (TIF) [file pone.0107491.s026.tif]

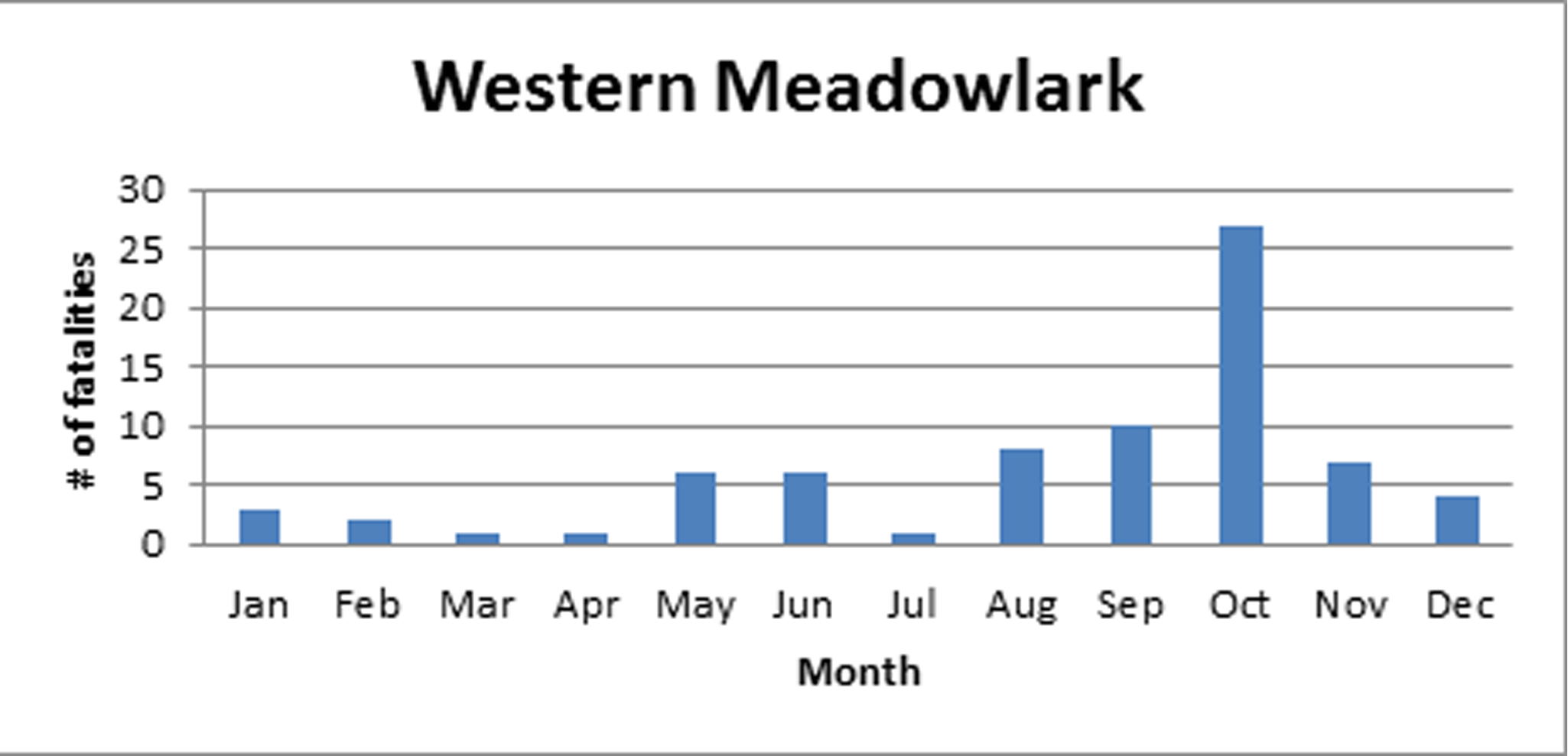

Supplement: Figure S27 — The monthly timing of fatalities for the western meadowlark ( Sturnella neglecta ) from 79 fatality studies for which the date when each fatality was found was provided. Timing of fatalities for small passerine families (presented alphabetically), unidentified passerines, and species of interest for 79 of 116 fatality monitoring studies in the United States and Canada for which dates were provided. (TIF) [file pone.0107491.s027.tif]
