# Supplementary material for: A Comprehensive Analysis of Small-Passerine Fatalities from Collision with Turbines at Wind Energy Facilities
Source: PLoS One. 2014 Sep 15;9(9):e107491. doi: 10.1371/journal.pone.0107491 (PMC4164633; doi:10.1371/journal.pone.0107491)
Supplement: Appendix S1 — Description of fatality studies conducted at wind energy facilities, including project location, number of turbines, size (megawatts [MW]), and height of turbines; and post-construction study information, divided into avifaunal biome regions for the United States and Canada. Blank spaces indicate that data was not available. (DOCX) [file pone.0107491.s028.docx]

**Appendix S1. Description of fatality studies conducted at wind energy facilities, including project location, number of turbines, size (megawatts [MW]), and height of turbines; and post-construction study information, divided into avifaunal biome regions for the United States and Canada.** Blank spaces indicate that data was not available.

| **Project Name (phase; year)** | **County, State/Prov.** | **# Turbines in WRA** | **# MW** | **Total # Turbines Searches** | **Tower Height (m)** | **Seasons Sampled** | **Search Interval** | **Plot Size** | **Study Year** | **References** |
| --- | --- | --- | --- | --- | --- | --- | --- | --- | --- | --- |
| **Projects with Small Bird Estimates by Avifaunal Biome Region** | | | | | | | | | | |
| **Eastern Biome** | | | | | | | | | | |
| Criterion (2011) | Garrett, MD | 28 | 70 | 5,316 | 80 | 7.5 months | Daily | 40-50 m radius | 2011 | [1] |
| Mount Storm (2009) | Grant, WV | 132 | 264 | 2,405 | 78 | 6 months | Weekly (28 turbines), daily (16 turbines) | 20 to 60 m from turbine | 2009 | [2, 3] |
| Mount Storm (2010) | Grant, WV | 132 | 264 | 2,105 | 78 | 6 months | Daily | 20 to 60 m from turbine | 2010 | [4, 5] |
| Mount Storm (2011) | Grant, WV | 132 | 264 | 3,794 | 78 | 6 months | Daily | 20 to 60 m from turbine | 2011 | [6, 7] |
| Noble Bliss (2008) | Wyoming, NY | 67 | 100 | 1,954 | 80 | Spring, summer, fall | Daily (8 turbines), 3-day (8 turbines), weekly ( 7 turbines) | 120 m x 120 m | 2008 | [8] |
| Noble Bliss (2009) | Wyoming, NY | 67 | 100 | 1,717 | 80 | Spring, summer, fall | Weekly, 8 turbines searched daily from July 1 to August 15 | 120 m x 120 m | 2009 | [9] |
| Noble Wethersfield (2010) | Wyoming, NY | 84 | 126 | 698 | 80 | Spring, summer, fall | Weekly | 120 m x 120 m | 2010 | [10] |
| **Intermountain West Biome** | | | | | | | | | | |
| Biglow Canyon (Phase I; 2008) | Sherman, OR | 76 | 125.4 | 850 | 80 | 1 year | Bi-monthly (spring, fall), monthly (winter, summer) | 110 m x 110 m | 2008 | [11] |
| Biglow Canyon (Phase I; 2009) | Sherman, OR | 76 | 125.4 | 850 | 80 | 1 year | Bi-monthly (spring, fall), monthly (winter, summer) | 110 m x 110 m | 2009 | [12] |
| Biglow Canyon (Phase II; 2009/2010) | Sherman, OR | 65 | 150 | 850 | 80 | 1 year | Bi-monthly (spring, fall), monthly (winter, summer) | 250 m x 250 m | 2009/ 2010 | [13] |
| Biglow Canyon (Phase II; 2010/2011) | Sherman, OR | 65 | 150 | 799 | 80 | 1 year | Bi-weekly(spring, fall), monthly (summer, winter) | 252 m x 252 m | 2010/ 2011 | [14] |
| Biglow Canyon (Phase III, 2010/2011) | Sherman, OR | 76 | 174.8 | 800 | 80 | 1 year | Bi-weekly(spring, fall), monthly (summer, winter) | 252 m x 252 m | 2010/ 2011 | [15] |
| Combine Hills (2004) | Umatilla, OR | 41 | 41 | 574 | 53 | 1 year | Monthly | 90-m radius | 2004/ 2005 | [16] |
| Combine Hills (2011) | Umatilla, OR | 104 | 104 | 955 | 53 | 1 year | Bi-weekly(spring, fall), monthly (summer, winter) | 180 m x 180 m | 2011 | [17] |
| Dry Lake I | Navajo, AZ | 30 | 63 | 768 | 78 | 1 year | Bi-monthly (spring, fall), monthly (winter, summer) | 160 m x 160 m | 2009/ 2010 | [18] |
| Dry Lake II | Navajo, AZ | 31 | 65 |  | 78 | 1 year | Twice weekly (spring, summer, fall), weekly (winter) | 160 m x 160 m | 2011-2012 | [19] |
| Elkhorn (2008) | Union, OR | 61 | 101 | 732 | 80 | 1 year | Monthly | 220 m x 220 m | 2008 | [20] |
| Elkhorn (2010) | Union, OR | 61 | 101 | 619 | 80 | 1 year | Bi-monthly (spring, fall), monthly (winter, summer) | 220 m x 220 m | 2010 | [21] |
| Foote Creek Rim (Phase I; 1999) | Carbon, WY | 69 | 41.4 | 42 | 40 | 1 year | Monthly | 126 m x 126 m | 1998-2002 | [22] |
| Foote Creek Rim (Phase I; 2000) | Carbon, WY | 69 | 41.4 | 42 | 40 | 1 year | Monthly | 126 m x 126 m | 1998-2002 | [22] |
| Foote Creek Rim (Phase I; 2001-2002) | Carbon, WY | 69 | 41.4 | 42 | 40 | 1 year | Monthly | 126 m x 126 m | 1998-2002 | [22] |
| Goodnoe | Klickitat, WA | 47 | 94 | 455 | 80 | 1 year | 14 days during migration periods, 28 days during non-migration periods | 180 m x 180 m | 2009/ 2010 | [23] |
| Hay Canyon | Sherman, OR | 48 | 100.8 | 320 | 79 | 1 year | Bi-monthly (spring, fall), monthly (winter, summer) | 180 m x 180 m | 2009/ 2010 | [24] |
| Hopkins Ridge (2006) | Columbia, WA | 83 | 150 | 865 | 67 | 1 year | Monthly, weekly (subset of 22 turbines spring and fall migration) | 180 m x 180 m | 2006 | [25] |
| Hopkins Ridge (2008) | Columbia, WA | 87 | 156.6 | 713 | 67 | 1 year | Bi-monthly (spring, fall), monthly (winter, summer) | 180 m x 180 m | 2008 | [26] |
| Kittitas Valley (2011-2012) | Kittitas, WA | 48 | 100.8 | 815 | 80 | 1 year | Bi weekly from Aug 15 - Oct 31 and March 16 - May 15; every 4 weeks from Nov 1 - March 15 and May 16 - Aug 14 | 100 m x 102 m | 2011/ 2012 | [27] |
| Klondike | Sherman, OR | 16 | 24 | 208 | 80 | 1 year | Monthly | 140 m x 140 m | 2002/ 2003 | [28] |
| Klondike II | Sherman, OR | 50 | 75 | 500 | 80 | 1 year | Bi-monthly (spring, fall), monthly (summer, winter) | 180 m x 180 m | 2005/ 2006 | [29] |
| Klondike III (Phase I) | Sherman, OR | 125 | 223.6 | 1,426 | 80 | 2 year | Bi-monthly (spring, fall migration), monthly (summer, winter) | 240 m x 240 m (1.5MW) 252 m x 252 m (2.3MW) | 2007-2009 | [30] |
| Klondike IIIa (Phase II) | Sherman, OR | 51 | 76.5 | 1,088 | 80 | 2 years | Bi-monthly (spring, fall), monthly (summer, winter) | 240 m x 240 m | 2008-2010 | [31] |
| Linden Ranch | Klickitat, WA | 25 | 50 | 441 | 80 | 1 year | Bi-weekly(spring, fall), monthly (summer, winter) | 110 m x 110 m | 2010 | [32] |
| Marengo I (2009) | Columbia, WA | 78 | 140.4 |  | 67 | 1 year | Bi-monthly (spring, fall), monthly (winter, summer) | 180 m x 180 m | 2009/ 2010 | [33] |
| Marengo II (2009) | Columbia, WA | 39 | 70.2 | 360 | 67 | 1 year | Bi-monthly (spring, fall), monthly (winter, summer) | 180 m x 180 m | 2009/ 2010 | [34] |
| Nine Canyon | Benton, WA | 37 | 48.1 | 703 | 60 | 1 year | Bi-monthly (spring, summer, fall), monthly (winter) | 90-m radius | 2002/ 2003 | [35] |
| Stateline (2002) | Walla Walla, WA and Umatilla, OR | 454 | 300 | 668 | 50 | 1 year | Bi-weekly, monthly | Minimum 126 m x 126 m | 2002 | [36] |
| Stateline (2003) | Walla Walla, WA and Umatilla, OR | 454 | 300 | 668 | 50 | 1 year | Bi-weekly, monthly | Minimum 126 m x 126 m | 2003 | [36] |
| Stateline (2006) | Walla Walla, WA and Umatilla, OR | 454 | 300 | 668 | 50 | 1 year | Bi-weekly | Variable turbine strings | 2006 | [37] |
| Vansycle | Umatilla, OR | 38 | 24.9 | 456 | 50 | 1 year | Monthly | 126 m x 126 m | 1999 | [38] |
| Vantage | Kittitas, WA | 60 | 90 | 520 | 80 | 1 year | Monthly, a subset of 10 searched weekly during migration | 240 m x 240 m | 2010/ 2011 | [39] |
| Wild Horse | Kittitas, WA | 127 | 229 | 1,088 | 67 | 1 year | Monthly, weekly (fall, spring migration at 16 turbines) | 110 m from two turbines in plot | 2007 | [40] |
| Windy Flats (Windy Point II) | Klickitat, WA | 114 | 262.2 | 685 | 80 | 1 year | Monthly (spring, summer, fall, and winter), weekly (spring and fall migration) | 180 m x 180 m (120m at MET tower) | 2010 | [41] |
| **Northern Forest Biome** | | | | | | | | | | |
| Lempster (2010) | Sullivan, NH | 12 | 24 | 289 | 78 | 6 months | Weekly | 120 m x 130 m | 2010 | [42] |
| Maple Ridge (2007) | Lewis, NY | 195 | 321.75 | 1,736 | 80 | 7 months | Weekly | 130 m x 120 m | 2007 | [43] |
| Maple Ridge (2008) | Lewis, NY | 195 | 321.75 | 1,882 | 80 | 7 months | Weekly | 130 m x 120 m | 2008 | [44] |
| Noble Altona (2010) | Clinton, NY | 65 | 97.5 | 964 | 80 | Spring, summer, fall | Daily, weekly | 120 m x 120 m | 2010 | [45] |
| Noble Chateaugay (2010) | Franklin, NY | 71 | 106.5 | 600 | 80 | Spring, summer, fall | Weekly | 120 m x 120 m | 2010 | [46] |
| Noble Clinton (2008) | Clinton, NY | 67 | 100 | 1,158 | 80 | Spring, summer, fall | Daily (8 turbines), 3-day (8 turbines) weekly (7 turbines | 120 m x 120 m | 2008 | [47] |
| Noble Clinton (2009) | Clinton, NY | 67 | 100 | 1,774 | 80 | Spring, summer, fall | Daily (8 turbines), weekly (15 turbines), all turbines weekly from July 1 to August 15 | 120 m x 120 m | 2009 | [48] |
| Noble Ellenburg (2008) | Clinton, NY | 54 | 80 | 1,068 | 80 | Spring, summer, fall | Daily (6 turbines), 3-day (6 turbines), weekly (6 turbines) | 120 m x 120 m | 2008 | [49] |
| Noble Ellenburg (2009) | Clinton, NY | 54 | 80 | 1,359 | 80 | Spring, summer, fall | Daily (6 turbines), weekly (12 turbines), all turbines weekly from July 1 to August 15 | 120 m x 120 m | 2009 | [50] |
| **Pacific Biome** | | | | | | | | | | |
| Alite (2010) | Kern, CA | 8 | 24 | 317 | 80 | 1 year | Weekly (spring, fall), bi-monthly (summer, winter) | 200 m x 200 m | 2009/ 2010 | [51] |
| Diablo | Alameda, CA | 31 | 20.46 | 682 | 50 & 55 | 2 years | Monthly | 75 m x 75 m | 2005-2007 | [52, 53] |
| Dillon ^a^ | Riverside, CA | 45 | 45 | 471 | 69 | 1 year | Weekly, bi-monthly in winter | 200 m x 200 m | 2008/ 2009 | [54] |
| Pine Tree | Kern, CA | 90 | 135 | 25 | 65 | 1 year | Bi-weekly | n/a | 2009/ 2010 | [55] |
| Shiloh I | Solano, CA | 100 | 150 | 7,771 | 65 | 3 years | Weekly | 105-m radius | 2006-2009 | [56] |
| Shiloh II (Year 1) | Solano, CA | 75 | 150 | 1,293 | 115 (33 turbs), 125 (42 turbs) | 1 year | Weekly | 100 m radius | 2009/ 2010 | [57] |
| **Prairie Biome** | | | | | | | | | | |
| Barton Chapel | Jack, TX | 60 | 120 |  | 78 | 1 year | 10 turbines weekly, 20 monthly | 200 m x 200 m | 2009/ 2010 | [58] |
| Blue Sky Green Field | Fond du Lac, WI | 88 | 145 | 1,869 | 80 | Fall, spring | Daily(10 turbines), weekly (20 turbines) | 160 m x 160 m | 2008 | [59] |
| Buffalo Gap I | Nolan and Taylor, TX | 67 | 134 | 336 | 80 | 10 months | Every 3 weeks | 215 m x 215 m | 2006 | [60] |
| Buffalo Gap II | Nolan, TX | 155 | 233 |  | 80 | 14 months | Every 21 days | 215 m x 215 m | 2007/ 2008 | [61] |
| Buffalo Ridge (Phase I; 1996) | Lincoln and Pipestone, MN | 73 | 25.6 | 1,362 | 36 | 1 year | Bi-monthly (spring, summer, and fall) | 126 m x 126 m | 1996-1999 | [62] |
| Buffalo Ridge (Phase I; 1997) | Lincoln and Pipestone, MN | 73 | 25.6 | 1,362 | 36 | 1 year | Bi-monthly (spring, summer, and fall) | 126 m x 126 m | 1996-1999 | [62] |
| Buffalo Ridge (Phase I; 1998) | Lincoln and Pipestone, MN | 73 | 25.6 | 1,362 | 36 | 1 year | Bi-monthly (spring, summer, and fall) | 126 m x 126 m | 1996-1999 | [62] |
| Buffalo Ridge (Phase I; 1999) | Lincoln and Pipestone, MN | 73 | 25.6 | 1,362 | 36 | 1 year | Bi-monthly (spring, summer, and fall) | 126 m x 126 m | 1996-1999 | [62] |
| Buffalo Ridge (Phase II; 1998) | Lincoln and Pipestone, MN | 143 | 107.25 | 1,106 | 50 | 1 year | Bi-monthly (spring, summer, and fall) | 126 m x 126 m | 1996-1999 | [62] |
| Buffalo Ridge (Phase II; 1999) | Lincoln and Pipestone, MN | 143 | 107.25 | 1,106 | 50 | 1 year | Bi-monthly (spring, summer, and fall) | 126 m x 126 m | 1996-1999 | [62] |
| Buffalo Ridge (Phase III; 1999) | Lincoln and Pipestone, MN | 138 | 103.5 | 372 | 50 | 1 year | Bi-monthly (spring, summer, and fall) | 126 m x 126 m | 1996-1999 | [62] |
| Buffalo Ridge I (2010) | Brookings, SD | 24 | 50.4 | 432 | 79 | 1 year | Weekly (migratory), monthly (non-migratory) | 200 m x 200 m | 2009/ 2010 | [63] |
| Buffalo Ridge II (2011) | Brookings and Deuel, SD | 105 | 210 |  | 78 | 1 year | Weekly (spring, summer, fall), monthly (winter) | 100 m x 100 m | 2011/ 2012 | [64] |
| Cedar Ridge (2009) | Fond du Lac, WI | 41 | 67.6 | 1,702 | 80 | Spring, summer, fall | Daily, every 4 days; late fall searched every 3 days | 160 m x 160 m | 2009 | [65] |
| Cedar Ridge (2010) | Fond Du Lac, WI | 41 | 68 | 1,702 (2009) and 1,448 (2010) | 80 | 1 year | Five turbines were surveyed daily, 15 turbines surveyed every 4 days in rotating groups each day. All 20 surveyed every three days during late fall | 160 m x 160 m | 2010 | [66] |
| Elm Creek | Jackson, MN | 67 | 100 | 448 | 80 | 1 year | Weekly, monthly | 200 m x 200 m | 2009/ 2010 | [67] |
| Elm Creek II | Jackson and Martin, MN | 62 | 148.8 | 427 | 80 | 1 year | 20 searched every 28 days, 10 turbines every 7 days during migration) | 200 x 200m (2 random migration search areas 100 x 100m) | 2011/ 2012 | [68] |
| Grand Ridge I | LaSalle, IL | 66 | 99 | 441 | 80 | 1 year | Weekly, monthly | 160 m x 160 m | 2009/ 2010 | [69] |
| NPPD Ainsworth | Brown, NE | 36 | 59.4 |  | 70 | Spring, summer, fall | Bi-monthly | 220 m x 220 m | 2006 | [70] |
| Prairie Winds SD1 (Crow Lake) | Aurora, Brule, and Jerauld, SD | 108 | 162 |  | 80 | 1 year | Twice monthly (spring, summer, fall), monthly (winter) | 200 m x 200 m | 2011/ 2012 | [71] |
| Rugby | Pierce, ND | 71 | 149 | 442 | 78 | 1 year | Weekly (spring, fall; migratory turbines), monthly ( non-migratory turbines) | 200 m x 200 m | 2010 | [72] |
| Wessington Springs (2009) | Jerauld, SD | 34 | 51 | 319 | 80 | Spring, summer, fall | Bi-monthly | 200 m x 200 m | 2009 | [73] |
| Wessington Springs (2010) | Jerauld, SD | 34 | 51 | 357 | 80 | 8 months | Bi-weekly (spring, summer, fall) | 200 m x 200 m | 2010 | [74] |
| Winnebago | Winnebago, IA | 10 | 20 | 175 | 78 | 1 year | Weekly (migratory), monthly (non-migratory) | 200 m x 200 m | 2009/ 2010 | [75] |
| **Projects with All Bird Estimates (where small bird estimates were not available) by Avifaunal Biome Region.** | | | | | | | | | | |
| **Eastern Biome** | | | | | | | | | | |
| Buffalo Mountain (2000-2003) | Anderson, TN | 3 | 1.98 | 645 | 65 | 3 years | Bi-weekly, weekly, bi-monthly | 50-m radius | 2000-2003 | [76] |
| Buffalo Mountain (2005) | Anderson, TN | 18 | 28.98 | 40 | Model V47 = 65, V80 = 78 | 1 year | Bi-weekly, weekly, bi-monthly, and 2 to 5 day intervals | 50-m radius | 2005 | [77] |
| Casselman (2008) | Somerset, PA | 23 | 34.5 | 2,040 | 80 | 7 months | Daily | 126 m x 120 m | 2008 | [78] |
| Casselman (2009) | Somerset, PA | 23 | 34.5 | 2,241 | 80 | 7.5 months | Daily | 126 m x 120 m | 2009 | [79] |
| Cohocton/Dutch Hill (2009) | Steuben, NY | 50 | 125 | 1,087 (804 (daily), 283 (weekly)) | 80 | Spring, summer, fall | Daily (5 turbines), weekly (12 turbines) | 130 m x 130 m | 2009 | [80] |
| Cohocton/Dutch Hills (2010) | Steuben, NY | 50 | 125 | 664 | 80 | Spring, summer, fall | Daily, weekly | 120 m x 120 m | 2010 | [81] |
| Locust Ridge II (2009) | Columbia and Schuykill, PA | 51 | 102 |  | 80 | 6.5 months | Daily | 126 m x 120 m | 2009 | [82] |
| Locust Ridge II (2010) | Columbia and Schuykill, PA | 51 | 102 |  | 80 | 6.5 months | Daily | 126 m x 120 m | 2010 | [82] |
| Mountaineer (2003) | Tucker, WV | 44 | 66 | 954 | 80 | 7 months | Weekly, monthly | 60-m radius | 2003 | [83] |
| Munnsville (2008) | Madison and Oneida, NY | 23 | 34.5 |  | 69.5 | Spring, summer, fall | Weekly | 120 m x 120 m | 2008 | [84] |
| Ripley (2008) | Bruce, Ontario | 38 | 76 |  | 64 | Spring, fall | Twice weekly for odd turbines; weekly for even turbines. | 80 m x 80 m | 2008 | [85] |
| Sheldon (2010) | Wyoming, NY | 75 | 112.5 | 2,188 | 80 | 7 months | Daily (8 turbines), weekly (17 turbines) | 115 m x 115 m | 2010 | [86] |
| Sheldon (2011) | Wyoming, NY | 75 | 112.5 | 2,136 | 80 | 7 months | Daily (8 turbines), weekly (17 turbines) | 115 m x 115 m | 2011 | [87] |
| **Intermountain West Biome** | | | | | | | | | | |
| Big Horn | Klickitat, WA | 133 | 199.5 | 2,261 | 80 | 1 year | Bi-monthly (spring, fall), monthly (winter, summer) | 180 m x 180 m | 2006/ 2007 | [88] |
| Harvest Wind (2010-2012) | Klickitat, WA | 43 | 98.9 | 184 | 80 | 2 years | Twice a week, weekly and monthly | 180 m x 180 m & 240 m x 240 m | 2010/ 2011 | [89] |
| Leaning Juniper | Gilliam, OR | 67 | 100.5 | 629 | 80 | 2 years | Bi-monthly (spring, fall), monthly (winter, summer) | 240 m x 240 m | 2006-2008 | [90] |
| Pebble Springs | Gilliam, OR | 47 | 98.7 | 320 | 79 | 1 year | Bi-monthly (spring, fall), monthly (winter, summer) | 180 m x 180 m | 2009-2010 | [91] |
| Summerview (2006) | Alberta | 39 | 70.2 | 2,574 | 67 | 1 year | Weekly, bi-weekly (May to July, September) | 140 m x 140 m | 2005/ 2006 | [92] |
| Tuolumne (Windy Point I) | Klickitat, WA | 62 | 136.6 | 509 | 80 | 1 year | Monthly throughout the year, a sub-set of 10 turbines were also searched weekly during the spring, summer, and fall | 180 m x 180 m | 2009/ 2010 | [93] |
| White Creek (2007-2011) | Klickitat, WA | 89 | 204.7 | 348 | 80 | 4 years | Twice a week, weekly and monthly | 180 m x 180 m & 240 m x 240 m | 2007-2011 | [94] |
| **Northern Forest Biome** | | | | | | | | | | |
| Lempster (2009) | Sullivan, NH | 12 | 24 | 598 | 78 | 6 months | Daily | 120 m x 130 m | 2009 | [95] |
| Mars Hill (2007) | Aroostook, ME | 28 | 42 |  | 80.5 | Spring, summer, fall | Daily (2 random turbines), weekly (all turbines): extended plot searched once per season | 76-m diameter, extended plot 238-m diameter | 2007 | [96] |
| Mars Hill (2008) | Aroostook, ME | 28 | 42 |  | 80.5 | Spring, summer, fall | Weekly: extended plot searched once per season | 76-m diameter, extended plot 238-m diameter | 2008 | [97] |
| Stetson Mountain I (2009) | Penobscot and Washington, ME | 38 | 57 | 506 | 80 | 27 weeks (spring, summer, fall) | Weekly | 76-m diameter | 2009 | [98] |
| Stetson Mountain I (2011) | Washington, ME | 38 | 57 | 513 | 80 | 6 months | Weekly | Varied | 2011 | [99] |
| Stetson Mountain II (2010) | Washington, ME | 17 | 25.5 | 552 | 80 | 6 months | Weekly (3 turbines twice a week) | Varied | 2010 | [100] |
| **Pacific Biome** | | | | | | | | | | |
| High Winds (2004) | Solano, CA | 90 | 162 | 2,070 | 60 | 1 year | Bi-monthly | 75-m radius | 2003/ 2004 | [101] |
| High Winds (2005) | Solano, CA | 90 | 162 | 2,150 | 60 | 1 year | Bi-monthly | 75-m radius | 2004/ 2005 | [101] |
| **Prairie Biome** | | | | | | | | | | |
| Barton I and II | Worth, IA | 80 | 160 | 405 | 100 | 1 year | Weekly (spring, fall; migratory turbines), monthly (summer, winter; non-migratory turbines) | 200 m x 200 m | 2010/ 2011 | [102] |
| Kewaunee County | Kewaunee, WI | 31 | 20.46 |  | 65 | 2 years | Bi-weekly (spring, summer), daily (spring, fall migration), weekly (fall, winter) | 60 m x 60 m | 1999-2001 | [103] |
| Moraine II | Pipestone and Murray, MN | 33 | 49.5 | 440 | 82.5 | 1 year | Weekly (migratory), monthly (non-migratory) | 200 m x 200 m | 2009 | [104] |
| Pioneer Prairie I (Phase II) | Mitchell, IA | 62 | 102.3 |  | 80 | 1 year | Weekly (spring and fall), every two weeks (summer), monthly (winter) | 80 m x 80 m | 2011/ 2012 | [105] |
| Prairie Winds ND1/Minot (2010) | Ward, ND | 80 | 115.5 |  | 89 | 3 seasons | Bi-monthly | Minimum of 100 m x 100 m | 2010 | [106] |
| Prairie Winds ND1/Minot (2011) | Ward, ND | 80 | 115.5 |  | 80 | 3 seasons | Twice monthly | Minimum of 100 m x 100 m | 2011 | [107] |
| Top of Iowa (2003) | Worth, IA | 89 | 80 | 6,214 | 71.6 | Spring, summer, fall | Once every 2 to 3 days | 76 m x 76 m | 2003 | [108] |
| Top of Iowa (2004) | Worth, IA | 89 | 80 | 6,214 | 71.6 | Spring, summer, fall | Once every 2 to 3 days | 76 m x 76 m | 2004 | [108] |
| ^a^ The Dillon Project was the only project in the Southwestern Biome represented by an available fatality report. Due to its singularity and since it is located very close to the Pacific Biome; it was combined with the Pacific Biome data. | | | | | | | | | | |

# References:

1. Young, D.P. Jr., M. Lout, Z. Courage, S. Nomani, and K. Bay. 2012. 2011 Post-Construction Monitoring Study, Criterion Wind Project, Garrett County, Maryland: April - November 2011. Revised November 25, 2013.

2. Young, D.P. Jr., Bay K, Nomani S, Tidhar W (2009) Nedpower Mount Storm Wind Energy Facility, Post-Construction Avian and Bat Monitoring: March - June 2009.

3. Young, D.P. Jr., Bay K, Nomani S, Tidhar W (2010) Nedpower Mount Storm Wind Energy Facility, Post-Construction Avian and Bat Monitoring: July - October 2009.

4. Young, D.P. Jr., Bay K, Nomani S, Tidhar W (2010) Nedpower Mount Storm Wind Energy Facility, Post-Construction Avian and Bat Monitoring: April - July 2010.

5. Young, D.P. Jr., Nomani S, Tidhar W, Bay K (2011) Nedpower Mount Storm Wind Energy Facility, Post-Construction Avian and Bat Monitoring: July - October 2010.

6. Young, D.P. Jr., Nomani S, Courage Z, Bay K (2011) Nedpower Mount Storm Wind Energy Facility, Post-Construction Avian and Bat Monitoring: April - July 2011.

7. Young, D.P. Jr., Nomani S, Courage Z, Bay K (2012) Nedpower Mount Storm Wind Energy Facility, Post-Construction Avian and Bat Monitoring: July - October 2011.

8. Jain A, Kerlinger P, Curry R, Slobodnik L, Quant J, et al. (2009) Annual Report for the Noble Bliss Windpark, LLC, Postconstruction Bird and Bat Fatality Study - 2008.

9. Jain A, Kerlinger P, Slobodnik L, Curry R, Fuerst A, et al. (2010) Annual Report for the Noble Bliss Windpark, LLC: Postconstruction Bird and Bat Fatality Study - 2009.

10. Jain A, Kerlinger P, Slobodnik L, Curry R, Harte A (2011) Annual Report for the Noble Wethersfield Windpark, LLC: Postconstruction Bird and Bat Fatality Study - 2010.

11. Jeffrey JD, Bay K, Erickson WP, Sonneberg M, Baker J, et al. (2009) Portland General Electric Biglow Canyon Wind Farm Phase I Post-Construction Avian and Bat Monitoring First Annual Report, Sherman County, Oregon. January 2008 - December 2008.

12. Enk T, Bay K, Sonnenberg M, Baker J, Kesterke M, et al. (2010) Biglow Canyon Wind Farm Phase I Post-Construction Avian and Bat Monitoring Second Annual Report, Sherman County, Oregon. January 26, 2009 - December 11, 2009.

13. Enk T, Bay K, Sonnenberg M, Flaig J, Boehrs JR, et al. (2011) Year 1 Post-Construction Avian and Bat Monitoring Report: Biglow Canyon Wind Farm Phase II, Sherman County, Oregon. September 10, 2009 - September 12, 2010.

14. Enk T, Bay K, Sonnenberg M, Boehrs JR (2012) Year 2 Avian and Bat Monitoring Report: Biglow Canyon Wind Farm Phase II, Sherman County, Oregon. September 13, 2010 - September 12, 2011.

15. Enk T, Bay K, Sonnenberg M, Boehrs JR (2012) Year 1 Avian and Bat Monitoring Report: Biglow Canyon Wind Farm Phase III, Sherman County, Oregon. September 13, 2010 - September 9, 2011.

16. Young, D.P. Jr., Jeffrey J, Erickson WP, Bay K, Poulton VK, et al. (2006) Eurus Combine Hills Turbine Ranch. Phase 1 Post Construction Wildlife Monitoring First Annual Report: February 2004 - February 2005.

17. Enz T, Bay K, Sonnenberg M, Palochak A (2012) Post-Construction Monitoring Studies for the Combine Hills Turbine Ranch, Umatilla County, Oregon. Final Report: January 7 - December 2, 2011.

18. Thompson J, Solick D, Bay K (2011) Post-Construction Fatality Surveys for the Dry Lake Phase I Wind Project. Iberdrola Renewables: September 2009 - November 2010.

19. Thompson J, Bay K (2012) Post-Construction Fatality Surveys for the Dry Lake II Wind Project: February 2011 – February 2012.

20. Jeffrey JD, Erickson WP, Bay K, Sonneberg M, Baker J, et al. (2009) Horizon Wind Energy, Elkhorn Valley Wind Project, Post-Construction Avian and Bat Monitoring, First Annual Report, January-December 2008.

21. Enk T, Derby C, Bay K, Sonnenberg M (2011) 2010 Post-Construction Fatality Monitoring Report, Elkhorn Valley Wind Farm, Union County, Oregon. January – December 2010.

22. Young, D.P. Jr., Erickson WP, Good RE, Strickland MD, Johnson GD (2003) Avian and Bat Mortality Associated with the Initial Phase of the Foote Creek Rim Windpower Project, Carbon County, Wyoming, Final Report, November 1998 - June 2002.

23. URS Corporation (2010) Final Goodnoe Hills Wind Project Avian Mortality Monitoring Report.

24. Gritski R, Kronner K (2010) Hay Canyon Wind Power Project Wildlife Monitoring Study: May 2009 - May 2010.

25. Young, D.P. Jr., Erickson WP, Jeffrey J, Poulton VK (2007) Puget Sound Energy Hopkins Ridge Wind Project Phase 1 Post-Construction Avian and Bat Monitoring First Annual Report, January - December 2006. 25 pp. p.

26. Young, D.P. Jr., Jeffrey JD, Bay K, Erickson WP (2009) Puget Sound Energy Hopkins Ridge Wind Project, Phase 1, Columbia County, Washington. Post-Construction Avian and Bat Monitoring, Second Annual Report: January - December, 2008.

27. Stantec Consulting Services, Inc. (Stantec Consulting Services) (2012) Post-Construction Monitoring, Summer 2011 - Spring 2012, Year 1 Annual Report: Kittitas Valley Wind Power Project, Cle Elum, Washington.

28. Johnson GD, Erickson WP, White J (2003) Avian and Bat Mortality During the First Year of Operation at the Klondike Phase I Wind Project, Sherman County, Oregon.

29. Northwest Wildlife Consultants, Inc. (NWC), Western EcoSystems Technology, Inc. (WEST) (2007) Avian and Bat Monitoring Report for the Klondike II Wind Power Project. Sherman County, Oregon.

30. Gritski R, Downes S, Kronner K (2010) Klondike III (Phase 1) Wind Power Project Wildlife Monitoring: October 2007-October 2009.

31. Gritski R, Downes S, Kronner K (2011) Klondike IIIa (Phase 2) Wind Power Project Wildlife Monitoring: August 2008 - August 2010.

32. Enz T, Bay K (2011) Post-Construction Monitoring at the Linden Ranch Wind Farm, Klickitat County, Washington. Final Report: June 30, 2010 - July 17, 2011.

33. URS Corporation (2010) Final Marengo I Wind Project Year One Avian Mortality Monitoring Report.

34. URS Corporation (2010) Final Marengo II Wind Project Year One Avian Mortality Monitoring Report.

35. Erickson WP, Kronner K, Gritski R (2003) Nine Canyon Wind Power Project Avian and Bat Monitoring Report. September 2002 – August 2003.

36. Erickson WP, Jeffrey J, Kronner K, Bay K (2004) Stateline Wind Project Wildlife Monitoring Annual Report. July 2001 - December 2003.

37. Erickson WP, Kronner K, Bay KJ (2007) Stateline 2 Wind Project Wildlife Monitoring Report, January - December 2006.

38. Erickson WP, Johnson GD, Strickland MD, Kronner K (2000) Avian and Bat Mortality Associated with the Vansycle Wind Project, Umatilla County, Oregon: 1999 Study Year.

39. Ventus Environmental Solutions (Ventus) (2012) Vantage Wind Energy Center Avian and Bat Monitoring Study: March 2011- March 2012.

40. Erickson WP, Jeffrey J, Poulton VK (2008) Avian and Bat Monitoring: Year 1 Report. Puget Sound Energy Wild Horse Wind Project, Kittitas County, Washington.

41. Enz T, Bay K, Nomani S, Kesterke M (2011) Bird and Bat Fatality Monitoring Study, Windy Flats and Windy Point II Wind Energy Projects, Klickitat County, Washington. Final Report: February 1, 2010 - January 14, 2011.

42. Tidhar D, Tidhar WL, McManus L, Courage Z (2011) 2010 Post-Construction Fatality Surveys for the Lempster Wind Project, Lempster, New Hampshire.

43. Jain A, Kerlinger P, Curry R, Slobodnik L (2009) Annual Report for the Maple Ridge Wind Power Project: Post-Construction Bird and Bat Fatality Study - 2007.

44. Jain A, Kerlinger P, Curry R, Slobodnik L, Lehman M (2009) Maple Ridge Wind Power Avian and Bat Fatality Study Report - 2008.

45. Jain A, Kerlinger P, Slobodnik L, Curry R, Russell K (2011) Annual Report for the Noble Altona Windpark, LLC: Postconstruction Bird and Bat Fatality Study - 2010.

46. Jain A, Kerlinger P, Slobodnik L, Curry R, Russell K (2011) Annual Report for the Noble Chateaugay Windpark, LLC: Postconstruction Bird and Bat Fatality Study - 2010.

47. Jain A, Kerlinger P, Curry R, Slobodnik L, Histed J, et al. (2009) Annual Report for the Noble Clinton Windpark, LLC, Postconstruction Bird and Bat Fatality Study - 2008.

48. Jain A, Kerlinger P, Slobodnik L, Curry R, Russell K (2010) Annual Report for the Noble Clinton Windpark, LLC: Postconstruction Bird and Bat Fatality Study - 2009.

49. Jain A, Kerlinger P, Curry R, Slobodnik L, Fuerst A, et al. (2009) Annual Report for the Noble Ellenburg Windpark, LLC, Postconstruction Bird and Bat Fatality Study - 2008.

50. Jain A, Kerlinger P, Slobodnik L, Curry R, Russell K (2010) Annual Report for the Noble Ellenburg Windpark, LLC: Postconstruction Bird and Bat Fatality Study - 2009.

51. Chatfield A, Erickson WP, Bay K (2010) Final Report: Avian and Bat Fatality Study at the Alite Wind-Energy Facility, Kern County, California. Final Report: June 15, 2009 – June 15, 2010.

52. Western EcoSystems Technology, Inc. (WEST) (2006) Diablo Winds Wildlife Monitoring Progress Report, March 2005 - February 2006. Cheyenne, Wyoming: WEST.

53. Western EcoSystems Technology, Inc. (WEST) (2008) Diablo Winds Wildlife Monitoring Progress Report: March 2005 – February 2007.

54. Chatfield A, Erickson W, Bay K (2009) Avian and Bat Fatality Study, Dillon Wind-Energy Facility, Riverside County, California. Final Report: March 26, 2008 - March 26, 2009.

55. BioResource Consultants, Inc. (BRC) (2010) 2009/2010 Annual Report: Bird and Bat Mortality Monitoring, Pine Tree Wind Farm, Kern County, California.

56. Kerlinger P, Curry R, Culp L, Hasch A, Jain A (2009) Post-Construction Avian Monitoring Study for the Shiloh I Wind Power Project, Solano County, California. Final Report: October 2009.

57. Kerlinger P, Curry R, Culp L, Hasch A, Jain A (2010) Post-Construction Avian Monitoring Study for the Shiloh II Wind Power Project, Solano County, California. Year One Report.

58. Western EcoSystems Technology, Inc. (WEST) (2011) Post-Construction Fatality Surveys for the Barton Chapel Wind Project: Iberdrola Renewables. Version: July 2011.

59. Gruver J, Sonnenberg M, Bay K, Erickson W (2009) Post-Construction Bat and Bird Fatality Study at the Blue Sky Green Field Wind Energy Center, Fond Du Lac County, Wisconsin July 21 - October 31, 2008 and March 15 - June 4, 2009.

60. Tierney R (2007) Buffalo Gap I Wind Farm Avian Mortality Study: February 2006-January 2007.

61. Tierney R (2009) Buffalo Gap 2 Wind Farm Avian Mortality Study: July 2007 - December 2008. Final Survey Report.

62. Johnson GD, Erickson WP, Strickland MD, Shepherd MF, Shepherd DA (2000) Avian Monitoring Studies at the Buffalo Ridge Wind Resource Area, Minnesota: Results of a 4-Year Study. 212 pp.

63. Derby C, Chodachek K, Bay K, Merrill A (2010) Post-Construction Fatality Survey for the Buffalo Ridge I Wind Project. May 2009 - May 2010.

64. Derby C, Chodachek K, Sonnenberg M (2012) Post-Construction Casualty Surveys for the Buffalo Ridge II Wind Project. Iberdrola Renewables: March 2011- February 2012.

65. BHE Environmental, Inc. (BHE) (2010) Post-Construction Bird and Bat Mortality Study: Cedar Ridge Wind Farm, Fond Du Lac County, Wisconsin.

66. BHE Environmental, Inc. (BHE) (2011) Post-Construction Bird and Bat Mortality Study: Cedar Ridge Wind Farm, Fond Du Lac County, Wisconsin.

67. Derby C, Chodachek K, Bay K, Merrill A (2010) Post-Construction Fatality Surveys for the Elm Creek Wind Project: March 2009- February 2010.

68. Derby C, Chodachek K, Sonnenberg M (2012) Post-Construction Fatality Surveys for the Elm Creek II Wind Project. Iberdrola Renewables: March 2011-February 2012.

69. Derby C, Ritzert J, Bay K (2010) Bird and Bat Fatality Study, Grand Ridge Wind Resource Area, Lasalle County, Illinois. January 2009 - January 2010.

70. Derby C, Dahl A, Erickson W, Bay K, Hoban J (2007) Post-Construction Monitoring Report for Avian and Bat Mortality at the NPPD Ainsworth Wind Farm. Unpublished report prepared by Western EcoSystems Technology, Inc. (WEST), Cheyenne, Wyoming, for the Nebraska Public Power District.

71. Derby C, Dahl A, Merrill A (2012) Post-Construction Monitoring Results for the PrairieWinds SD1 Wind Energy Facility, South Dakota. Final Report: March 2011 - February 2012.

72. Derby C, Chodachek K, Bay K, Nomani S (2011) Post-Construction Fatality Surveys for the Rugby Wind Project: Iberdrola Renewables, Inc. March 2010 - March 2011.

73. Derby C, Dahl A, Merrill A, Bay K (2010) 2009 Post-Construction Monitoring Results for the Wessington Springs Wind-Energy Facility, South Dakota. Final Report.

74. Derby C, Dahl A, Bay K, McManus L (2011) 2010 Post-Construction Monitoring Results for the Wessington Springs Wind Energy Facility, South Dakota. Final Report: March 9 – November 16, 2010.

75. Derby C, Chodachek K, Bay K, Merrill A (2010) Post-Construction Fatality Surveys for the Winnebago Wind Project: March 2009- February 2010.

76. Nicholson CP, R.D. Tankersley J, Fiedler JK, Nicholas NS (2005) Assessment and Prediction of Bird and Bat Mortality at Wind Energy Facilities in the Southeastern United States.

77. Fiedler JK, Henry TH, Tankersley RD, Nicholson CP (2007) Results of Bat and Bird Mortality Monitoring at the Expanded Buffalo Mountain Windfarm, 2005.

78. Arnett EB, Schirmacher MR, Huso MMP, Hayes JP (2009) Effectiveness of Changing Wind Turbine Cut-in Speed to Reduce Bat Fatalities at Wind Facilities: 2008 Annual Report.

79. Arnett EB, Schirmacher MR, Huso MMP, Hayes JP (2010) Patterns of Bat Fatality at the Casselman Wind Project in South-Central Pennsylvania.

80. Stantec Consulting, Inc. (Stantec) (2010) Cohocton and Dutch Hill Wind Farms Year 1 Post-Construction Monitoring Report, 2009, for the Cohocton and Dutch Hill Wind Farms in Cohocton, New York.

81. Stantec Consulting, Inc. (Stantec) (2011) Cohocton and Dutch Hill Wind Farms Year 2 Post-Construction Monitoring Report, 2010, for the Cohocton and Dutch Hill Wind Farms in Cohocton, New York.

82. Arnett EB, Schirmacher MR, Hein CD, Huso MMP (2011) Patterns of Bird and Bat Fatality at the Locust Ridge II Wind Project, Pennsylvania. 2009-2010 Final Report.

83. Kerns J, Kerlinger P (2004) A Study of Bird and Bat Collisions at the Mountaineer Wind Energy Facility, Tucker County, West Virginia: Annual Report for 2003. Technical report prepared by Curry and Kerlinger, LLC., for FPL Energy and Mountaineer Wind Energy Center Technical Review Committee. Curry and Kerlinger, LLC. 39 pp p.

84. Stantec Consulting, Inc. (Stantec) (2009) Post-Construction Monitoring at the Munnsville Wind Farm, New York: 2008.

85. Jacques Whitford Stantec Limited (Jacques Whitford) (2009) Ripley Wind Power Project Postconstruction Monitoring Report.

86. Tidhar D, McManus L, Courage Z, Tidhar WL (2012) 2010 Post-Construction Fatality Monitoring Study and Bat Acoustic Study for the High Sheldon Wind Farm, Wyoming County, New York. Final Report: April 15 - November 15, 2010.

87. Tidhar D, McManus L, Solick D, Courage Z, Bay K (2012) 2011 Post-Construction Fatality Monitoring Study and Bat Acoustic Study for the High Sheldon Wind Farm, Wyoming County, New York. Final Report: April 15 - November 15, 2011.

88. Kronner K, Gritski R, Downes S (2008) Big Horn Wind Power Project Wildlife Fatality Monitoring Study: 2006−2007.

89. Downes S, Gritski R (2012) Harvest Wind Project Wildlife Monitoring Report: January 2010 – January 2012.

90. Gritski R, Kronner K, Downes S (2008) Leaning Juniper Wind Power Project, 2006 − 2008. Wildlife Monitoring Final Report.

91. Gritski R, Kronner K (2010) Pebble Springs Wind Power Project Wildlife Monitoring Study: January 2009 - January 2010.

92. Brown WK, Hamilton BL (2006) Monitoring of Bird and Bat Collisions with Wind Turbines at the Summerview Wind Power Project, Alberta: 2005-2006.

93. Enz T, Bay K (2010) Post-Construction Avian and Bat Fatality Monitoring Study, Tuolumne Wind Project, Klickitat County, Washington. Final Report: April 20, 2009 - April 7, 2010.

94. Downes S, Gritski R (2012) White Creek Wind I Wildlife Monitoring Report: November 2007 - November 2011.

95. Tidhar D, Tidhar W, Sonnenberg M (2010) Post-Construction Fatality Surveys for Lempster Wind Project, Iberdrola Renewables. Prepared for Lempster Wind, LLC, Lempster Wind Technical Advisory Committee, and Iberdrola Renewables, Inc.

96. Stantec Consulting, Inc. (Stantec) (2008) 2007 Spring, Summer, and Fall Post-Construction Bird and Bat Mortality Study at the Mars Hill Wind Farm, Maine.

97. Stantec Consulting, Inc. (Stantec) (2009) Post-Construction Monitoring at the Mars Hill Wind Farm, Maine - Year 2, 2008.

98. Stantec Consulting, Inc. (Stantec) (2009) Stetson I Mountain Wind Project: Year 1 Post-Construction Monitoring Report, 2009 for the Stetson Mountain Wind Project in Penobscot and Washington Counties, Maine.

99. Normandeau Associates, Inc. (2011) Year 3 Post- Construction Avian and Bat Casualty Monitoring at the Stetson I Wind Farm, T8 R4 NBPP, Maine.

100. Normandeau Associates, Inc. (2010) Stetson Mountain II Wind Project Year 1 Post-Construction Avian and Bat Mortality Monitoring Study, T8 R4 NBPP, Maine.

101. Kerlinger P, Curry R, Culp L, Jain A, Wilkerson C, et al. (2006) Post-Construction Avian and Bat Fatality Monitoring for the High Winds Wind Power Project, Solano County, California: Two Year Report.

102. Derby C, Chodachek K, Bay K, Nomani S (2011) Post-Construction Fatality Surveys for the Barton I and II Wind Project: IRI. March 2010 - February 2011.

103. Howe RW, Evans W, Wolf AT (2002) Effects of Wind Turbines on Birds and Bats in Northeastern Wisconsin. 104 pp p.

104. Derby C, Chodachek K, Bay K, Merrill A (2010) Post-Construction Fatality Surveys for the Moraine II Wind Project: March - December 2009.

105. Chodachek K, Derby C, Sonnenberg M, Thorn T (2012) Post-Construction Fatality Surveys for the Pioneer Prairie Wind Farm I LLC Phase II, Mitchell County, Iowa: April 4, 2011 – March 31, 2012.

106. Derby C, Chodachek K, Thorn T, Bay K, Nomani S (2011) Post-Construction Fatality Surveys for the PrairieWinds ND1 Wind Facility, Basin Electric Power Cooperative, March - November 2010.

107. Derby C, Chodachek K, Thorn T, Merrill A (2012) Post-Construction Surveys for the PrairieWinds ND1 (2011) Wind Facility Basin Electric Power Cooperative: March - October 2011.

108. Jain A (2005) Bird and Bat Behavior and Mortality at a Northern Iowa Windfarm. Thesis. Ames, Iowa: Iowa State University.
