# Supplementary material for: A Comprehensive Analysis of Small-Passerine Fatalities from Collision with Turbines at Wind Energy Facilities
Source: PLoS One. 2014 Sep 15;9(9):e107491. doi: 10.1371/journal.pone.0107491 (PMC4164633; doi:10.1371/journal.pone.0107491)
Supplement: Appendix S2 — Bird fatality monitoring studies at wind energy facilities with estimates of fatality rates for small birds, confidence intervals (CI) and estimator used, if known, for avifaunal biomes in the United States and Canada. Blank spaces indicate that data was not available. (DOCX) [file pone.0107491.s029.docx]

**Appendix S2. Bird fatality monitoring studies at wind energy facilities with estimates of fatality rates for small birds, confidence intervals (CI) and estimator used, if known, for avifaunal biomes in the United States and Canada.** Blank spaces indicate that data was not available.

| **Project Name** | **Small Bird Fatality Estimate (MW/year)** | **CI (MW/year)** | **Estimator Used** | **References** |
| --- | --- | --- | --- | --- |
| **Eastern Biome** | | | | |
| Criterion (2011) | 6.1 | (4.20-9.11) | Shoenfeld | [1] |
| Mount Storm (2009) | 3.1 | (1.94-4.49) | Shoenfeld | [2, 3] |
| Mount Storm (2010) | 2.19 | (1.69-3.05) | Shoenfeld | [4, 5] |
| Mount Storm (2011) | 3.93 | (2.79-6.12) | Shoenfeld | [6, 7] |
| Noble Bliss (2008) | 1.09 |  | Jain | [8] |
| Noble Bliss (2009) | 1.82 |  | Jain | [9] |
| Noble Wethersfield (2010) | 1.51 |  | Jain | [10] |
| **Intermountain West Biome** | | | | |
| Biglow Canyon (Phase I; 2008) | 1.54 | (0.90-2.46) | Shoenfeld | [11] |
| Biglow Canyon (Phase I; 2009) | 2.33 | (1.72-3.16) | Shoenfeld | [12] |
| Biglow Canyon (Phase II; 2009/2010) | 5.16 | (3.07-8.98) | Shoenfeld | [13] |
| Biglow Canyon (Phase II; 2010/2011) | 2.48 | (1.57-5.67) | Shoenfeld | [14] |
| Biglow Canyon (Phase III; 2010/2011) | 2.17 | (1.38-4.60) | Shoenfeld | [15] |
| Combine Hills (2004/2005) | 1.89 | (1.10-2.89) | Shoenfeld | [16] |
| Combine Hills (2011) | 1.96 | (1.26-3.41) | Shoenfeld | [17] |
| Dry Lake I | 2.13 | (1.22-3.45) | Shoenfeld | [18] |
| Dry Lake II | 1.30 | (0.53-2.96) | Shoenfeld | [19] |
| Elkhorn (2008) | 0.45 | (0.29-0.70) | Shoenfeld | [20] |
| Elkhorn (2010) | 1.76 | (0.96-2.99) | Shoenfeld | [21] |
| Foote Creek Rim (Phase I; 1999) | 3.00 | (1.37-4.62) | Shoenfeld | [22] |
| Foote Creek Rim (Phase I; 2000) | 2.15 | (0.72-3.57) | Shoenfeld | [22] |
| Foote Creek Rim (Phase I; 2001-2002) | 1.71 | (0.24-3.19) | Shoenfeld | [22] |
| Goodnoe | 1.02 |  | Shoenfeld | [23] |
| Hay Canyon | 1.92 | (0.68-4.19) | Huso | [24] |
| Hopkins Ridge (2006) | 0.8 | (0.52-1.19) | Shoenfeld | [25] |
| Hopkins Ridge (2008) | 2.43 | (1.41-4.67) | Shoenfeld | [26] |
| Kittitas Valley (2011-2012) | 0.72 | (0.48-1.02) | Huso | [27] |
| Klondike | 0.77 | (0.27-1.40) | Shoenfeld | [28] |
| Klondike II | 2.90 | (1.86-4.45) | Shoenfeld | [29] |
| Klondike III (Phase I) | 2.60 | (1.97-3.77) | Shoenfeld | [30] |
| Klondike IIIa (Phase II) | 1.92 | (0.55-4.34) | Huso | [31] |
| Linden Ranch | 4.83 | (2.86-9.79) | Shoenfeld | [32] |
| Marengo I (2009) | 0.23 |  | Shoenfeld | [33] |
| Marengo II (2009) | 0.16 |  | Shoenfeld | [34] |
| Nine Canyon | 2.55 | (1.34-4.23) | Shoenfeld | [35] |
| Stateline (2002) | 2.85 | (2.16-3.43) | Shoenfeld | [36] |
| Stateline (2003) | 2.29 | (1.57-2.87) | Shoenfeld | [36] |
| Stateline (2006) | 0.95 | (0.46-1.50) | Shoenfeld | [37] |
| Vansycle | 0.75 | (0.72-0.80) | Naïve | [38] |
| Vantage | 1.14 |  | Shoenfeld | [39] |
| Wild Horse | 1.28 |  | Shoenfeld | [40] |
| Windy Flats (Windy Point II) | 7.59 |  | Shoenfeld | [41] |
| **Northern Forest Biome** | | | | |
| Lempster (2010) | 1.65 | (0.77-2.95) | Shoenfeld | [42] |
| Maple Ridge (2007) | 2.73 |  | Jain | [43] |
| Maple Ridge (2008) | 1.86 |  | Jain | [44] |
| Noble Altona (2010) | 1.68 |  | Jain | [45] |
| Noble Chateaugay (2010) | 1.01 |  | Jain | [46] |
| Noble Clinton (2008) | 1.39 |  | Jain | [47] |
| Noble Clinton (2009) | 0.57 |  | Jain | [48] |
| Noble Ellenburg (2008) | 0.66 |  | Jain | [49] |
| Noble Ellenburg (2009) | 2.02 |  | Jain | [50] |
| **Pacific Biome** | | | | |
| Alite (2010) | 0.24 | (0.00-0.56) | Shoenfeld | [51] |
| Diablo Winds | 3.67 |  | Shoenfeld | [52, 53] |
| Dillon ^a^ | 4.26 | (2.76-6.50) | Shoenfeld | [54] |
| Pine Tree | 3.87 |  | Shoenfeld | [55] |
| Shiloh I | 4.86 |  | Jain | [56] |
| Shiloh II (Year 1) | 0.81 |  | Jain | [57] |
| **Prairie Biome** | | | | |
| Barton Chapel | 0.62 | (0-2.11) | Shoenfeld | [58] |
| Blue Sky Green Field | 6.96 | (5.32-9.63) | Shoenfeld | [59] |
| Buffalo Gap I | 1.08 |  | Shoenfeld | [60] |
| Buffalo Gap II | 0.07 |  | Shoenfeld | [61] |
| Buffalo Ridge (Phase I; 1996) | 4.14 | (0.94-7.34) | Naïve | [62] |
| Buffalo Ridge (Phase I; 1997) | 1.40 | (0.11-3.34) | Naïve | [62] |
| Buffalo Ridge (Phase I; 1998) | 2.40 | (0.06-4.71) | Naïve | [62] |
| Buffalo Ridge (Phase I; 1999) | 1.43 | (0.13-3.43) | Naïve | [62] |
| Buffalo Ridge (Phase II; 1998) | 2.28 | (0.89-3.67) | Naïve | [62] |
| Buffalo Ridge (Phase II; 1999) | 3.04 | (0.69-5.87) | Naïve | [62] |
| Buffalo Ridge (Phase III; 1999) | 5.93 | (0.15-11.71) | Naïve | [62] |
| Buffalo Ridge I (2010) | 4.16 | (1.66-7.53) | Shoenfeld | [63] |
| Buffalo Ridge II (2011) | 1.12 |  | Shoenfeld | [64] |
| Cedar Ridge (2009) | 5.21 |  | Huso | [65] |
| Cedar Ridge (2010) | 2.21 |  | Shoenfeld | [66] |
| Elm Creek | 1.07 | (0.00-2.94) | Shoenfeld | [67] |
| Elm Creek II | 3.60 |  | Shoenfeld | [68] |
| Grand Ridge I | 0 |  | Shoenfeld | [69] |
| NPPD Ainsworth | 1.51 | (0.79-2.57) | Shoenfeld | [70] |
| Prairie Winds SD1/Crow Lake | 0.96 |  | Shoenfeld | [71] |
| Rugby | 1.03 | (0.37-2.02) | Shoenfeld | [72] |
| Wessington Springs (2009) | 7.67 |  | Shoenfeld | [73] |
| Wessington Springs (2010) | 0.27 | (0.00-0.85) | Shoenfeld | [74] |
| Winnebago | 3.16 | (0.65-7.21) | Shoenfeld | [75] |
| ^a^ The Dillon Project was the only project in the Southwestern Biome represented by an available fatality report. Due to its singularity and since it is located very close to the Pacific Biome; it was combined with the Pacific Biome data. | | | | |

# References:

1. Young, D.P. Jr., M. Lout, Z. Courage, S. Nomani, and K. Bay. 2012. 2011 Post-Construction Monitoring Study, Criterion Wind Project, Garrett County, Maryland: April - November 2011. Revised November 25, 2013.

2. Young, D.P. Jr., Bay K, Nomani S, Tidhar W (2009) Nedpower Mount Storm Wind Energy Facility, Post-Construction Avian and Bat Monitoring: March - June 2009.

3. Young, D.P. Jr., Bay K, Nomani S, Tidhar W (2010) Nedpower Mount Storm Wind Energy Facility, Post-Construction Avian and Bat Monitoring: July - October 2009.

4. Young, D.P. Jr., Bay K, Nomani S, Tidhar W (2010) Nedpower Mount Storm Wind Energy Facility, Post-Construction Avian and Bat Monitoring: April - July 2010.

5. Young, D.P. Jr., Nomani S, Tidhar W, Bay K (2011) Nedpower Mount Storm Wind Energy Facility, Post-Construction Avian and Bat Monitoring: July - October 2010.

6. Young, D.P. Jr., Nomani S, Courage Z, Bay K (2011) Nedpower Mount Storm Wind Energy Facility, Post-Construction Avian and Bat Monitoring: April - July 2011.

7. Young, D.P. Jr., Nomani S, Courage Z, Bay K (2012) Nedpower Mount Storm Wind Energy Facility, Post-Construction Avian and Bat Monitoring: July - October 2011.

8. Jain A, Kerlinger P, Curry R, Slobodnik L, Quant J, et al. (2009) Annual Report for the Noble Bliss Windpark, LLC, Postconstruction Bird and Bat Fatality Study - 2008.

9. Jain A, Kerlinger P, Slobodnik L, Curry R, Fuerst A, et al. (2010) Annual Report for the Noble Bliss Windpark, LLC: Postconstruction Bird and Bat Fatality Study - 2009.

10. Jain A, Kerlinger P, Slobodnik L, Curry R, Harte A (2011) Annual Report for the Noble Wethersfield Windpark, LLC: Postconstruction Bird and Bat Fatality Study - 2010.

11. Jeffrey JD, Bay K, Erickson WP, Sonneberg M, Baker J, et al. (2009) Portland General Electric Biglow Canyon Wind Farm Phase I Post-Construction Avian and Bat Monitoring First Annual Report, Sherman County, Oregon. January 2008 - December 2008.

12. Enk T, Bay K, Sonnenberg M, Baker J, Kesterke M, et al. (2010) Biglow Canyon Wind Farm Phase I Post-Construction Avian and Bat Monitoring Second Annual Report, Sherman County, Oregon. January 26, 2009 - December 11, 2009.

13. Enk T, Bay K, Sonnenberg M, Flaig J, Boehrs JR, et al. (2011) Year 1 Post-Construction Avian and Bat Monitoring Report: Biglow Canyon Wind Farm Phase II, Sherman County, Oregon. September 10, 2009 - September 12, 2010.

14. Enk T, Bay K, Sonnenberg M, Boehrs JR (2012) Year 2 Avian and Bat Monitoring Report: Biglow Canyon Wind Farm Phase II, Sherman County, Oregon. September 13, 2010 - September 12, 2011.

15. Enk T, Bay K, Sonnenberg M, Boehrs JR (2012) Year 1 Avian and Bat Monitoring Report: Biglow Canyon Wind Farm Phase III, Sherman County, Oregon. September 13, 2010 - September 9, 2011.

16. Young, D.P. Jr., Jeffrey J, Erickson WP, Bay K, Poulton VK, et al. (2006) Eurus Combine Hills Turbine Ranch. Phase 1 Post Construction Wildlife Monitoring First Annual Report: February 2004 - February 2005.

17. Enz T, Bay K, Sonnenberg M, Palochak A (2012) Post-Construction Monitoring Studies for the Combine Hills Turbine Ranch, Umatilla County, Oregon. Final Report: January 7 - December 2, 2011.

18. Thompson J, Solick D, Bay K (2011) Post-Construction Fatality Surveys for the Dry Lake Phase I Wind Project. Iberdrola Renewables: September 2009 - November 2010.

19. Thompson J, Bay K (2012) Post-Construction Fatality Surveys for the Dry Lake II Wind Project: February 2011 – February 2012.

20. Jeffrey JD, Erickson WP, Bay K, Sonneberg M, Baker J, et al. (2009) Horizon Wind Energy, Elkhorn Valley Wind Project, Post-Construction Avian and Bat Monitoring, First Annual Report, January-December 2008.

21. Enk T, Derby C, Bay K, Sonnenberg M (2011) 2010 Post-Construction Fatality Monitoring Report, Elkhorn Valley Wind Farm, Union County, Oregon. January – December 2010.

22. Young, D.P. Jr., Erickson WP, Good RE, Strickland MD, Johnson GD (2003) Avian and Bat Mortality Associated with the Initial Phase of the Foote Creek Rim Windpower Project, Carbon County, Wyoming, Final Report, November 1998 - June 2002.

23. URS Corporation (2010) Final Goodnoe Hills Wind Project Avian Mortality Monitoring Report.

24. Gritski R, Kronner K (2010) Hay Canyon Wind Power Project Wildlife Monitoring Study: May 2009 - May 2010.

25. Young, D.P. Jr., Erickson WP, Jeffrey J, Poulton VK (2007) Puget Sound Energy Hopkins Ridge Wind Project Phase 1 Post-Construction Avian and Bat Monitoring First Annual Report, January - December 2006. 25 pp. p.

26. Young, D.P. Jr., Jeffrey JD, Bay K, Erickson WP (2009) Puget Sound Energy Hopkins Ridge Wind Project, Phase 1, Columbia County, Washington. Post-Construction Avian and Bat Monitoring, Second Annual Report: January - December, 2008.

27. Stantec Consulting Services, Inc. (Stantec Consulting Services) (2012) Post-Construction Monitoring, Summer 2011 - Spring 2012, Year 1 Annual Report: Kittitas Valley Wind Power Project, Cle Elum, Washington.

28. Johnson GD, Erickson WP, White J (2003) Avian and Bat Mortality During the First Year of Operation at the Klondike Phase I Wind Project, Sherman County, Oregon.

29. Northwest Wildlife Consultants, Inc. (NWC), Western EcoSystems Technology, Inc. (WEST) (2007) Avian and Bat Monitoring Report for the Klondike II Wind Power Project. Sherman County, Oregon.

30. Gritski R, Downes S, Kronner K (2010) Klondike III (Phase 1) Wind Power Project Wildlife Monitoring: October 2007-October 2009.

31. Gritski R, Downes S, Kronner K (2011) Klondike IIIa (Phase 2) Wind Power Project Wildlife Monitoring: August 2008 - August 2010.

32. Enz T, Bay K (2011) Post-Construction Monitoring at the Linden Ranch Wind Farm, Klickitat County, Washington. Final Report: June 30, 2010 - July 17, 2011.

33. URS Corporation (2010) Final Marengo I Wind Project Year One Avian Mortality Monitoring Report.

34. URS Corporation (2010) Final Marengo II Wind Project Year One Avian Mortality Monitoring Report.

35. Erickson WP, Kronner K, Gritski R (2003) Nine Canyon Wind Power Project Avian and Bat Monitoring Report. September 2002 – August 2003.

36. Erickson WP, Jeffrey J, Kronner K, Bay K (2004) Stateline Wind Project Wildlife Monitoring Annual Report. July 2001 - December 2003.

37. Erickson WP, Kronner K, Bay KJ (2007) Stateline 2 Wind Project Wildlife Monitoring Report, January - December 2006.

38. Erickson WP, Johnson GD, Strickland MD, Kronner K (2000) Avian and Bat Mortality Associated with the Vansycle Wind Project, Umatilla County, Oregon: 1999 Study Year.

39. Ventus Environmental Solutions (Ventus) (2012) Vantage Wind Energy Center Avian and Bat Monitoring Study: March 2011- March 2012.

40. Erickson WP, Jeffrey J, Poulton VK (2008) Avian and Bat Monitoring: Year 1 Report. Puget Sound Energy Wild Horse Wind Project, Kittitas County, Washington.

41. Enz T, Bay K, Nomani S, Kesterke M (2011) Bird and Bat Fatality Monitoring Study, Windy Flats and Windy Point II Wind Energy Projects, Klickitat County, Washington. Final Report: February 1, 2010 - January 14, 2011.

42. Tidhar D, Tidhar WL, McManus L, Courage Z (2011) 2010 Post-Construction Fatality Surveys for the Lempster Wind Project, Lempster, New Hampshire.

43. Jain A, Kerlinger P, Curry R, Slobodnik L (2009) Annual Report for the Maple Ridge Wind Power Project: Post-Construction Bird and Bat Fatality Study - 2007.

44. Jain A, Kerlinger P, Curry R, Slobodnik L, Lehman M (2009) Maple Ridge Wind Power Avian and Bat Fatality Study Report - 2008.

45. Jain A, Kerlinger P, Slobodnik L, Curry R, Russell K (2011) Annual Report for the Noble Altona Windpark, LLC: Postconstruction Bird and Bat Fatality Study - 2010.

46. Jain A, Kerlinger P, Slobodnik L, Curry R, Russell K (2011) Annual Report for the Noble Chateaugay Windpark, LLC: Postconstruction Bird and Bat Fatality Study - 2010.

47. Jain A, Kerlinger P, Curry R, Slobodnik L, Histed J, et al. (2009) Annual Report for the Noble Clinton Windpark, LLC, Postconstruction Bird and Bat Fatality Study - 2008.

48. Jain A, Kerlinger P, Slobodnik L, Curry R, Russell K (2010) Annual Report for the Noble Clinton Windpark, LLC: Postconstruction Bird and Bat Fatality Study - 2009.

49. Jain A, Kerlinger P, Curry R, Slobodnik L, Fuerst A, et al. (2009) Annual Report for the Noble Ellenburg Windpark, LLC, Postconstruction Bird and Bat Fatality Study - 2008.

50. Jain A, Kerlinger P, Slobodnik L, Curry R, Russell K (2010) Annual Report for the Noble Ellenburg Windpark, LLC: Postconstruction Bird and Bat Fatality Study - 2009.

51. Chatfield A, Erickson WP, Bay K (2010) Final Report: Avian and Bat Fatality Study at the Alite Wind-Energy Facility, Kern County, California. Final Report: June 15, 2009 – June 15, 2010.

52. Western EcoSystems Technology, Inc. (WEST) (2006) Diablo Winds Wildlife Monitoring Progress Report, March 2005 - February 2006. Cheyenne, Wyoming: WEST.

53. Western EcoSystems Technology, Inc. (WEST) (2008) Diablo Winds Wildlife Monitoring Progress Report: March 2005 – February 2007.

54. Chatfield A, Erickson W, Bay K (2009) Avian and Bat Fatality Study, Dillon Wind-Energy Facility, Riverside County, California. Final Report: March 26, 2008 - March 26, 2009.

55. BioResource Consultants, Inc. (BRC) (2010) 2009/2010 Annual Report: Bird and Bat Mortality Monitoring, Pine Tree Wind Farm, Kern County, California.

56. Kerlinger P, Curry R, Culp L, Hasch A, Jain A (2009) Post-Construction Avian Monitoring Study for the Shiloh I Wind Power Project, Solano County, California. Final Report: October 2009.

57. Kerlinger P, Curry R, Culp L, Hasch A, Jain A (2010) Post-Construction Avian Monitoring Study for the Shiloh II Wind Power Project, Solano County, California. Year One Report.

58. Western EcoSystems Technology, Inc. (WEST) (2011) Post-Construction Fatality Surveys for the Barton Chapel Wind Project: Iberdrola Renewables. Version: July 2011.

59. Gruver J, Sonnenberg M, Bay K, Erickson W (2009) Post-Construction Bat and Bird Fatality Study at the Blue Sky Green Field Wind Energy Center, Fond Du Lac County, Wisconsin July 21 - October 31, 2008 and March 15 - June 4, 2009.

60. Tierney R (2007) Buffalo Gap I Wind Farm Avian Mortality Study: February 2006-January 2007.

61. Tierney R (2009) Buffalo Gap 2 Wind Farm Avian Mortality Study: July 2007 - December 2008. Final Survey Report.

62. Johnson GD, Erickson WP, Strickland MD, Shepherd MF, Shepherd DA (2000) Avian Monitoring Studies at the Buffalo Ridge Wind Resource Area, Minnesota: Results of a 4-Year Study. 212 pp.

63. Derby C, Chodachek K, Bay K, Merrill A (2010) Post-Construction Fatality Survey for the Buffalo Ridge I Wind Project. May 2009 - May 2010.

64. Derby C, Chodachek K, Sonnenberg M (2012) Post-Construction Casualty Surveys for the Buffalo Ridge II Wind Project. Iberdrola Renewables: March 2011- February 2012.

65. BHE Environmental, Inc. (BHE) (2010) Post-Construction Bird and Bat Mortality Study: Cedar Ridge Wind Farm, Fond Du Lac County, Wisconsin.

66. BHE Environmental, Inc. (BHE) (2011) Post-Construction Bird and Bat Mortality Study: Cedar Ridge Wind Farm, Fond Du Lac County, Wisconsin.

67. Derby C, Chodachek K, Bay K, Merrill A (2010) Post-Construction Fatality Surveys for the Elm Creek Wind Project: March 2009- February 2010.

68. Derby C, Chodachek K, Sonnenberg M (2012) Post-Construction Fatality Surveys for the Elm Creek II Wind Project. Iberdrola Renewables: March 2011-February 2012.

69. Derby C, Ritzert J, Bay K (2010) Bird and Bat Fatality Study, Grand Ridge Wind Resource Area, Lasalle County, Illinois. January 2009 - January 2010.

70. Derby C, Dahl A, Erickson W, Bay K, Hoban J (2007) Post-Construction Monitoring Report for Avian and Bat Mortality at the NPPD Ainsworth Wind Farm. Unpublished report prepared by Western EcoSystems Technology, Inc. (WEST), Cheyenne, Wyoming, for the Nebraska Public Power District.

71. Derby C, Dahl A, Merrill A (2012) Post-Construction Monitoring Results for the PrairieWinds SD1 Wind Energy Facility, South Dakota. Final Report: March 2011 - February 2012.

72. Derby C, Chodachek K, Bay K, Nomani S (2011) Post-Construction Fatality Surveys for the Rugby Wind Project: Iberdrola Renewables, Inc. March 2010 - March 2011.

73. Derby C, Dahl A, Merrill A, Bay K (2010) 2009 Post-Construction Monitoring Results for the Wessington Springs Wind-Energy Facility, South Dakota. Final Report.

74. Derby C, Dahl A, Bay K, McManus L (2011) 2010 Post-Construction Monitoring Results for the Wessington Springs Wind Energy Facility, South Dakota. Final Report: March 9 – November 16, 2010.

75. Derby C, Chodachek K, Bay K, Merrill A (2010) Post-Construction Fatality Surveys for the Winnebago Wind Project: March 2009- February 2010.
