# Supplementary material for: A Comprehensive Analysis of Small-Passerine Fatalities from Collision with Turbines at Wind Energy Facilities
Source: PLoS One. 2014 Sep 15;9(9):e107491. doi: 10.1371/journal.pone.0107491 (PMC4164633; doi:10.1371/journal.pone.0107491)
Supplement: Appendix S3 — Variables used to calculate a multiplier value used to determine estimates of small-bird rates of fatality for wind energy fatality studies providing only all-bird estimates, in the United States and Canada, grouped by avifaunal biome. (DOCX) [file pone.0107491.s030.docx]

**Appendix S3. Variables used to calculate a multiplier value used to determine estimates of small-bird rates of fatality for wind energy fatality studies providing only all-bird estimates, in the United States and Canada, grouped by avifaunal biome.**

|  | **Average Removal Time (ART; days)** | **Searcher Efficiency Rate (SEEF)** | **Average Search Interval (I; days)** | **Probability of Availability and Detection (pi-hat)** |
| --- | --- | --- | --- | --- |
| **Eastern Biome** | | | | |
| **Large Birds** |  |  |  |  |
| Daily | 5.33 | 0.89 | 1 | 0.89 |
| Bi-weekly | 5.33 | 0.89 | 3.5 | 0.69 |
| Weekly | 5.33 | 0.89 | 7 | 0.51 |
| Bi-monthly | 5.33 | 0.89 | 14 | 0.32 |
| Monthly | 5.33 | 0.89 | 28 | 0.17 |
| **Small Birds** |  |  |  |  |
| Daily | 4.51 | 0.50 | 1 | 0.75 |
| Bi-weekly | 4.51 | 0.50 | 3.5 | 0.45 |
| Weekly | 4.51 | 0.50 | 7 | 0.29 |
| Bi-monthly | 4.51 | 0.50 | 14 | 0.16 |
| Monthly | 4.51 | 0.50 | 28 | 0.08 |
| **Intermountain West Biome** | | | | |
| **Large Birds** |  |  |  |  |
| Daily | 24.52 | 0.72 | 1 | 0.97 |
| Bi-weekly | 24.52 | 0.72 | 3.5 | 0.89 |
| Weekly | 24.52 | 0.72 | 7 | 0.79 |
| Bi-monthly | 24.52 | 0.72 | 14 | 0.65 |
| Monthly | 24.52 | 0.72 | 28 | 0.47 |
| **Small Birds** |  |  |  |  |
| Daily | 14.64 | 0.48 | 1 | 0.90 |
| Bi-weekly | 14.64 | 0.48 | 3.5 | 0.72 |
| Weekly | 14.64 | 0.48 | 7 | 0.56 |
| Bi-monthly | 14.64 | 0.48 | 14 | 0.38 |
| Monthly | 14.64 | 0.48 | 28 | 0.23 |
| **Northern Forest Biome** | | | | |
| **Large Birds** |  |  |  |  |
| Daily | 8.62 | 0.86 | 1 | 0.93 |
| Bi-weekly | 8.62 | 0.86 | 3.5 | 0.78 |
| Weekly | 8.62 | 0.86 | 7 | 0.63 |
| Bi-monthly | 8.62 | 0.86 | 14 | 0.44 |
| Monthly | 8.62 | 0.86 | 28 | 0.25 |
| **Small Birds** |  |  |  |  |
| Daily | 5.62 | 0.65 | 1 | 0.84 |
| Bi-weekly | 5.62 | 0.65 | 3.5 | 0.60 |
| Weekly | 5.62 | 0.65 | 7 | 0.41 |
| Bi-monthly | 5.62 | 0.65 | 14 | 0.25 |
| Monthly | 5.62 | 0.65 | 28 | 0.13 |
| **Pacific Biome** | | | | |
| **Large Birds** |  |  |  |  |
| Daily | 22.09 | 0.85 | 1 | 0.97 |
| Bi-weekly | 22.09 | 0.85 | 3.5 | 0.90 |
| Weekly | 22.09 | 0.85 | 7 | 0.82 |
| Bi-monthly | 22.09 | 0.85 | 14 | 0.68 |
| Monthly | 22.09 | 0.85 | 28 | 0.50 |
| **Small Birds** |  |  |  |  |
| Daily | 8.39 | 0.52 | 1 | 0.85 |
| Bi-weekly | 8.39 | 0.52 | 3.5 | 0.62 |
| Weekly | 8.39 | 0.52 | 7 | 0.44 |
| Bi-monthly | 8.39 | 0.52 | 14 | 0.28 |
| Monthly | 8.39 | 0.52 | 28 | 0.15 |
| **Prairie Biome** | | | | |
| **Large Birds** |  |  |  |  |
| Daily | 19.94 | 0.78 | 1 | 0.96 |
| Bi-weekly | 19.94 | 0.78 | 3.5 | 0.88 |
| Weekly | 19.94 | 0.78 | 7 | 0.78 |
| Bi-monthly | 19.94 | 0.78 | 14 | 0.63 |
| Monthly | 19.94 | 0.78 | 28 | 0.44 |
| **Small Birds** |  |  |  |  |
| Daily | 7.69 | 0.58 | 1 | 0.86 |
| Bi-weekly | 7.69 | 0.58 | 3.5 | 0.64 |
| Weekly | 7.69 | 0.58 | 7 | 0.46 |
| Bi-monthly | 7.69 | 0.58 | 14 | 0.29 |
| Monthly | 7.69 | 0.58 | 28 | 0.16 |
