# Supplementary material for: A Comprehensive Analysis of Small-Passerine Fatalities from Collision with Turbines at Wind Energy Facilities
Source: PLoS One. 2014 Sep 15;9(9):e107491. doi: 10.1371/journal.pone.0107491 (PMC4164633; doi:10.1371/journal.pone.0107491)
Supplement: Appendix S5 — Additional variables used to calculate a multiplier value used to determine estimated rates of fatality for small birds and the calculated multiplier for wind energy fatality studies providing only all-bird estimates for each associated avifaunal biome in the United States and Canada. See equation in methods section of main document. (DOCX) [file pone.0107491.s032.docx]

**Appendix S5. Additional variables used to calculate a multiplier value used to determine estimated rates of fatality for small birds and the calculated multiplier for wind energy fatality studies providing only all-bird estimates for each associated avifaunal biome in the United States and Canada.** See equation in methods section of main document.

| **Search Interval** | **Ratio of Probability of Availability and Detection of Large Birds Over Small Birds (pi LB/pi SB)** | **Proportion Large Birds** | **Proportion Small Birds** | **Multiplier** |
| --- | --- | --- | --- | --- |
| **Eastern Biome** | | | | |
| Daily | 1.19 | 0.19 | 0.81 | 0.84 |
| Bi-weekly | 1.53 | 0.19 | 0.81 | 0.87 |
| Weekly | 1.79 | 0.19 | 0.81 | 0.88 |
| Bi-monthly | 2.01 | 0.19 | 0.81 | 0.90 |
| Monthly | 2.09 | 0.19 | 0.81 | 0.90 |
|  |  |  | **Average** | **0.88** |
| **Intermountain West Biome** | | | | |
| Daily | 1.07 | 0.29 | 0.71 | 0.72 |
| Bi-weekly | 1.23 | 0.29 | 0.71 | 0.75 |
| Weekly | 1.42 | 0.29 | 0.71 | 0.77 |
| Bi-monthly | 1.70 | 0.29 | 0.71 | 0.80 |
| Monthly | 2.06 | 0.29 | 0.71 | 0.83 |
|  |  |  | **Average** | **0.78** |
| **Northern Forest Biome** | | | | |
| Daily | 1.10 | 0.26 | 0.74 | 0.76 |
| Bi-weekly | 1.30 | 0.26 | 0.74 | 0.79 |
| Weekly | 1.51 | 0.26 | 0.74 | 0.81 |
| Bi-monthly | 1.76 | 0.26 | 0.74 | 0.84 |
| Monthly | 1.95 | 0.26 | 0.74 | 0.85 |
|  |  |  | **Average** | **0.81** |
| **Pacific Biome** | | | | |
| Daily | 1.14 | 0.48 | 0.52 | 0.55 |
| Bi-weekly | 1.45 | 0.48 | 0.52 | 0.61 |
| Weekly | 1.84 | 0.48 | 0.52 | 0.66 |
| Bi-monthly | 2.47 | 0.48 | 0.52 | 0.73 |
| Monthly | 3.31 | 0.48 | 0.52 | 0.78 |
|  |  |  | **Average** | **0.67** |
| **Prairie Biome** | | | | |
| Daily | 1.11 | 0.45 | 0.55 | 0.58 |
| Bi-weekly | 1.38 | 0.45 | 0.55 | 0.63 |
| Weekly | 1.69 | 0.45 | 0.55 | 0.68 |
| Bi-monthly | 2.18 | 0.45 | 0.55 | 0.73 |
| Monthly | 2.80 | 0.45 | 0.55 | 0.78 |
|  |  |  | **Average** | **0.68** |
