# Supplementary material for: A Comprehensive Analysis of Small-Passerine Fatalities from Collision with Turbines at Wind Energy Facilities
Source: PLoS One. 2014 Sep 15;9(9):e107491. doi: 10.1371/journal.pone.0107491 (PMC4164633; doi:10.1371/journal.pone.0107491)
Supplement: Appendix S7 — Fatality studies conducted at wind energy facilities in the United States and Canada, sorted by avifaunal biome region, with associated small-bird fatality rate estimates (birds/megawatt/year), for which the estimator type is identified, the applied low and high bias adjustment factors, followed by the resulting low and high bias-adjusted fatality rates for small birds. (DOCX) [file pone.0107491.s034.docx]

**Appendix S7. Fatality studies conducted at wind energy facilities in the United States and Canada, sorted by avifaunal biome region, with associated small-bird fatality rate estimates (birds/megawatt /year), for which the estimator type is identified, the applied low and high bias adjustment factors, followed by the resulting low and high bias-adjusted fatality rates for small birds.**

| **Project name** | **Estimator used** | **Small bird est^a^** | **Low adjustment** | **High adjustment** | **Adj est (low bias) ^b^** | **Adj est (high bias) ^c^** | **Reference** |
| --- | --- | --- | --- | --- | --- | --- | --- |
| **Eastern Biome** | | | | | | | |
| Buffalo Mountain (2000-2003) | Naïve | 9.65 | 0.52 | 0.70 | 18.54 | 13.81 | [1] |
| Buffalo Mountain (2005) | Naïve | 0.98 | 0.52 | 0.70 | 1.88 | 1.40 | [2] |
| Casselman (2008) | Shoenfeld | 1.33 | 0.63 | 0.81 | 2.11 | 1.64 | [3] |
| Casselman (2009) | Shoenfeld | 2.52 | 0.63 | 0.81 | 4.02 | 3.12 | [4] |
| Cohocton/Dutch Hill (2009) | Jain | 1.20 | 1.26 | 1.74 | 0.96 | 0.69 | [5] |
| Cohocton/Dutch Hills (2010) | Jain | 0.69 | 1.26 | 1.74 | 0.55 | 0.40 | [6] |
| Criterion (2011) | Schoenfeld | 6.10 | 0.59 | 0.85 | 10.27 | 7.14 | [7] |
| Locust Ridge II (2009) | Shoenfeld | 0.74 | 0.39 | 0.73 | 1.89 | 1.01 | [8] |
| Locust Ridge II (2010) | Shoenfeld | 0.66 | 0.90 | 0.96 | 0.74 | 0.69 | [8] |
| Mount Storm (2009) | Shoenfeld | 3.10 | 0.39 | 1.01 | 7.95 | 3.07 | [9, 10] |
| Mount Storm (2010) | Shoenfeld | 2.19 | 0.39 | 0.73 | 5.62 | 3.02 | [11, 12] |
| Mount Storm (2011) | Shoenfeld | 3.93 | 0.63 | 0.81 | 6.26 | 4.86 | [13, 14] |
| Mountaineer (2003) | Shoenfeld | 2.36 | 0.55 | 1.08 | 4.30 | 2.18 | [15] |
| Munnsville (2008) | Jain | 1.30 | 1.07 | 1.33 | 1.21 | 0.98 | [16] |
| Noble Bliss (2008) | Jain | 1.09 | 1.26 | 1.74 | 0.87 | 0.63 | [17] |
| Noble Bliss (2009) | Jain | 1.82 | 1.44 | 2.25 | 1.26 | 0.81 | [18] |
| Noble Wethersfield (2010) | Jain | 1.51 | 1.26 | 1.33 | 1.20 | 1.14 | [19] |
| Ripley (2008) | Jain | 2.70 | 1.26 | 1.33 | 2.15 | 2.03 | [20] |
| Sheldon (2010) | Shoenfeld | 1.54 | 0.60 | 0.96 | 2.59 | 1.61 | [21] |
| Sheldon (2011) | Shoenfeld | 1.38 | 0.60 | 0.96 | 2.32 | 1.44 | [22] |
| **Intermountain West Biome** | | | | | | | |
| Big Horn | Huso | 1.97 | 0.72 | 1.15 | 2.72 | 1.72 | [23] |
| Biglow Canyon (Phase I; 2008) | Shoenfeld | 1.54 | 0.49 | 0.95 | 3.17 | 1.62 | [24] |
| Biglow Canyon (Phase I; 2009) | Shoenfeld | 2.33 | 0.57 | 0.85 | 4.06 | 2.73 | [25] |
| Biglow Canyon (Phase II; 2009/2010) | Shoenfeld | 5.16 | 0.60 | 1.20 | 8.53 | 4.30 | [26] |
| Biglow Canyon (Phase II; 2010/2011) | Shoenfeld | 2.48 | 0.65 | 0.95 | 3.83 | 2.61 | [27] |
| Biglow Canyon (Phase III; 2010/2011) | Shoenfeld | 2.17 | 0.65 | 0.95 | 3.35 | 2.28 | [28] |
| Combine Hills (2004/2005) | Shoenfeld | 1.89 | 0.71 | 0.85 | 2.65 | 2.22 | [29] |
| Combine Hills (2011) | Shoenfeld | 1.96 | 0.71 | 0.85 | 2.75 | 2.30 | [30] |
| Dry Lake I | Shoenfeld | 1.93 | 0.64 | 0.96 | 3.00 | 2.00 | [31] |
| Dry Lake II | Shoenfeld | 1.30 | 0.60 | 0.67 | 2.18 | 1.94 | [32] |
| Elkhorn (2008) | Shoenfeld | 0.45 | 0.69 | 0.84 | 0.65 | 0.54 | [33] |
| Elkhorn (2010) | Shoenfeld | 1.76 | 0.62 | 1.08 | 2.82 | 1.62 | [34] |
| Foote Creek Rim (Phase I; 1999) | Shoenfeld | 3.00 | 0.71 | 0.85 | 4.21 | 3.52 | [35] |
| Foote Creek Rim (Phase I; 2000) | Shoenfeld | 2.15 | 0.71 | 0.85 | 3.02 | 2.52 | [35] |
| Foote Creek Rim (Phase I; 2001-2002) | Shoenfeld | 1.71 | 0.71 | 0.85 | 2.40 | 2.01 | [35] |
| Goodnoe | Shoenfeld | 1.02 | 0.66 | 0.82 | 1.54 | 1.24 | [36] |
| Harvest Wind (2010-2012) | Huso | 2.28 | 0.82 | 1.67 | 2.80 | 1.37 | [37] |
| Hay Canyon | Huso | 1.92 | 0.72 | 1.15 | 2.65 | 1.68 | [38] |
| Hopkins Ridge (2006) | Shoenfeld | 0.80 | 0.56 | 0.84 | 1.42 | 0.95 | [39] |
| Hopkins Ridge (2008) | Shoenfeld | 2.43 | 0.49 | 0.95 | 5.01 | 2.56 | [40] |
| Kittitas Valley (2011-2012) | Huso | 0.72 | 0.97 | 1.87 | 0.74 | 0.39 | [41] |
| Klondike | Shoenfeld | 0.77 | 0.80 | 0.82 | 0.97 | 0.94 | [42] |
| Klondike II | Shoenfeld | 2.90 | 0.57 | 0.85 | 5.06 | 3.40 | [43] |
| Klondike III (Phase I) | Shoenfeld | 2.60 | 0.62 | 1.08 | 4.16 | 2.40 | [44] |
| Klondike IIIa (Phase II) | Shoenfeld | 1.92 | 0.62 | 1.08 | 3.07 | 1.77 | [45] |
| Leaning Juniper | Huso | 5.17 | 0.72 | 1.15 | 7.13 | 4.51 | [46] |
| Linden Ranch | Shoenfeld | 4.83 | 0.65 | 0.95 | 7.46 | 5.08 | [47] |
| Marengo I (2009) | Shoenfeld | 0.23 | 0.57 | 0.85 | 0.40 | 0.27 | [48] |
| Marengo II (2009) | Shoenfeld | 0.16 | 0.57 | 0.85 | 0.28 | 0.19 | [49] |
| Nine Canyon | Shoenfeld | 2.55 | 0.57 | 0.85 | 4.45 | 2.99 | [50] |
| Pebble Springs | Huso | 1.50 | 0.80 | 1.13 | 1.87 | 1.33 | [51] |
| Stateline (2002) | Shoenfeld | 2.85 | 0.57 | 0.85 | 4.97 | 3.34 | [52] |
| Stateline (2003) | Shoenfeld | 2.29 | 0.57 | 0.85 | 3.99 | 2.69 | [52] |
| Stateline (2006) | Shoenfeld | 0.95 | 0.52 | 0.85 | 1.81 | 1.11 | [53] |
| Summerview (2006) ^d^ |  | 0.82 |  |  |  |  | [54] |
| Tuolumne (Windy Point I) | Shoenfeld | 2.49 | 0.41 | 0.95 | 6.13 | 2.62 | [55] |
| Vansycle | Naïve | 0.75 | 0.57 | 0.70 | 1.31 | 1.07 | [56] |
| Vantage | Shoenfeld | 1.14 | 0.41 | 0.95 | 2.81 | 1.20 | [57] |
| White Creek (2007-2011) | Huso | 3.14 | 0.87 | 2.77 | 3.62 | 1.13 | [58] |
| Wild Horse | Shoenfeld | 1.28 | 0.52 | 0.85 | 2.44 | 1.50 | [59] |
| Windy Flats (Windy Point II) | Shoenfeld | 7.59 | 0.87 | 1.86 | 8.75 | 4.09 | [60] |
| **Northern Forest Biome** | | | | | | | |
| Lempster (2009) | Shoenfeld | 2.73 | 0.63 | 0.81 | 4.35 | 3.38 | [61] |
| Lempster (2010) | Shoenfeld | 1.65 | 0.55 | 0.78 | 3.00 | 2.11 | [62] |
| Maple Ridge (2007) | Jain | 2.73 | 1.26 | 1.33 | 2.17 | 2.05 | [63] |
| Maple Ridge (2008) | Jain | 1.86 | 1.44 | 1.47 | 1.29 | 1.26 | [64] |
| Mars Hill (2007) | Jain | 1.33 | 1.05 | 1.33 | 1.27 | 1.00 | [65] |
| Mars Hill (2008) | Jain | 1.43 | 1.07 | 1.33 | 1.33 | 1.08 | [66] |
| Noble Altona (2010) | Jain | 1.68 | 1.44 | 2.25 | 1.17 | 0.75 | [67] |
| Noble Chateaugay (2010) | Jain | 1.01 | 1.44 | 1.47 | 0.70 | 0.69 | [68] |
| Noble Clinton (2008) | Jain | 1.39 | 1.44 | 2.25 | 0.97 | 0.62 | [69] |
| Noble Clinton (2009) | Jain | 0.57 | 1.44 | 2.25 | 0.40 | 0.25 | [70] |
| Noble Ellenburg (2008) | Jain | 0.66 | 1.44 | 2.25 | 0.46 | 0.29 | [71] |
| Noble Ellenburg (2009) | Jain | 2.02 | 1.44 | 2.25 | 1.40 | 0.90 | [72] |
| Stetson Mountain I (2009) | Jain | 2.17 | 1.44 | 1.47 | 1.51 | 1.48 | [73] |
| Stetson Mountain I (2011) | Jain | 0.96 | 1.44 | 1.47 | 0.66 | 0.65 | [74] |
| Stetson Mountain II (2010) | Jain | 1.15 | 1.44 | 1.47 | 0.80 | 0.78 | [75] |
| **Pacific Biome** | | | | | | | |
| Alite (2010) | Shoenfeld | 0.24 | 0.55 | 0.88 | 0.44 | 0.27 | [76] |
| Diablo Winds | Shoenfeld | 3.67 | 0.92 | 1.08 | 4.01 | 3.38 | [77] |
| Dillon ^e^ | Shoenfeld | 4.26 | 0.63 | 0.75 | 6.71 | 5.71 | [77, 78] |
| High Winds (2004) | Shoenfeld | 1.08 | 0.62 | 0.88 | 1.73 | 1.23 | [79] |
| High Winds (2005) | Shoenfeld | 0.73 | 0.62 | 0.88 | 1.17 | 0.83 | [80] |
| Pine Tree | Shoenfeld | 3.87 | 0.55 | 0.81 | 7.04 | 4.79 | [81] |
| Shiloh I | Jain | 4.86 | 1.07 | 1.33 | 4.53 | 3.66 | [82] |
| Shiloh II (Year 1) | Jain | 0.81 | 1.44 | 1.47 | 0.56 | 0.55 | [83] |
| **Prairie Biome** | | | | | | | |
| Barton Chapel | Shoenfeld | 0.62 | 0.55 | 1.08 | 1.13 | 0.57 | [84] |
| Barton I and II | Shoenfeld | 3.73 | 0.60 | 0.96 | 6.27 | 3.87 | [85] |
| BSGF | Shoenfeld | 6.96 | 0.52 | 0.85 | 13.26 | 8.15 | [86] |
| Buffalo Gap I | Shoenfeld | 1.08 | 0.62 | 1.08 | 1.73 | 1.00 | [87] |
| Buffalo Gap II | Shoenfeld | 0.07 | 0.62 | 1.08 | 0.11 | 0.06 | [88] |
| Buffalo Ridge (Phase I; 1996) | Naïve | 4.14 | 0.50 | 1.30 | 8.22 | 3.19 | [89] |
| Buffalo Ridge (Phase I; 1997) | Naïve | 1.40 | 0.50 | 1.30 | 2.78 | 1.08 | [89] |
| Buffalo Ridge (Phase I; 1998) | Naïve | 2.40 | 0.50 | 1.30 | 4.76 | 1.85 | [89] |
| Buffalo Ridge (Phase I; 1999) | Naïve | 1.43 | 0.50 | 1.30 | 2.83 | 1.10 | [89] |
| Buffalo Ridge (Phase II; 1998) | Naïve | 2.28 | 0.50 | 1.30 | 4.52 | 1.76 | [89] |
| Buffalo Ridge (Phase II; 1999) | Naïve | 3.04 | 0.50 | 1.30 | 6.03 | 2.34 | [89] |
| Buffalo Ridge (Phase III; 1999) | Naïve | 5.93 | 0.50 | 1.30 | 11.76 | 4.57 | [89] |
| Buffalo Ridge I (2010) | Shoenfeld | 4.16 | 0.55 | 1.08 | 7.57 | 3.84 | [90] |
| Buffalo Ridge II (2011) | Shoenfeld | 1.12 | 0.60 | 0.96 | 1.88 | 1.16 | [91] |
| Cedar Ridge (2009) | Huso | 5.21 | 1.11 | 1.47 | 4.68 | 3.53 | [92] |
| Cedar Ridge (2010) | Shoenfeld | 2.21 | 0.90 | 0.96 | 2.46 | 2.31 | [93] |
| Elm Creek | Shoenfeld | 1.07 | 0.60 | 0.96 | 1.80 | 1.11 | [94] |
| Elm Creek II | Shoenfeld | 3.60 | 0.60 | 0.96 | 6.05 | 3.73 | [95] |
| Grand Ridge I | Shoenfeld | 0 | 0.55 | 1.08 | 0 | 0 | [96] |
| Kewaunee County | Shoenfeld | 1.33 | 0.60 | 0.96 | 2.23 | 1.38 | [97] |
| Moraine II | Shoenfeld | 3.79 | 0.60 | 0.96 | 6.37 | 3.93 | [98] |
| NPPD Ainsworth | Shoenfeld | 1.51 | 0.62 | 0.88 | 2.42 | 1.72 | [99] |
| Pioneer Prairie I (Phase II) | Shoenfeld | 0.18 | 0.60 | 0.96 | 0.31 | 0.19 | [100] |
| Prairie Winds SD1 (Crow Lake) | Shoenfeld | 0.96 | 0.57 | 0.85 | 1.67 | 1.13 | [101] |
| Prairie Winds ND1/Minot (2010) | Shoenfeld | 1.04 | 0.57 | 0.81 | 1.82 | 1.29 | [102] |
| Prairie Winds ND1/Minot (2011) | Shoenfeld | 0.96 | 0.49 | 0.86 | 1.98 | 1.12 | [103] |
| Rugby | Shoenfeld | 1.03 | 0.55 | 1.08 | 1.87 | 0.95 | [104] |
| Top of Iowa (2003) | Shoenfeld | 0.29 | 0.55 | 0.81 | 0.52 | 0.35 | [105] |
| Top of Iowa (2004) | Shoenfeld | 0.57 | 0.55 | 0.81 | 1.04 | 0.71 | [105] |
| Wessington Springs (2009) | Shoenfeld | 7.67 | 0.60 | 1.08 | 12.68 | 7.10 | [106] |
| Wessington Springs (2010) | Shoenfeld | 0.27 | 0.52 | 0.85 | 0.51 | 0.32 | [107] |
| Winnebago | Shoenfeld | 3.16 | 0.60 | 0.96 | 5.31 | 3.28 | [108] |
| ^a^  Fatality estimate for small birds(MW/year)  ^b^ Average estimate (MW/year) using the lowest bias adjustment  ^c^ Average estimate (MW/year) using the highest bias adjustment  ^d^ The Summerview (2006) fatality estimate for small birds was not adjusted, as it was calculated using a unique estimator from Environment Canada.  ^e^ The Dillon Project was the only project in the Southwestern Biome represented by an available fatality report. Due to its singularity and since it is located very close to the Pacific Biome; it was combined with the Pacific Biome data. | | | | | | | |

**References:**

1. Nicholson CP, R.D. Tankersley J, Fiedler JK, Nicholas NS (2005) Assessment and Prediction of Bird and Bat Mortality at Wind Energy Facilities in the Southeastern United States.

2. Fiedler JK, Henry TH, Tankersley RD, Nicholson CP (2007) Results of Bat and Bird Mortality Monitoring at the Expanded Buffalo Mountain Windfarm, 2005.

3. Arnett EB, Schirmacher MR, Huso MMP, Hayes JP (2009) Effectiveness of Changing Wind Turbine Cut-in Speed to Reduce Bat Fatalities at Wind Facilities: 2008 Annual Report.

4. Arnett EB, Schirmacher MR, Huso MMP, Hayes JP (2010) Patterns of Bat Fatality at the Casselman Wind Project in South-Central Pennsylvania.

5. Stantec Consulting, Inc. (Stantec) (2010) Cohocton and Dutch Hill Wind Farms Year 1 Post-Construction Monitoring Report, 2009, for the Cohocton and Dutch Hill Wind Farms in Cohocton, New York.

6. Stantec Consulting, Inc. (Stantec) (2011) Cohocton and Dutch Hill Wind Farms Year 2 Post-Construction Monitoring Report, 2010, for the Cohocton and Dutch Hill Wind Farms in Cohocton, New York.

7. Young, D.P. Jr., M. Lout, Z. Courage, S. Nomani, and K. Bay. 2012. 2011 Post-Construction Monitoring Study, Criterion Wind Project, Garrett County, Maryland: April - November 2011. Revised November 25, 2013.

8. Arnett EB, Schirmacher MR, Hein CD, Huso MMP (2011) Patterns of Bird and Bat Fatality at the Locust Ridge II Wind Project, Pennsylvania. 2009-2010 Final Report.

9. Young, D.P. Jr., Bay K, Nomani S, Tidhar W (2009) Nedpower Mount Storm Wind Energy Facility, Post-Construction Avian and Bat Monitoring: March - June 2009.

10. Young, D.P. Jr., Bay K, Nomani S, Tidhar W (2010) Nedpower Mount Storm Wind Energy Facility, Post-Construction Avian and Bat Monitoring: July - October 2009.

11. Young, D.P. Jr., Bay K, Nomani S, Tidhar W (2010) Nedpower Mount Storm Wind Energy Facility, Post-Construction Avian and Bat Monitoring: April - July 2010.

12. Young, D.P. Jr., Nomani S, Tidhar W, Bay K (2011) Nedpower Mount Storm Wind Energy Facility, Post-Construction Avian and Bat Monitoring: July - October 2010.

13. Young, D.P. Jr., Nomani S, Courage Z, Bay K (2011) Nedpower Mount Storm Wind Energy Facility, Post-Construction Avian and Bat Monitoring: April - July 2011.

14. Young, D.P. Jr., Nomani S, Courage Z, Bay K (2012) Nedpower Mount Storm Wind Energy Facility, Post-Construction Avian and Bat Monitoring: July - October 2011.

15. Kerns J, Kerlinger P (2004) A Study of Bird and Bat Collisions at the Mountaineer Wind Energy Facility, Tucker County, West Virginia: Annual Report for 2003. Technical report prepared by Curry and Kerlinger, LLC., for FPL Energy and Mountaineer Wind Energy Center Technical Review Committee. Curry and Kerlinger, LLC. 39 pp p.

16. Stantec Consulting, Inc. (Stantec) (2009) Post-Construction Monitoring at the Munnsville Wind Farm, New York: 2008.

17. Jain A, Kerlinger P, Curry R, Slobodnik L, Quant J, et al. (2009) Annual Report for the Noble Bliss Windpark, LLC, Postconstruction Bird and Bat Fatality Study - 2008.

18. Jain A, Kerlinger P, Slobodnik L, Curry R, Fuerst A, et al. (2010) Annual Report for the Noble Bliss Windpark, LLC: Postconstruction Bird and Bat Fatality Study - 2009.

19. Jain A, Kerlinger P, Slobodnik L, Curry R, Harte A (2011) Annual Report for the Noble Wethersfield Windpark, LLC: Postconstruction Bird and Bat Fatality Study - 2010.

20. Jacques Whitford Stantec Limited (Jacques Whitford) (2009) Ripley Wind Power Project Postconstruction Monitoring Report.

21. Tidhar D, McManus L, Courage Z, Tidhar WL (2012) 2010 Post-Construction Fatality Monitoring Study and Bat Acoustic Study for the High Sheldon Wind Farm, Wyoming County, New York. Final Report: April 15 - November 15, 2010.

22. Tidhar D, McManus L, Solick D, Courage Z, Bay K (2012) 2011 Post-Construction Fatality Monitoring Study and Bat Acoustic Study for the High Sheldon Wind Farm, Wyoming County, New York. Final Report: April 15 - November 15, 2011.

23. Kronner K, Gritski R, Downes S (2008) Big Horn Wind Power Project Wildlife Fatality Monitoring Study: 2006−2007.

24. Jeffrey JD, Bay K, Erickson WP, Sonneberg M, Baker J, et al. (2009) Portland General Electric Biglow Canyon Wind Farm Phase I Post-Construction Avian and Bat Monitoring First Annual Report, Sherman County, Oregon. January 2008 - December 2008.

25. Enk T, Bay K, Sonnenberg M, Baker J, Kesterke M, et al. (2010) Biglow Canyon Wind Farm Phase I Post-Construction Avian and Bat Monitoring Second Annual Report, Sherman County, Oregon. January 26, 2009 - December 11, 2009.

26. Enk T, Bay K, Sonnenberg M, Flaig J, Boehrs JR, et al. (2011) Year 1 Post-Construction Avian and Bat Monitoring Report: Biglow Canyon Wind Farm Phase II, Sherman County, Oregon. September 10, 2009 - September 12, 2010.

27. Enk T, Bay K, Sonnenberg M, Boehrs JR (2012) Year 2 Avian and Bat Monitoring Report: Biglow Canyon Wind Farm Phase II, Sherman County, Oregon. September 13, 2010 - September 12, 2011.

28. Enk T, Bay K, Sonnenberg M, Boehrs JR (2012) Year 1 Avian and Bat Monitoring Report: Biglow Canyon Wind Farm Phase III, Sherman County, Oregon. September 13, 2010 - September 9, 2011.

29. Young, D.P. Jr., Jeffrey J, Erickson WP, Bay K, Poulton VK, et al. (2006) Eurus Combine Hills Turbine Ranch. Phase 1 Post Construction Wildlife Monitoring First Annual Report: February 2004 - February 2005.

30. Enz T, Bay K, Sonnenberg M, Palochak A (2012) Post-Construction Monitoring Studies for the Combine Hills Turbine Ranch, Umatilla County, Oregon. Final Report: January 7 - December 2, 2011.

31. Thompson J, Solick D, Bay K (2011) Post-Construction Fatality Surveys for the Dry Lake Phase I Wind Project. Iberdrola Renewables: September 2009 - November 2010.

32. Thompson J, Bay K (2012) Post-Construction Fatality Surveys for the Dry Lake II Wind Project: February 2011 – February 2012.

33. Jeffrey JD, Erickson WP, Bay K, Sonneberg M, Baker J, et al. (2009) Horizon Wind Energy, Elkhorn Valley Wind Project, Post-Construction Avian and Bat Monitoring, First Annual Report, January-December 2008.

34. Enk T, Derby C, Bay K, Sonnenberg M (2011) 2010 Post-Construction Fatality Monitoring Report, Elkhorn Valley Wind Farm, Union County, Oregon. January – December 2010.

35. Young, D.P. Jr., Erickson WP, Good RE, Strickland MD, Johnson GD (2003) Avian and Bat Mortality Associated with the Initial Phase of the Foote Creek Rim Windpower Project, Carbon County, Wyoming, Final Report, November 1998 - June 2002.

36. URS Corporation (2010) Final Goodnoe Hills Wind Project Avian Mortality Monitoring Report.

37. Downes S, Gritski R (2012) Harvest Wind Project Wildlife Monitoring Report: January 2010 – January 2012.

38. Gritski R, Kronner K (2010) Hay Canyon Wind Power Project Wildlife Monitoring Study: May 2009 - May 2010.

39. Young, D.P. Jr., Erickson WP, Jeffrey J, Poulton VK (2007) Puget Sound Energy Hopkins Ridge Wind Project Phase 1 Post-Construction Avian and Bat Monitoring First Annual Report, January - December 2006. 25 pp. p.

40. Young, D.P. Jr., Jeffrey JD, Bay K, Erickson WP (2009) Puget Sound Energy Hopkins Ridge Wind Project, Phase 1, Columbia County, Washington. Post-Construction Avian and Bat Monitoring, Second Annual Report: January - December, 2008.

41. Stantec Consulting Services, Inc. (Stantec Consulting Services) (2012) Post-Construction Monitoring, Summer 2011 - Spring 2012, Year 1 Annual Report: Kittitas Valley Wind Power Project, Cle Elum, Washington.

42. Johnson GD, Erickson WP, White J (2003) Avian and Bat Mortality During the First Year of Operation at the Klondike Phase I Wind Project, Sherman County, Oregon.

43. Northwest Wildlife Consultants, Inc. (NWC), Western EcoSystems Technology, Inc. (WEST) (2007) Avian and Bat Monitoring Report for the Klondike II Wind Power Project. Sherman County, Oregon.

44. Gritski R, Downes S, Kronner K (2010) Klondike III (Phase 1) Wind Power Project Wildlife Monitoring: October 2007-October 2009.

45. Gritski R, Downes S, Kronner K (2011) Klondike IIIa (Phase 2) Wind Power Project Wildlife Monitoring: August 2008 - August 2010.

46. Gritski R, Kronner K, Downes S (2008) Leaning Juniper Wind Power Project, 2006 − 2008. Wildlife Monitoring Final Report.

47. Enz T, Bay K (2011) Post-Construction Monitoring at the Linden Ranch Wind Farm, Klickitat County, Washington. Final Report: June 30, 2010 - July 17, 2011.

48. URS Corporation (2010) Final Marengo I Wind Project Year One Avian Mortality Monitoring Report.

49. URS Corporation (2010) Final Marengo II Wind Project Year One Avian Mortality Monitoring Report.

50. Erickson WP, Kronner K, Gritski R (2003) Nine Canyon Wind Power Project Avian and Bat Monitoring Report. September 2002 – August 2003.

51. Gritski R, Kronner K (2010) Pebble Springs Wind Power Project Wildlife Monitoring Study: January 2009 - January 2010.

52. Erickson WP, Jeffrey J, Kronner K, Bay K (2004) Stateline Wind Project Wildlife Monitoring Annual Report. July 2001 - December 2003.

53. Erickson WP, Kronner K, Bay KJ (2007) Stateline 2 Wind Project Wildlife Monitoring Report, January - December 2006.

54. Brown WK, Hamilton BL (2006) Monitoring of Bird and Bat Collisions with Wind Turbines at the Summerview Wind Power Project, Alberta: 2005-2006.

55. Enz T, Bay K (2010) Post-Construction Avian and Bat Fatality Monitoring Study, Tuolumne Wind Project, Klickitat County, Washington. Final Report: April 20, 2009 - April 7, 2010.

56. Erickson WP, Johnson GD, Strickland MD, Kronner K (2000) Avian and Bat Mortality Associated with the Vansycle Wind Project, Umatilla County, Oregon: 1999 Study Year.

57. Ventus Environmental Solutions (Ventus) (2012) Vantage Wind Energy Center Avian and Bat Monitoring Study: March 2011- March 2012.

58. Downes S, Gritski R (2012) White Creek Wind I Wildlife Monitoring Report: November 2007 - November 2011.

59. Erickson WP, Jeffrey J, Poulton VK (2008) Avian and Bat Monitoring: Year 1 Report. Puget Sound Energy Wild Horse Wind Project, Kittitas County, Washington.

60. Enz T, Bay K, Nomani S, Kesterke M (2011) Bird and Bat Fatality Monitoring Study, Windy Flats and Windy Point II Wind Energy Projects, Klickitat County, Washington. Final Report: February 1, 2010 - January 14, 2011.

61. Tidhar D, Tidhar W, Sonnenberg M (2010) Post-Construction Fatality Surveys for Lempster Wind Project, Iberdrola Renewables. Prepared for Lempster Wind, LLC, Lempster Wind Technical Advisory Committee, and Iberdrola Renewables, Inc.

62. Tidhar D, Tidhar WL, McManus L, Courage Z (2011) 2010 Post-Construction Fatality Surveys for the Lempster Wind Project, Lempster, New Hampshire.

63. Jain A, Kerlinger P, Curry R, Slobodnik L (2009) Annual Report for the Maple Ridge Wind Power Project: Post-Construction Bird and Bat Fatality Study - 2007.

64. Jain A, Kerlinger P, Curry R, Slobodnik L, Lehman M (2009) Maple Ridge Wind Power Avian and Bat Fatality Study Report - 2008.

65. Stantec Consulting, Inc. (Stantec) (2008) 2007 Spring, Summer, and Fall Post-Construction Bird and Bat Mortality Study at the Mars Hill Wind Farm, Maine.

66. Stantec Consulting, Inc. (Stantec) (2009) Post-Construction Monitoring at the Mars Hill Wind Farm, Maine - Year 2, 2008.

67. Jain A, Kerlinger P, Slobodnik L, Curry R, Russell K (2011) Annual Report for the Noble Altona Windpark, LLC: Postconstruction Bird and Bat Fatality Study - 2010.

68. Jain A, Kerlinger P, Slobodnik L, Curry R, Russell K (2011) Annual Report for the Noble Chateaugay Windpark, LLC: Postconstruction Bird and Bat Fatality Study - 2010.

69. Jain A, Kerlinger P, Curry R, Slobodnik L, Histed J, et al. (2009) Annual Report for the Noble Clinton Windpark, LLC, Postconstruction Bird and Bat Fatality Study - 2008.

70. Jain A, Kerlinger P, Slobodnik L, Curry R, Russell K (2010) Annual Report for the Noble Clinton Windpark, LLC: Postconstruction Bird and Bat Fatality Study - 2009.

71. Jain A, Kerlinger P, Curry R, Slobodnik L, Fuerst A, et al. (2009) Annual Report for the Noble Ellenburg Windpark, LLC, Postconstruction Bird and Bat Fatality Study - 2008.

72. Jain A, Kerlinger P, Slobodnik L, Curry R, Russell K (2010) Annual Report for the Noble Ellenburg Windpark, LLC: Postconstruction Bird and Bat Fatality Study - 2009.

73. Stantec Consulting, Inc. (Stantec) (2009) Stetson I Mountain Wind Project: Year 1 Post-Construction Monitoring Report, 2009 for the Stetson Mountain Wind Project in Penobscot and Washington Counties, Maine.

74. Normandeau Associates, Inc. (2011) Year 3 Post- Construction Avian and Bat Casualty Monitoring at the Stetson I Wind Farm, T8 R4 NBPP, Maine.

75. Normandeau Associates, Inc. (2010) Stetson Mountain II Wind Project Year 1 Post-Construction Avian and Bat Mortality Monitoring Study, T8 R4 NBPP, Maine.

76. Chatfield A, Erickson WP, Bay K (2010) Final Report: Avian and Bat Fatality Study at the Alite Wind-Energy Facility, Kern County, California. Final Report: June 15, 2009 – June 15, 2010.

77. Western EcoSystems Technology, Inc. (WEST) (2006) Diablo Winds Wildlife Monitoring Progress Report, March 2005 - February 2006. Cheyenne, Wyoming: WEST.

78. Western EcoSystems Technology, Inc. (WEST) (2008) Diablo Winds Wildlife Monitoring Progress Report: March 2005 – February 2007.

79. Chatfield A, Erickson W, Bay K (2009) Avian and Bat Fatality Study, Dillon Wind-Energy Facility, Riverside County, California. Final Report: March 26, 2008 - March 26, 2009.

80. Kerlinger P, Curry R, Culp L, Jain A, Wilkerson C, et al. (2006) Post-Construction Avian and Bat Fatality Monitoring for the High Winds Wind Power Project, Solano County, California: Two Year Report.

81. BioResource Consultants, Inc. (BRC) (2010) 2009/2010 Annual Report: Bird and Bat Mortality Monitoring, Pine Tree Wind Farm, Kern County, California.

82. Kerlinger P, Curry R, Culp L, Hasch A, Jain A (2009) Post-Construction Avian Monitoring Study for the Shiloh I Wind Power Project, Solano County, California. Final Report: October 2009.

83. Kerlinger P, Curry R, Culp L, Hasch A, Jain A (2010) Post-Construction Avian Monitoring Study for the Shiloh II Wind Power Project, Solano County, California. Year One Report.

84. Western EcoSystems Technology, Inc. (WEST) (2011) Post-Construction Fatality Surveys for the Barton Chapel Wind Project: Iberdrola Renewables. Version: July 2011.

85. Derby C, Chodachek K, Bay K, Nomani S (2011) Post-Construction Fatality Surveys for the Barton I and II Wind Project: IRI. March 2010 - February 2011.

86. Gruver J, Sonnenberg M, Bay K, Erickson W (2009) Post-Construction Bat and Bird Fatality Study at the Blue Sky Green Field Wind Energy Center, Fond Du Lac County, Wisconsin July 21 - October 31, 2008 and March 15 - June 4, 2009.

87. Tierney R (2007) Buffalo Gap I Wind Farm Avian Mortality Study: February 2006-January 2007.

88. Tierney R (2009) Buffalo Gap 2 Wind Farm Avian Mortality Study: July 2007 - December 2008. Final Survey Report.

89. Johnson GD, Erickson WP, Strickland MD, Shepherd MF, Shepherd DA (2000) Avian Monitoring Studies at the Buffalo Ridge Wind Resource Area, Minnesota: Results of a 4-Year Study. 212 pp.

90. Derby C, Chodachek K, Bay K, Merrill A (2010) Post-Construction Fatality Survey for the Buffalo Ridge I Wind Project. May 2009 - May 2010.

91. Derby C, Chodachek K, Sonnenberg M (2012) Post-Construction Casualty Surveys for the Buffalo Ridge II Wind Project. Iberdrola Renewables: March 2011- February 2012.

92. BHE Environmental, Inc. (BHE) (2010) Post-Construction Bird and Bat Mortality Study: Cedar Ridge Wind Farm, Fond Du Lac County, Wisconsin.

93. BHE Environmental, Inc. (BHE) (2011) Post-Construction Bird and Bat Mortality Study: Cedar Ridge Wind Farm, Fond Du Lac County, Wisconsin.

94. Derby C, Chodachek K, Bay K, Merrill A (2010) Post-Construction Fatality Surveys for the Elm Creek Wind Project: March 2009- February 2010.

95. Derby C, Chodachek K, Sonnenberg M (2012) Post-Construction Fatality Surveys for the Elm Creek II Wind Project. Iberdrola Renewables: March 2011-February 2012.

96. Derby C, Ritzert J, Bay K (2010) Bird and Bat Fatality Study, Grand Ridge Wind Resource Area, Lasalle County, Illinois. January 2009 - January 2010.

97. Howe RW, Evans W, Wolf AT (2002) Effects of Wind Turbines on Birds and Bats in Northeastern Wisconsin. 104 pp p.

98. Derby C, Chodachek K, Bay K, Merrill A (2010) Post-Construction Fatality Surveys for the Moraine II Wind Project: March - December 2009.

99. Derby C, Dahl A, Erickson W, Bay K, Hoban J (2007) Post-Construction Monitoring Report for Avian and Bat Mortality at the NPPD Ainsworth Wind Farm. Unpublished report prepared by Western EcoSystems Technology, Inc. (WEST), Cheyenne, Wyoming, for the Nebraska Public Power District.

100. Chodachek K, Derby C, Sonnenberg M, Thorn T (2012) Post-Construction Fatality Surveys for the Pioneer Prairie Wind Farm I LLC Phase II, Mitchell County, Iowa: April 4, 2011 – March 31, 2012.

101. Derby C, Dahl A, Merrill A (2012) Post-Construction Monitoring Results for the PrairieWinds SD1 Wind Energy Facility, South Dakota. Final Report: March 2011 - February 2012.

102. Derby C, Chodachek K, Thorn T, Bay K, Nomani S (2011) Post-Construction Fatality Surveys for the PrairieWinds ND1 Wind Facility, Basin Electric Power Cooperative, March - November 2010.

103. Derby C, Chodachek K, Thorn T, Merrill A (2012) Post-Construction Surveys for the PrairieWinds ND1 (2011) Wind Facility Basin Electric Power Cooperative: March - October 2011.

104. Derby C, Chodachek K, Bay K, Nomani S (2011) Post-Construction Fatality Surveys for the Rugby Wind Project: Iberdrola Renewables, Inc. March 2010 - March 2011.

105. Jain A (2005) Bird and Bat Behavior and Mortality at a Northern Iowa Windfarm. Thesis. Ames, Iowa: Iowa State University.

106. Derby C, Dahl A, Merrill A, Bay K (2010) 2009 Post-Construction Monitoring Results for the Wessington Springs Wind-Energy Facility, South Dakota. Final Report.

107. Derby C, Dahl A, Bay K, McManus L (2011) 2010 Post-Construction Monitoring Results for the Wessington Springs Wind Energy Facility, South Dakota. Final Report: March 9 – November 16, 2010.

108. Derby C, Chodachek K, Bay K, Merrill A (2010) Post-Construction Fatality Surveys for the Winnebago Wind Project: March 2009- February 2010.
