# Supplementary material for: A Comprehensive Analysis of Small-Passerine Fatalities from Collision with Turbines at Wind Energy Facilities
Source: PLoS One. 2014 Sep 15;9(9):e107491. doi: 10.1371/journal.pone.0107491 (PMC4164633; doi:10.1371/journal.pone.0107491)
Supplement: Appendix S8 — The number and percent composition of fatalities of small passerines found during 116 studies of bird collisions with wind energy turbines, geographically separated into avifaunal biomes, along with the estimated annual number of fatalities using the lowest and highest bias adjustment value. Blank spaces indicate no fatalities were recorded. See Appendix S10 for scientific names of each species. Column A = number of fatalities found in studies in biome, B = % composition in biome, C = estimated number of fatalities each year adjusted by the bias value that was the lowest, D = estimated number of fatalities each year adjusted by the bias value that was the highest. (DOCX) [file pone.0107491.s035.docx]

**Appendix S8. The number and percent composition of fatalities of small passerines found during 116 studies of bird collisions with wind energy turbines, geographically separated into avifaunal biomes, along with the estimated annual number of fatalities using the lowest and highest bias adjustment value.** Blank spaces indicate no fatalities were recorded. See Appendix S10 for scientific names of each species. Column A = number of fatalities found in studies in biome, B = proportion of total in biome, C = estimated number of fatalities each year adjusted by the bias value that was the lowest, D = estimated number of fatalities each year adjusted by the bias value that was the highest.

|  | **Avifaunal Biome** | | | | | | | | | | | | | | | | | | | |
| --- | --- | --- | --- | --- | --- | --- | --- | --- | --- | --- | --- | --- | --- | --- | --- | --- | --- | --- | --- | --- |
| **Species** | **Eastern** | | | | **Intermountain West** | | | | **Northern Forest** | | | | **Pacific*** | | | | **Prairie** | | | |
|  | A | B | C | D | A | B | C | D | A | B | C | D | A | B | C | D | A | B | C | D |
| Acadian flycatcher | 2 | <0.01 | 66 | 44 |  | - | - | - |  | - | - | - |  | - | - | - |  | - | - | - |
| alder flycatcher |  | - | - | - |  | - | - | - | 1 | <0.01 | 19 | 15 |  | - | - | - |  | - | - | - |
| American goldfinch | 1 | <0.01 | 33 | 22 | 3 | <0.01 | 71 | 45 | 3 | 0.01 | 57 | 46 | 1 | <0.01 | 13 | 10 | 3 | 0.01 | 1,610 | 873 |
| American pipit |  | - | - | - | 2 | <0.01 | 48 | 30 |  | - | - | - | 11 | 0.02 | 146 | 114 |  | - | - | - |
| American redstart | 12 | 0.02 | 394 | 265 |  | - | - | - | 4 | 0.01 | 76 | 61 |  | - | - | - | 2 | 0.01 | 1,073 | 582 |
| American robin | 7 | 0.01 | 230 | 155 | 14 | 0.01 | 333 | 208 | 6 | 0.02 | 115 | 92 |  | - | - | - | 1 | <0.01 | 537 | 291 |
| American tree sparrow |  | - | - | - |  | - | - | - |  | - | - | - |  | - | - | - | 7 | 0.03 | 3,756 | 2,038 |
| ash-throated flycatcher |  | - | - | - | 1 | <0.01 | 24 | 15 |  | - | - | - | 1 | <0.01 | 13 | 10 |  | - | - | - |
| bank swallow | 1 | <0.01 | 33 | 22 |  | - | - | - |  | - | - | - |  | - | - | - |  | - | - | - |
| barn swallow | 1 | <0.01 | 33 | 22 |  | - | - | - | 1 | <0.01 | 19 | 15 |  | - | - | - | 9 | 0.03 | 4,829 | 2,620 |
| bay-breasted warbler | 13 | 0.02 | 427 | 288 |  | - | - | - | 5 | 0.02 | 96 | 77 |  | - | - | - |  | - | - | - |
| Bell's vireo |  | - | - | - |  | - | - | - |  | - | - | - |  | - | - | - | 1 | <0.01 | 537 | 291 |
| Bewick's wren |  | - | - | - |  | - | - | - |  | - | - | - | 1 | <0.01 | 13 | 10 |  | - | - | - |
| black-and-white warbler | 7 | 0.01 | 230 | 155 |  | - | - | - | 4 | 0.01 | 76 | 61 |  | - | - | - | 3 | 0.01 | 1,610 | 873 |
| Blackburnian warbler | 7 | 0.01 | 230 | 155 |  | - | - | - | 3 | 0.01 | 57 | 46 |  | - | - | - |  | - | - | - |
| black-capped chickadee |  | - | - | - |  | - | - | - | 1 | <0.01 | 19 | 15 |  | - | - | - |  | - | - | - |
| black-headed grosbeak |  | - | - | - |  | - | - | - |  | - | - | - | 7 | 0.02 | 93 | 73 |  | - | - | - |
| blackpoll warbler | 43 | 0.06 | 1,411 | 951 |  | - | - | - | 6 | 0.02 | 115 | 92 |  | - | - | - | 1 | <0.01 | 537 | 291 |
| black-tailed gnatcatcher |  | - | - | - |  | - | - | - |  | - | - | - | 1 | <0.01 | 13 | 10 |  | - | - | - |
| black-throated blue warbler | 23 | 0.03 | 755 | 509 | 1 | <0.01 | 24 | 15 | 3 | 0.01 | 57 | 46 |  | - | - | - |  | - | - | - |
| black-throated gray warbler |  | - | - | - | 5 | <0.01 | 119 | 74 |  | - | - | - | 4 | 0.01 | 53 | 42 |  | - | - | - |
| black-throated green warbler | 12 | 0.02 | 394 | 265 |  | - | - | - | 4 | 0.01 | 76 | 61 |  | - | - | - | 1 | <0.01 | 537 | 291 |
| black-throated sparrow |  | - | - | - | 2 | <0.01 | 48 | 30 |  | - | - | - | 2 | <0.01 | 27 | 21 |  | - | - | - |
| blue jay | 3 | <0.01 | 98 | 66 |  | - | - | - | 3 | 0.01 | 57 | 46 |  | - | - | - | 1 | <0.01 | 537 | 291 |
| blue-headed vireo | 8 | 0.01 | 263 | 177 |  | - | - | - | 6 | 0.02 | 115 | 92 |  | - | - | - |  | - | - | - |
| blue-winged warbler | 2 | <0.01 | 66 | 44 |  | - | - | - |  | - | - | - |  | - | - | - |  | - | - | - |
| bobolink | 17 | 0.02 | 558 | 376 |  | - | - | - | 5 | 0.02 | 96 | 77 |  | - | - | - |  | - | - | - |
| Brewer's blackbird |  | - | - | - | 2 | <0.01 | 48 | 30 |  | - | - | - | 20 | 0.04 | 266 | 208 | 2 | 0.01 | 1,073 | 582 |
| Brewer's sparrow |  | - | - | - | 17 | 0.01 | 404 | 252 |  | - | - | - |  | - | - | - |  | - | - | - |
| brown creeper | 2 | <0.01 | 66 | 44 | 3 | <0.01 | 71 | 45 | 3 | 0.01 | 57 | 46 |  | - | - | - | 2 | 0.01 | 1,073 | 582 |
| brown thrasher |  | - | - | - |  | - | - | - |  | - | - | - |  | - | - | - | 3 | 0.01 | 1,610 | 873 |
| brown-headed cowbird |  | - | - | - | 1 | <0.01 | 24 | 15 |  | - | - | - |  | - | - | - | 4 | 0.01 | 2,146 | 1,165 |
| Bullock's oriole |  | - | - | - |  | - | - | - |  | - | - | - | 2 | <0.01 | 27 | 21 |  | - | - | - |
| bushtit |  | - | - | - |  | - | - | - |  | - | - | - |  | - | - | - | 1 | <0.01 | 537 | 291 |
| Canada warbler | 6 | 0.01 | 197 | 133 |  | - | - | - |  | - | - | - |  | - | - | - |  | - | - | - |
| Cape May warbler | 12 | 0.02 | 394 | 265 |  | - | - | - |  | - | - | - |  | - | - | - | 1 | <0.01 | 537 | 291 |
| Cassin's vireo |  | - | - | - | 4 | <0.01 | 95 | 59 |  | - | - | - | 1 | <0.01 | 13 | 10 |  | - | - | - |
| cedar waxwing | 3 | <0.01 | 98 | 66 | 1 | <0.01 | 24 | 15 | 8 | 0.03 | 153 | 123 |  | - | - | - | 3 | 0.01 | 1,610 | 873 |
| cerulean warbler | 1 | <0.01 | 33 | 22 |  | - | - | - |  | - | - | - |  | - | - | - |  | - | - | - |
| chestnut-collared longspur |  | - | - | - | 2 | <0.01 | 48 | 30 |  | - | - | - |  | - | - | - |  | - | - | - |
| chestnut-sided warbler | 15 | 0.02 | 492 | 332 |  | - | - | - | 1 | <0.01 | 19 | 15 |  | - | - | - | 2 | 0.01 | 1,073 | 582 |
| chipping sparrow |  | - | - | - | 10 | 0.01 | 238 | 148 |  | - | - | - | 1 | <0.01 | 13 | 10 |  | - | - | - |
| cliff swallow | 2 | <0.01 | 66 | 44 | 7 | 0.01 | 166 | 104 | 1 | <0.01 | 19 | 15 |  | - | - | - | 3 | 0.01 | 1,610 | 873 |
| common grackle |  | - | - | - |  | - | - | - | 1 | <0.01 | 19 | 15 |  | - | - | - | 4 | 0.01 | 2,146 | 1,165 |
| common yellowthroat | 11 | 0.01 | 361 | 243 | 2 | <0.01 | 48 | 30 | 3 | 0.01 | 57 | 46 | 2 | <0.01 | 27 | 21 | 8 | 0.03 | 4,292 | 2,329 |
| dark-eyed junco | 1 | <0.01 | 33 | 22 | 38 | 0.03 | 904 | 564 | 1 | <0.01 | 19 | 16 | 1 | <0.01 | 13 | 10 | 11 | 0.04 | 5,902 | 3,202 |
| dickcissel |  | - | - | - |  | - | - | - |  | - | - | - |  | - | - | - | 3 | 0.01 | 1,610 | 873 |
| eastern bluebird | 3 | <0.01 | 98 | 66 |  | - | - | - |  | - | - | - |  | - | - | - |  | - | - | - |
| eastern kingbird | 3 | <0.01 | 98 | 66 |  | - | - | - | 8 | 0.03 | 153 | 123 |  | - | - | - | 4 | 0.01 | 2,146 | 1,165 |
| eastern meadowlark |  | - | - | - |  | - | - | - |  | - | - | - |  | - | - | - | 3 | 0.01 | 1,610 | 873 |
| eastern phoebe | 1 | <0.01 | 33 | 22 |  | - | - | - |  | - | - | - |  | - | - | - |  | - | - | - |
| eastern towhee | 4 | 0.01 | 131 | 88 |  | - | - | - | 1 | <0.01 | 19 | 15 |  | - | - | - |  | - | - | - |
| eastern wood-pewee | 1 | <0.01 | 33 | 22 |  | - | - | - | 3 | 0.01 | 57 | 46 |  | - | - | - |  | - | - | - |
| European starling | 7 | 0.01 | 230 | 155 | 53 | 0.04 | 1,261 | 787 | 14 | 0.05 | 268 | 215 | 19 | 0.04 | 253 | 197 | 10 | 0.04 | 5,365 | 2,911 |
| evening grosbeak |  | - | - | - |  | - | - | - | 1 | <0.01 | 19 | 15 |  | - | - | - |  | - | - | - |
| field sparrow | 5 | 0.01 | 164 | 111 |  | - | - | - |  | - | - | - |  | - | - | - | 2 | 0.01 | 1,073 | 582 |
| fox sparrow |  | - | - | - |  | - | - | - |  | - | - | - |  | - | - | - | 1 | <0.01 | 537 | 291 |
| golden-crowned kinglet | 38 | 0.05 | 1,247 | 840 | 87 | 0.06 | 2,069 | 1,292 | 20 | 0.07 | 382 | 307 | 1 | <0.01 | 13 | 10 | 12 | 0.04 | 6,439 | 3,494 |
| golden-crowned sparrow |  | - | - | - | 5 | <0.01 | 119 | 74 |  | - | - | - | 1 | <0.01 | 13 | 10 |  | - | - | - |
| grasshopper sparrow |  | - | - | - | 1 | <0.01 | 24 | 15 |  | - | - | - |  | - | - | - | 4 | 0.01 | 2,146 | 1,165 |
| gray catbird | 6 | 0.01 | 197 | 133 | 1 | <0.01 | 24 | 15 | 1 | <0.01 | 19 | 15 |  | - | - | - | 3 | 0.01 | 1,610 | 873 |
| gray flycatcher |  | - | - | - | 2 | <0.01 | 48 | 30 |  | - | - | - |  | - | - | - |  | - | - | - |
| gray vireo |  | - | - | - | 1 | <0.01 | 24 | 15 |  | - | - | - |  | - | - | - |  | - | - | - |
| gray-cheeked thrush | 7 | 0.01 | 230 | 155 |  | - | - | - |  | - | - | - |  | - | - | - | 1 | <0.01 | 537 | 291 |
| green-tailed towhee |  | - | - | - | 4 | <0.01 | 95 | 59 |  | - | - | - |  | - | - | - |  | - | - | - |
| Hammond's flycatcher |  | - | - | - | 3 | <0.01 | 71 | 45 |  | - | - | - | 2 | <0.01 | 27 | 21 |  | - | - | - |
| hermit thrush | 4 | 0.01 | 131 | 88 | 3 | <0.01 | 71 | 45 | 2 | 0.01 | 38 | 31 | 1 | <0.01 | 13 | 10 |  | - | - | - |
| hooded warbler | 4 | 0.01 | 131 | 88 |  | - | - | - |  | - | - | - |  | - | - | - |  | - | - | - |
| horned lark | 4 | 0.01 | 131 | 88 | 608 | 0.45 | 14,461 | 9,027 |  | - | - | - | 44 | 0.10 | 586 | 457 | 25 | 0.09 | 13,414 | 7,278 |
| house finch |  | - | - | - | 5 | <0.01 | 119 | 74 |  | - | - | - | 3 | 0.01 | 40 | 31 |  | - | - | - |
| house sparrow | 3 | <0.01 | 98 | 66 | 10 | 0.01 | 238 | 148 |  | - | - | - | 1 | <0.01 | 13 | 10 | 1 | <0.01 | 537 | 291 |
| house wren |  | - | - | - | 13 | 0.01 | 309 | 193 |  | - | - | - | 1 | <0.01 | 13 | 10 | 6 | 0.02 | 3,219 | 1,747 |
| indigo bunting | 1 | <0.01 | 33 | 22 |  | - | - | - | 2 | 0.01 | 38 | 31 |  | - | - | - |  | - | - | - |
| Kentucky warbler | 2 | <0.01 | 66 | 44 |  | - | - | - |  | - | - | - |  | - | - | - |  | - | - | - |
| Lapland longspur |  | - | - | - |  | - | - | - |  | - | - | - |  | - | - | - | 2 | 0.01 | 1,073 | 582 |
| lark bunting |  | - | - | - | 1 | <0.01 | 24 | 15 |  | - | - | - |  | - | - | - |  | - | - | - |
| lark sparrow |  | - | - | - |  | - | - | - |  | - | - | - | 2 | <0.01 | 27 | 21 |  | - | - | - |
| Le Conte's sparrow |  | - | - | - |  | - | - | - |  | - | - | - |  | - | - | - | 2 | 0.01 | 1,073 | 582 |
| least flycatcher |  | - | - | - |  | - | - | - | 1 | <0.01 | 19 | 15 |  | - | - | - | 1 | <0.01 | 537 | 291 |
| lesser goldfinch |  | - | - | - |  | - | - | - |  | - | - | - | 1 | <0.01 | 13 | 10 |  | - | - | - |
| Lincoln's sparrow | 3 | <0.01 | 98 | 66 | 7 | 0.01 | 166 | 104 |  | - | - | - | 4 | 0.01 | 53 | 42 | 1 | <0.01 | 537 | 291 |
| loggerhead shrike |  | - | - | - |  | - | - | - |  | - | - | - | 4 | 0.01 | 53 | 42 | 1 | <0.01 | 537 | 291 |
| MacGillivray's warbler |  | - | - | - | 5 | <0.01 | 119 | 74 |  | - | - | - | 3 | 0.01 | 40 | 31 |  | - | - | - |
| magnolia warbler | 38 | 0.05 | 1,247 | 840 |  | - | - | - | 18 | 0.06 | 344 | 277 |  | - | - | - | 4 | 0.01 | 2,146 | 1,165 |
| marsh wren |  | - | - | - |  | - | - | - |  | - | - | - |  | - | - | - | 2 | 0.01 | 1,073 | 582 |
| mountain bluebird |  | - | - | - | 6 | <0.01 | 143 | 89 |  | - | - | - |  | - | - | - |  | - | - | - |
| mourning warbler | 1 | <0.01 | 33 | 22 |  | - | - | - |  | - | - | - |  | - | - | - |  | - | - | - |
| Nashville warbler |  | - | - | - |  | - | - | - | 1 | <0.01 | 19 | 15 | 1 | <0.01 | 13 | 10 |  | - | - | - |
| northern mockingbird |  | - | - | - | 2 | <0.01 | 48 | 30 | 1 | <0.01 | 19 | 15 | 2 | <0.01 | 27 | 21 | 1 | <0.01 | 537 | 291 |
| northern Parula | 3 | <0.01 | 98 | 66 |  | - | - | - | 3 | 0.01 | 57 | 46 |  | - | - | - |  | - | - | - |
| northern rough-winged swallow |  | - | - | - | 2 | <0.01 | 48 | 30 |  | - | - | - |  | - | - | - | 1 | <0.01 | 537 | 291 |
| northern shrike |  | - | - | - | 1 | <0.01 | 24 | 15 |  | - | - | - |  | - | - | - |  | - | - | - |
| northern waterthrush | 2 | <0.01 | 66 | 44 |  | - | - | - | 1 | <0.01 | 19 | 15 |  | - | - | - |  | - | - | - |
| oak titmouse |  | - | - | - |  | - | - | - |  | - | - | - | 1 | <0.01 | 13 | 10 |  | - | - | - |
| orange-crowned warbler |  | - | - | - | 4 | <0.01 | 95 | 59 |  | - | - | - | 5 | 0.01 | 67 | 52 | 6 | 0.02 | 3,219 | 1,747 |
| orchard oriole |  | - | - | - |  | - | - | - |  | - | - | - |  | - | - | - | 1 | <0.01 | 537 | 291 |
| ovenbird | 17 | 0.02 | 558 | 376 |  | - | - | - | 5 | 0.02 | 96 | 77 |  | - | - | - |  | - | - | - |
| Pacific-slope flycatcher |  | - | - | - | 1 | <0.01 | 24 | 15 |  | - | - | - | 3 | 0.01 | 40 | 31 |  | - | - | - |
| palm warbler | 4 | 0.01 | 131 | 88 |  | - | - | - |  | - | - | - |  | - | - | - |  | - | - | - |
| Philadelphia vireo | 3 | <0.01 | 98 | 66 |  | - | - | - | 1 | <0.01 | 19 | 15 |  | - | - | - |  | - | - | - |
| pine siskin | 1 | <0.01 | 33 | 22 | 2 | <0.01 | 48 | 30 |  | - | - | - |  | - | - | - |  | - | - | - |
| pine warbler |  | - | - | - |  | - | - | - | 2 | 0.01 | 38 | 31 |  | - | - | - |  | - | - | - |
| prairie warbler |  | - | - | - |  | - | - | - | 1 | <0.01 | 19 | 15 |  | - | - | - |  | - | - | - |
| purple finch |  | - | - | - | 1 | <0.01 | 24 | 15 | 3 | 0.01 | 57 | 46 |  | - | - | - |  | - | - | - |
| purple martin | 1 | <0.01 | 33 | 22 |  | - | - | - |  | - | - | - |  | - | - | - | 2 | 0.01 | 1,073 | 582 |
| red crossbill |  | - | - | - |  | - | - | - | 1 | <0.01 | 19 | 15 |  | - | - | - |  | - | - | - |
| red-breasted nuthatch | 2 | <0.01 | 66 | 44 | 14 | 0.01 | 333 | 208 | 4 | 0.01 | 76 | 61 |  | - | - | - |  | - | - | - |
| red-eyed vireo | 210 | 0.28 | 6,893 | 4,645 |  | - | - | - | 52 | 0.19 | 994 | 799 |  | - | - | - | 3 | 0.01 | 1,610 | 873 |
| red-winged blackbird |  | - | - | - | 2 | <0.01 | 48 | 30 |  | - | - | - | 61 | 0.13 | 812 | 633 | 7 | 0.03 | 3,756 | 2,038 |
| rock wren |  | - | - | - | 10 | 0.01 | 238 | 148 |  | - | - | - | 4 | 0.01 | 53 | 42 |  | - | - | - |
| rose-breasted grosbeak | 6 | 0.01 | 197 | 133 |  | - | - | - | 2 | 0.01 | 38 | 31 |  | - | - | - | 1 | <0.01 | 537 | 291 |
| ruby-crowned kinglet | 8 | 0.01 | 263 | 177 | 29 | 0.02 | 690 | 431 | 7 | 0.03 | 134 | 108 | 3 | 0.01 | 40 | 31 | 8 | 0.03 | 4,292 | 2,329 |
| sage sparrow |  | - | - | - | 1 | <0.01 | 24 | 15 |  | - | - | - |  | - | - | - |  | - | - | - |
| sage thrasher |  | - | - | - | 3 | <0.01 | 71 | 45 |  | - | - | - |  | - | - | - |  | - | - | - |
| savannah sparrow | 6 | 0.01 | 197 | 133 | 13 | 0.01 | 309 | 193 | 1 | <0.01 | 19 | 15 | 10 | 0.02 | 133 | 104 | 7 | 0.03 | 3,756 | 2,038 |
| Say's phoebe |  | - | - | - | 1 | <0.01 | 24 | 15 |  | - | - | - | 1 | <0.01 | 13 | 10 |  | - | - | - |
| scarlet tanager | 3 | <0.01 | 98 | 66 |  | - | - | - | 1 | <0.01 | 19 | 15 |  | - | - | - |  | - | - | - |
| scissor-tailed flycatcher |  | - | - | - |  | - | - | - |  | - | - | - |  | - | - | - | 1 | <0.01 | 537 | 291 |
| sedge wren |  | - | - | - |  | - | - | - |  | - | - | - |  | - | - | - | 3 | 0.01 | 1,610 | 873 |
| snow bunting |  | - | - | - |  | - | - | - |  | - | - | - |  | - | - | - | 1 | <0.01 | 537 | 291 |
| song sparrow | 1 | <0.01 | 33 | 22 | 4 | <0.01 | 95 | 59 | 2 | 0.01 | 38 | 31 |  | - | - | - | 3 | 0.01 | 1,610 | 873 |
| spotted towhee |  | - | - | - | 5 | <0.01 | 119 | 74 |  | - | - | - | 1 | <0.01 | 13 | 10 | 1 | <0.01 | 537 | 291 |
| Steller's jay |  | - | - | - | 1 | <0.01 | 24 | 15 |  | - | - | - |  | - | - | - |  | - | - | - |
| Swainson's thrush | 14 | 0.02 | 460 | 310 | 1 | <0.01 | 24 | 15 | 2 | 0.01 | 38 | 31 | 1 | <0.01 | 13 | 10 |  | - | - | - |
| swamp sparrow | 1 | <0.01 | 33 | 22 |  | - | - | - |  | - | - | - |  | - | - | - | 2 | 0.01 | 1,073 | 582 |
| Tennessee warbler | 8 | 0.01 | 263 | 177 |  | - | - | - | 1 | <0.01 | 19 | 15 |  | - | - | - | 1 | <0.01 | 537 | 291 |
| Townsend's solitaire |  | - | - | - | 2 | <0.01 | 48 | 30 |  | - | - | - |  | - | - | - |  | - | - | - |
| Townsend's warbler |  | - | - | - | 32 | 0.02 | 761 | 475 |  | - | - | - | 6 | 0.01 | 80 | 62 |  | - | - | - |
| tree swallow | 5 | 0.01 | 164 | 111 | 3 | <0.01 | 71 | 45 | 8 | 0.03 | 153 | 123 | 6 | 0.01 | 80 | 62 | 12 | 0.04 | 6,439 | 3,494 |
| tricolored blackbird |  | - | - | - |  | - | - | - |  | - | - | - | 2 | <0.01 | 27 | 21 |  | - | - | - |
| tufted titmouse | 1 | <0.01 | 33 | 22 |  | - | - | - |  | - | - | - |  | - | - | - |  | - | - | - |
| unidentified blackbird | 2 | <0.01 | 66 | 44 | 2 | <0.01 | 48 | 30 |  | - | - | - | 2 | <0.01 | 27 | 21 |  | - | - | - |
| unidentified bluebird |  | - | - | - |  | - | - | - |  | - | - | - | 2 | <0.01 | 27 | 21 |  | - | - | - |
| unidentified corvid | 4 | 0.01 | 131 | 88 | 1 | <0.01 | 24 | 15 |  | - | - | - |  | - | - | - |  | - | - | - |
| unidentified crowned sparrow |  | - | - | - |  | - | - | - |  | - | - | - | 4 | 0.01 | 53 | 42 |  | - | - | - |
| unidentified empidonax | 4 | 0.01 | 131 | 88 | 2 | <0.01 | 48 | 30 |  | - | - | - | 2 | <0.01 | 27 | 21 | 2 | 0.01 | 1,073 | 582 |
| unidentified flycatcher | 6 | 0.01 | 197 | 133 |  | - | - | - | 4 | 0.01 | 76 | 61 |  | - | - | - | 1 | <0.01 | 537 | 291 |
| unidentified kingbird |  | - | - | - | 2 | <0.01 | 48 | 30 | 3 | 0.01 | 57 | 46 |  | - | - | - |  | - | - | - |
| unidentified kinglet |  | - | - | - | 7 | 0.01 | 166 | 104 | 1 | <0.01 | 19 | 15 |  | - | - | - |  | - | - | - |
| unidentified meadowlark |  | - | - | - |  | - | - | - |  | - | - | - |  | - | - | - | 1 | <0.01 | 537 | 291 |
| unidentified nuthatch |  | - | - | - |  | - | - | - | 1 | <0.01 | 19 | 15 |  | - | - | - |  | - | - | - |
| unidentified passerine | 29 | 0.04 | 952 | 641 | 55 | 0.04 | 1,308 | 817 | 3 | 0.01 | 57 | 46 | 23 | 0.05 | 306 | 239 | 10 | 0.04 | 5,365 | 2,911 |
| unidentified sparrow | 1 | <0.01 | 33 | 22 | 15 | 0.01 | 357 | 223 | 1 | <0.01 | 19 | 15 | 3 | 0.01 | 40 | 31 | 5 | 0.02 | 2,683 | 1,456 |
| unidentified swallow |  | - | - | - | 2 | <0.01 | 48 | 30 |  | - | - | - | 1 | <0.01 | 13 | 10 | 1 | <0.01 | 537 | 291 |
| unidentified thrasher |  | - | - | - |  | - | - | - |  | - | - | - | 2 | <0.01 | 27 | 21 |  | - | - | - |
| unidentified thrush | 2 | <0.01 | 66 | 44 | 2 | <0.01 | 48 | 30 | 1 | <0.01 | 19 | 15 | 1 | <0.01 | 13 | 10 |  | - | - | - |
| unidentified vireo | 3 | <0.01 | 98 | 66 | 4 | <0.01 | 95 | 59 |  | - | - | - |  | - | - | - |  | - | - | - |
| unidentified warbler | 9 | 0.01 | 295 | 199 | 12 | 0.01 | 285 | 178 | 7 | 0.03 | 134 | 108 | 3 | 0.01 | 40 | 31 | 3 | 0.01 | 1,610 | 873 |
| unidentified wren |  | - | - | - | 1 | <0.01 | 24 | 15 | 1 | <0.01 | 19 | 15 |  | - | - | - |  | - | - | - |
| varied thrush |  | - | - | - | 3 | <0.01 | 71 | 45 |  | - | - | - | 1 | <0.01 | 13 | 10 |  | - | - | - |
| veery | 8 | 0.01 | 263 | 177 |  | - | - | - | 1 | <0.01 | 19 | 15 |  | - | - | - |  | - | - | - |
| vesper sparrow |  | - | - | - | 13 | 0.01 | 309 | 193 |  | - | - | - |  | - | - | - | 6 | 0.02 | 3,219 | 1,747 |
| warbling vireo |  | - | - | - | 11 | 0.01 | 262 | 163 |  | - | - | - | 6 | 0.01 | 80 | 62 | 2 | 0.01 | 1,073 | 582 |
| western bluebird |  | - | - | - | 1 | <0.01 | 24 | 15 |  | - | - | - |  | - | - | - |  | - | - | - |
| western flycatcher |  | - | - | - |  | - | - | - |  | - | - | - | 1 | <0.01 | 13 | 10 |  | - | - | - |
| western kingbird |  | - | - | - | 1 | <0.01 | 24 | 15 |  | - | - | - |  | - | - | - |  | - | - | - |
| western meadowlark |  | - | - | - | 47 | 0.04 | 1,118 | 698 |  | - | - | - | 106 | 0.23 | 1,411 | 1,100 | 6 | 0.02 | 3,219 | 1,747 |
| western scrub-jay |  | - | - | - |  | - | - | - |  | - | - | - | 12 | 0.03 | 160 | 125 |  | - | - | - |
| western tanager |  | - | - | - | 6 | <0.01 | 143 | 89 |  | - | - | - | 3 | 0.01 | 40 | 31 |  | - | - | - |
| western wood-pewee |  | - | - | - | 1 | <0.01 | 24 | 15 |  | - | - | - | 1 | <0.01 | 13 | 10 |  | - | - | - |
| white-breasted nuthatch |  | - | - | - | 1 | <0.01 | 24 | 15 | 1 | <0.01 | 19 | 15 |  | - | - | - |  | - | - | - |
| white-crowned sparrow |  | - | - | - | 32 | 0.02 | 761 | 475 | 1 | <0.01 | 19 | 15 | 3 | 0.01 | 40 | 31 | 1 | <0.01 | 537 | 291 |
| white-eyed vireo | 2 | <0.01 | 66 | 44 |  | - | - | - |  | - | - | - |  | - | - | - |  | - | - | - |
| white-throated sparrow | 1 | <0.01 | 33 | 22 |  | - | - | - | 1 | <0.01 | 19 | 15 |  | - | - | - |  | - | - | - |
| white-winged crossbill |  | - | - | - |  | - | - | - | 1 | <0.01 | 19 | 15 |  | - | - | - |  | - | - | - |
| Wilson's warbler | 1 | <0.01 | 33 | 22 | 9 | 0.01 | 214 | 134 |  | - | - | - | 16 | 0.04 | 213 | 166 | 1 | <0.01 | 537 | 291 |
| winter wren | 3 | <0.01 | 98 | 66 | 14 | 0.01 | 333 | 208 | 1 | <0.01 | 19 | 15 |  | - | - | - | 1 | <0.01 | 537 | 291 |
| wood thrush | 25 | 0.03 | 821 | 553 |  | - | - | - |  | - | - | - |  | - | - | - |  | - | - | - |
| yellow warbler | 1 | <0.01 | 33 | 22 | 1 | <0.01 | 24 | 15 | 1 | <0.01 | 19 | 15 | 7 | 0.02 | 93 | 73 | 2 | 0.01 | 1,073 | 582 |
| yellow-bellied flycatcher | 4 | 0.01 | 131 | 88 |  | - | - | - | 3 | 0.01 | 57 | 46 |  | - | - | - | 1 | <0.01 | 537 | 291 |
| yellow-breasted Chat |  | - | - | - |  | - | - | - |  | - | - | - | 1 | <0.01 | 13 | 10 |  | - | - | - |
| yellow-headed blackbird |  | - | - | - |  | - | - | - |  | - | - | - |  | - | - | - | 2 | 0.01 | 1,073 | 582 |
| yellow-rumped warbler | 12 | 0.02 | 394 | 265 | 31 | 0.02 | 737 | 460 | 2 | 0.01 | 38 | 31 | 8 | 0.02 | 106 | 83 | 4 | 0.01 | 2,146 | 1,165 |
| yellow-throated vireo | 2 | <0.01 | 66 | 44 |  | - | - | - |  | - | - | - |  | - | - | - | 2 | 0.01 | 1,073 | 582 |
| **Total** | **762** | **1.00** | **25,010** | **16,853** | **1,340** | **1.00** | **31,871** | **19,896** | **277** | **1.00** | **5,293** | **4,257** | **457** | **1.00** | **6,082** | **4,743** | **273** | **1.00** | **146,477** | **79,478** |
| *The Dillon Project was the only project in the Southwestern Biome represented by a fatality report that was available. Due to its singularity and since it is located very close to the Pacific Biome; it was combined with the Pacific Biome data for these estimates. | | | | | | | | | | | | | | | | | | | | |
