# Supplementary material for: A Comprehensive Analysis of Small-Passerine Fatalities from Collision with Turbines at Wind Energy Facilities
Source: PLoS One. 2014 Sep 15;9(9):e107491. doi: 10.1371/journal.pone.0107491 (PMC4164633; doi:10.1371/journal.pone.0107491)
Supplement: Appendix S9 — The cumulative values for analysis of small-passerine fatalities in 116 studies at wind energy facilities from all associated avifaunal biomes* in the United States and Canada. Total number and percent composition of fatalities of small-passerines, estimated number of fatalities using for the lowest and highest value of bias and the biome-specific rate (bird fatalities/megawatt/year) from studies and total megawatts of operating capacity, and the adjusted number of fatalities by species. (DOCX) [file pone.0107491.s036.docx]

**Appendix S9. The cumulative values for analysis of small-passerine fatalities in 116 studies at wind energy facilities from all associated avifaunal biomes* in the United States and Canada.** Total number and percent composition of fatalities of small-passerines, estimated number of fatalities using for the lowest and highest value of bias and the biome-specific rate (bird fatalities/megawatt/year) from studies and total megawatts of operating capacity, and the adjusted number of fatalities by species. Based on Appendix S8.

| **Species** | **Scientific Name** | **Total Fatalities** | **%Comp** | **Low Bias Across Biomes** | **High Bias Across Biomes** | **Adj Low Bias**** | **Adj High Bias**** |
| --- | --- | --- | --- | --- | --- | --- | --- |
| Acadian flycatcher | *Empidonax virescens* | 2 | 0.06 | 66 | 44 | 70 | 47 |
| alder flycatcher | *Empidonax alnorum* | 1 | 0.03 | 19 | 15 | 20 | 16 |
| American goldfinch | *Carduelis tristis* | 11 | 0.35 | 1,784 | 996 | 1,909 | 1,066 |
| American pipit | *Anthus rubescens* | 13 | 0.42 | 194 | 144 | 207 | 154 |
| American redstart | *Setophaga ruticilla* | 18 | 0.58 | 1,543 | 909 | 1,651 | 973 |
| American robin | *Turdus migratorius* | 28 | 0.90 | 1,214 | 746 | 1,299 | 798 |
| American tree sparrow | *Spizella arborea* | 7 | 0.23 | 3,756 | 2,038 | 4,019 | 2,181 |
| ash-throated flycatcher | *Myiarchus cinerascens* | 2 | 0.06 | 37 | 25 | 40 | 27 |
| bank swallow | *Riparia* | 1 | 0.03 | 33 | 22 | 35 | 24 |
| barn swallow | *Hirundo rustica* | 11 | 0.35 | 4,881 | 2,658 | 5,222 | 2,844 |
| bay-breasted warbler | *Setophaga castanea* | 18 | 0.58 | 522 | 364 | 559 | 390 |
| Bell's vireo | *Vireo bellii* | 1 | 0.03 | 537 | 291 | 574 | 312 |
| Bewick's wren | *Thryomanes bewickii* | 1 | 0.03 | 13 | 10 | 14 | 11 |
| black-and-white warbler | *Mniotilta varia* | 14 | 0.45 | 1,916 | 1,090 | 2,050 | 1,166 |
| Blackburnian warbler | *Setophaga fusca* | 10 | 0.32 | 287 | 201 | 307 | 215 |
| black-capped chickadee | *Poecile atricapilla* | 1 | 0.03 | 19 | 15 | 20 | 16 |
| black-headed grosbeak | *Pheucticus melanocephalus* | 7 | 0.23 | 93 | 72 | 99 | 78 |
| blackpoll warbler | *Setophaga striata* | 50 | 1.61 | 2,063 | 1,334 | 2,207 | 1,428 |
| black-tailed gnatcatcher | *Polioptila melanura* | 1 | 0.03 | 13 | 10 | 14 | 11 |
| black-throated blue warbler | *Setophaga caerulescens* | 27 | 0.87 | 836 | 570 | 895 | 610 |
| black-throated gray warbler | *Setophaga nigrescens* | 9 | 0.29 | 172 | 116 | 184 | 124 |
| black-throated green warbler | *Setophaga virens* | 17 | 0.55 | 1,007 | 618 | 1,077 | 661 |
| black-throated sparrow | *Amphispiza bilineata* | 4 | 0.13 | 74 | 50 | 79 | 54 |
| blue jay | *Cyanocitta cristata* | 7 | 0.23 | 692 | 404 | 741 | 432 |
| blue-headed vireo | *Vireo solitarius* | 14 | 0.45 | 377 | 269 | 404 | 288 |
| blue-winged warbler | *Vermivora cyanoptera* | 2 | 0.06 | 66 | 44 | 70 | 47 |
| bobolink | *Dolichonyx oryzivorus* | 22 | 0.71 | 654 | 453 | 699 | 485 |
| Brewer's blackbird | *Euphagus cyanocephalus* | 24 | 0.77 | 1,386 | 819 | 1,483 | 876 |
| Brewer's sparrow | *Spizella breweri* | 17 | 0.55 | 404 | 252 | 433 | 270 |
| brown creeper | *Certhia americana* | 10 | 0.32 | 1,267 | 717 | 1,356 | 767 |
| brown thrasher | *Toxostoma rufum* | 3 | 0.10 | 1,610 | 873 | 1,722 | 935 |
| brown-headed cowbird | *Molothrus ater* | 5 | 0.16 | 2,170 | 1,179 | 2,322 | 1,262 |
| Bullock's oriole | *Icterus bullockii* | 2 | 0.06 | 27 | 21 | 28 | 22 |
| bushtit | *Psaltriparus minimus* | 1 | 0.03 | 537 | 291 | 574 | 312 |
| Canada warbler | *Cardellina canadensis* | 6 | 0.19 | 197 | 133 | 211 | 142 |
| Cape May warbler | *Setophaga tigrina* | 13 | 0.42 | 930 | 557 | 996 | 595 |
| Cassin's vireo | *Vireo cassinii* | 5 | 0.16 | 108 | 70 | 116 | 75 |
| cedar waxwing | *Bombycilla cedrorum* | 15 | 0.48 | 1,885 | 1,078 | 2,017 | 1,153 |
| cerulean warbler | *Setophaga cerulea* | 1 | 0.03 | 33 | 22 | 35 | 24 |
| chestnut-collared longspur | *Calcarius ornatus* | 2 | 0.06 | 48 | 30 | 51 | 32 |
| chestnut-sided warbler | *Setophaga pensylvanica* | 18 | 0.58 | 1,585 | 929 | 1,695 | 994 |
| chipping sparrow | *Spizella passerina* | 11 | 0.35 | 251 | 159 | 269 | 170 |
| cliff swallow | *Petrochelidon pyrrhonota* | 13 | 0.42 | 1,861 | 1,037 | 1,991 | 1,110 |
| common grackle | *Quiscalus quiscula* | 5 | 0.16 | 2,165 | 1,180 | 2,317 | 1,262 |
| common yellowthroat | *Geothlypis trichas* | 26 | 0.84 | 4,785 | 2,669 | 5,120 | 2,856 |
| dark-eyed junco | *Junco hyemalis* | 53 | 1.70 | 6,884 | 3,825 | 7,366 | 4,093 |
| dickcissel | *Spiza americana* | 3 | 0.10 | 1,610 | 873 | 1,722 | 935 |
| eastern bluebird | *Sialia sialis* | 3 | 0.10 | 98 | 66 | 105 | 71 |
| eastern kingbird | *Tyrannus tyrannus* | 15 | 0.48 | 2,398 | 1,354 | 2,565 | 1,449 |
| eastern meadowlark | *Sturnella magna* | 3 | 0.10 | 1,610 | 873 | 1,722 | 935 |
| eastern phoebe | *Sayornis phoebe* | 1 | 0.03 | 33 | 22 | 35 | 24 |
| eastern towhee | *Pipilo erythrophthalmus* | 5 | 0.16 | 150 | 104 | 161 | 111 |
| eastern wood-pewee | *Contopus virens* | 4 | 0.13 | 90 | 68 | 96 | 73 |
| European starling | *Sturnus vulgaris* | 103 | 3.31 | 7,376 | 4,265 | 7,892 | 4,563 |
| evening grosbeak | *Coccothraustes vespertinus* | 1 | 0.03 | 19 | 15 | 20 | 16 |
| field sparrow | *Spizella pusilla* | 7 | 0.23 | 1,237 | 693 | 1,324 | 741 |
| fox sparrow | *Passerella iliaca* | 1 | 0.03 | 537 | 291 | 574 | 312 |
| golden-crowned kinglet | *Regulus satrapa* | 158 | 5.08 | 10,150 | 5,943 | 10,861 | 6,359 |
| golden-crowned sparrow | *Zonotrichia atricapilla* | 6 | 0.19 | 132 | 85 | 141 | 91 |
| grasshopper sparrow | *Ammodramus savannarum* | 5 | 0.16 | 2,170 | 1,179 | 2,322 | 1,262 |
| gray catbird | *Dumetella carolinensis* | 11 | 0.35 | 1,849 | 1,036 | 1,979 | 1,109 |
| gray flycatcher | *Empidonax wrightii* | 2 | 0.06 | 48 | 30 | 51 | 32 |
| gray vireo | *Vireo vicinior* | 1 | 0.03 | 24 | 15 | 25 | 16 |
| gray-cheeked thrush | *Catharus minimus* | 8 | 0.26 | 766 | 446 | 820 | 477 |
| green-tailed towhee | *Pipilo chlorurus* | 4 | 0.13 | 95 | 59 | 102 | 64 |
| Hammond's flycatcher | *Empidonax hammondii* | 5 | 0.16 | 98 | 65 | 105 | 70 |
| hermit thrush | *Catharus guttatus* | 10 | 0.32 | 254 | 174 | 272 | 186 |
| hooded warbler | *Setophaga citrina* | 4 | 0.13 | 131 | 88 | 140 | 95 |
| horned lark | *Eremophila alpestris* | 681 | 21.90 | 28,590 | 16,850 | 30,591 | 18,029 |
| house finch | *Haemorhous mexicanus* | 8 | 0.26 | 159 | 105 | 170 | 113 |
| house sparrow | *Passer domesticus* | 15 | 0.48 | 886 | 516 | 948 | 552 |
| house wren | *Troglodytes aedon* | 20 | 0.64 | 3,542 | 1,950 | 3,790 | 2,087 |
| indigo bunting | *Passerina cyanea* | 3 | 0.10 | 71 | 53 | 76 | 57 |
| Kentucky warbler | *Geothlypis formosa* | 2 | 0.06 | 66 | 44 | 70 | 47 |
| Lapland longspur | *Calcarius lapponicus* | 2 | 0.06 | 1,073 | 582 | 1,148 | 623 |
| lark bunting | *Calamospiza melanocorys* | 1 | 0.03 | 24 | 15 | 25 | 16 |
| lark sparrow | *Chondestes grammacus* | 2 | 0.06 | 27 | 21 | 28 | 22 |
| Le Conte's sparrow | *Ammodramus leconteii* | 2 | 0.06 | 1,073 | 582 | 1,148 | 623 |
| least flycatcher | *Empidonax minimus* | 2 | 0.06 | 556 | 306 | 595 | 328 |
| lesser goldfinch | *Carduelis psaltria* | 1 | 0.03 | 13 | 10 | 14 | 11 |
| Lincoln's sparrow | *Melospiza lincolnii* | 15 | 0.48 | 855 | 503 | 914 | 538 |
| loggerhead shrike | *Lanius ludovicianus* | 5 | 0.16 | 590 | 333 | 631 | 356 |
| MacGillivray's warbler | *Geothlypis tolmiei* | 8 | 0.26 | 159 | 105 | 170 | 113 |
| magnolia warbler | *Setophaga magnolia* | 60 | 1.93 | 3,737 | 2,282 | 3,999 | 2,441 |
| marsh wren | *Cistothorus palustris* | 2 | 0.06 | 1,073 | 582 | 1,148 | 623 |
| mountain bluebird | *Sialia currucoides* | 6 | 0.19 | 143 | 89 | 153 | 95 |
| mourning warbler | *Geothlypis philadelphia* | 1 | 0.03 | 33 | 22 | 35 | 24 |
| Nashville warbler | *Oreothlypis ruficapilla* | 2 | 0.06 | 32 | 26 | 35 | 28 |
| northern mockingbird | *Mimus polyglottos* | 6 | 0.19 | 630 | 357 | 674 | 382 |
| northern Parula | *Setophaga americana* | 6 | 0.19 | 156 | 112 | 167 | 120 |
| northern rough-winged swallow | *Stelgidopteryx serripennis* | 3 | 0.10 | 584 | 321 | 625 | 343 |
| northern shrike | *Lanius excubitor* | 1 | 0.03 | 24 | 15 | 25 | 16 |
| northern waterthrush | *Parkesia noveboracensis* | 3 | 0.10 | 85 | 60 | 91 | 64 |
| oak titmouse | *Baeolophus inornatus* | 1 | 0.03 | 13 | 10 | 14 | 11 |
| orange-crowned warbler | *Oreothlypis celata* | 15 | 0.48 | 3,381 | 1,858 | 3,617 | 1,988 |
| orchard oriole | *Icterus spurius* | 1 | 0.03 | 537 | 291 | 574 | 312 |
| ovenbird | *Seiurus aurocapilla* | 22 | 0.71 | 654 | 453 | 699 | 485 |
| Pacific-slope flycatcher | *Empidonax difficilis* | 4 | 0.13 | 64 | 46 | 68 | 49 |
| palm warbler | *Setophaga palmarum* | 4 | 0.13 | 131 | 88 | 140 | 95 |
| Philadelphia vireo | *Vireo philadelphicus* | 4 | 0.13 | 118 | 82 | 126 | 87 |
| pine siskin | *Carduelis pinus* | 3 | 0.10 | 80 | 52 | 86 | 55 |
| pine warbler | *Setophaga pinus* | 2 | 0.06 | 38 | 31 | 41 | 33 |
| prairie warbler | *Setophaga discolor* | 1 | 0.03 | 19 | 15 | 20 | 16 |
| purple finch | *Haemorhous purpureus* | 4 | 0.13 | 81 | 61 | 87 | 65 |
| purple martin | *Progne subis* | 3 | 0.10 | 1,106 | 604 | 1,183 | 647 |
| red crossbill | *Loxia curvirostra* | 1 | 0.03 | 19 | 15 | 20 | 16 |
| red-breasted nuthatch | *Sitta canadensis* | 20 | 0.64 | 475 | 314 | 508 | 336 |
| red-eyed vireo | *Vireo olivaceus* | 265 | 8.52 | 9,496 | 6,317 | 10,161 | 6,759 |
| red-winged blackbird | *Agelaius phoeniceus* | 70 | 2.25 | 4,613 | 2,699 | 4,936 | 2,888 |
| rock wren | *Salpinctes obsoletus* | 14 | 0.45 | 291 | 190 | 311 | 203 |
| rose-breasted grosbeak | *Pheucticus ludovicianus* | 9 | 0.29 | 772 | 455 | 826 | 486 |
| ruby-crowned kinglet | *Regulus calendula* | 55 | 1.77 | 5,418 | 3,075 | 5,798 | 3,290 |
| sage sparrow | *Artemisiospiza belli* | 1 | 0.03 | 24 | 15 | 25 | 16 |
| sage thrasher | *Oreoscoptes montanus* | 3 | 0.10 | 71 | 45 | 76 | 48 |
| savannah sparrow | *Passerculus sandwichensis* | 37 | 1.19 | 4,414 | 2,483 | 4,723 | 2,656 |
| Say's phoebe | *Sayornis saya* | 2 | 0.06 | 37 | 25 | 40 | 27 |
| scarlet tanager | *Piranga olivacea* | 4 | 0.13 | 118 | 82 | 126 | 87 |
| scissor-tailed flycatcher | *Tyrannus forficatus* | 1 | 0.03 | 537 | 291 | 574 | 312 |
| sedge wren | *Cistothorus platensis* | 3 | 0.10 | 1,610 | 873 | 1,722 | 935 |
| snow bunting | *Plectrophenax nivalis* | 1 | 0.03 | 537 | 291 | 574 | 312 |
| song sparrow | *Melospiza melodia* | 10 | 0.32 | 1,776 | 986 | 1,900 | 1,055 |
| spotted towhee | *Pipilo maculatus* | 7 | 0.23 | 669 | 376 | 716 | 402 |
| Steller's jay | *Cyanocitta stelleri* | 1 | 0.03 | 24 | 15 | 25 | 16 |
| Swainson's thrush | *Catharus ustulatus* | 18 | 0.58 | 535 | 366 | 572 | 391 |
| swamp sparrow | *Melospiza georgiana* | 3 | 0.10 | 1,106 | 604 | 1,183 | 647 |
| Tennessee warbler | *Oreothlypis peregrina* | 10 | 0.32 | 818 | 483 | 876 | 517 |
| Townsend's solitaire | *Myadestes townsendi* | 2 | 0.06 | 48 | 30 | 51 | 32 |
| Townsend's warbler | *Setophaga townsendi* | 38 | 1.22 | 841 | 537 | 900 | 575 |
| tree swallow | *Tachycineta bicolor* | 34 | 1.09 | 6,907 | 3,834 | 7,390 | 4,102 |
| tricolored blackbird | *Agelaius tricolor* | 2 | 0.06 | 27 | 21 | 28 | 22 |
| tufted titmouse | *Baeolophus bicolor* | 1 | 0.03 | 33 | 22 | 35 | 24 |
| unidentified blackbird |  | 6 | 0.19 | 140 | 95 | 150 | 101 |
| unidentified bluebird |  | 2 | 0.06 | 27 | 21 | 28 | 22 |
| unidentified corvid |  | 5 | 0.16 | 155 | 103 | 166 | 111 |
| unidentified crowned sparrow |  | 4 | 0.13 | 53 | 41 | 57 | 44 |
| unidentified empidonax |  | 10 | 0.32 | 1,279 | 721 | 1,368 | 772 |
| unidentified flycatcher |  | 11 | 0.35 | 810 | 485 | 867 | 519 |
| unidentified kingbird |  | 5 | 0.16 | 105 | 76 | 112 | 81 |
| unidentified kinglet |  | 8 | 0.26 | 186 | 119 | 199 | 128 |
| unidentified meadowlark |  | 1 | 0.03 | 537 | 291 | 574 | 312 |
| unidentified nuthatch |  | 1 | 0.03 | 19 | 15 | 20 | 16 |
| unidentified passerine |  | 120 | 3.86 | 7,988 | 4,654 | 8,547 | 4,979 |
| unidentified sparrow |  | 25 | 0.80 | 3,131 | 1,747 | 3,350 | 1,869 |
| unidentified swallow |  | 4 | 0.13 | 597 | 331 | 639 | 354 |
| unidentified thrasher |  | 2 | 0.06 | 27 | 21 | 28 | 22 |
| unidentified thrush |  | 6 | 0.19 | 146 | 100 | 156 | 107 |
| unidentified vireo |  | 7 | 0.23 | 194 | 126 | 207 | 135 |
| unidentified warbler |  | 34 | 1.09 | 2,364 | 1,389 | 2,530 | 1,486 |
| unidentified wren |  | 2 | 0.06 | 43 | 30 | 46 | 32 |
| varied thrush | *Ixoreus naevius* | 4 | 0.13 | 85 | 55 | 91 | 59 |
| veery | *Catharus fuscescens* | 9 | 0.29 | 282 | 192 | 301 | 206 |
| vesper sparrow | *Pooecetes gramineus* | 19 | 0.61 | 3,528 | 1,940 | 3,775 | 2,076 |
| warbling vireo | *Vireo gilvus* | 19 | 0.61 | 1,414 | 808 | 1,513 | 864 |
| western bluebird | *Sialia mexicana* | 1 | 0.03 | 24 | 15 | 25 | 16 |
| western flycatcher | *Empidonax difficilis* | 1 | 0.03 | 13 | 10 | 14 | 11 |
| western kingbird | *Tyrannus verticalis* | 1 | 0.03 | 24 | 15 | 25 | 16 |
| western meadowlark | *Sturnella neglecta* | 159 | 5.11 | 5,745 | 3,542 | 6,147 | 3,790 |
| western scrub-jay | *Aphelocoma californica* | 12 | 0.39 | 159 | 124 | 171 | 133 |
| western tanager | *Piranga ludoviciana* | 9 | 0.29 | 183 | 120 | 195 | 129 |
| western wood-pewee | *Contopus sordidulus* | 2 | 0.06 | 37 | 25 | 40 | 27 |
| white-breasted nuthatch | *Sitta carolinensis* | 2 | 0.06 | 43 | 30 | 46 | 32 |
| white-crowned sparrow | *Zonotrichia leucophrys* | 37 | 1.19 | 1,357 | 813 | 1,452 | 870 |
| white-eyed vireo | *Vireo griseus* | 2 | 0.06 | 66 | 44 | 70 | 47 |
| white-throated sparrow | *Zonotrichia albicollis* | 2 | 0.06 | 52 | 37 | 56 | 40 |
| white-winged crossbill | *Loxia leucoptera* | 1 | 0.03 | 19 | 15 | 20 | 16 |
| Wilson's warbler | *Cardellina pusilla* | 27 | 0.87 | 996 | 613 | 1,066 | 655 |
| winter wren | *Troglodytes hiemalis* | 19 | 0.61 | 987 | 581 | 1,056 | 621 |
| wood thrush | *Hylocichla mustelina* | 25 | 0.80 | 821 | 553 | 878 | 592 |
| yellow warbler | *Setophaga petechia* | 12 | 0.39 | 1,242 | 707 | 1,329 | 757 |
| yellow-bellied flycatcher | *Empidonax flaviventris* | 8 | 0.26 | 725 | 426 | 776 | 455 |
| yellow-breasted chat | *Icteria virens* | 1 | 0.03 | 13 | 10 | 14 | 11 |
| yellow-headed blackbird | *Xanthocephalus xanthocephalus* | 2 | 0.06 | 1,073 | 582 | 1,148 | 623 |
| yellow-rumped warbler | *Setophaga coronata* | 57 | 1.83 | 3,422 | 2,004 | 3,661 | 2,144 |
| yellow-throated vireo | *Vireo flavifrons* | 4 | 0.13 | 1,139 | 626 | 1,218 | 670 |
| **Total** | **156 species** | **3,110** | **100** | **214,733** | **125,227** | **229,765** | **133,993** |
| *The eastern, intermountain west, northern forest, Pacific, and prairie biomes are included.  **To account for the absence of data from the Southwestern biome, which comprises 7% of the total operating capacity for wind energy in the US and Canada, estimates were increased by 7%. | | | | | | | |
