# Supplementary material for: A Comprehensive Analysis of Small-Passerine Fatalities from Collision with Turbines at Wind Energy Facilities
Source: PLoS One. 2014 Sep 15;9(9):e107491. doi: 10.1371/journal.pone.0107491 (PMC4164633; doi:10.1371/journal.pone.0107491)
Supplement: Appendix S10 — Annual fatality rates compared to population sizes for species of small passerines found as fatalities in 116 available studies conducted at wind energy facilities in the United States and Canada. The estimated average number and percent of population killed each year after low and high bias adjustments were applied and adjusted for operating capacity (see Appendix S9); estimated population size, and the proportion of the population that these estimates represent. Based on actual fatalities found both during scheduled searches and incidentally. (DOCX) [file pone.0107491.s037.docx]

**Appendix S10. Annual fatality rates compared to population sizes for species of small passerines found as fatalities in 116 available studies conducted at wind energy facilities in the United States and Canada.** The estimated average number and percent of population killed each year after low and high bias adjustments were applied and adjusted for operating capacity (see Appendix S9); estimated population size, and the proportion of the population that these estimates represent. Based on actual fatalities found both during scheduled searches and incidentally.

| **Species** | **Scientific Name** | **Ave est (low bias) ^a^** | **Ave est (high bias) ^b^** | **Pop est for North America ^c^** | **% pop affected (low bias) ^d^** | **% pop affected (high bias)^e^** |
| --- | --- | --- | --- | --- | --- | --- |
| Acadian flycatcher | *Empidonax virescens* | 70 | 47 | 4,500,000 | 0.002 | 0.001 |
| alder flycatcher | *Empidonax alnorum* | 20 | 16 | 130,000,000 | <0.001 | <0.001 |
| American goldfinch | *Carduelis tristis* | 1790 | 1020 | 42,000,000 | 0.005 | 0.003 |
| American pipit | *Anthus rubescens* | 207 | 154 | 18,000,000 | 0.001 | 0.001 |
| American redstart | *Setophaga ruticilla* | 1572 | 942 | 39,000,000 | 0.004 | 0.002 |
| American robin | *Turdus migratorius* | 1259 | 783 | 300,000,000 | <0.001 | <0.001 |
| American tree sparrow | *Spizella arborea* | 3740 | 2072 | 20,000,000 | 0.020 | 0.011 |
| ash-throated flycatcher | *Myiarchus cinerascens* | 40 | 27 | 5,200,000 | 0.001 | 0.001 |
| bank swallow | *Riparia riparia* | 35 | 24 | 6,000,000 | 0.001 | <0.001 |
| barn swallow | *Hirundo rustica* | 4864 | 2705 | 33,000,000 | 0.016 | 0.009 |
| bay-breasted warbler | *Setophaga castanea* | 559 | 390 | 9,000,000 | 0.006 | 0.004 |
| Bell's vireo | *Vireo bellii* | 534 | 296 | 3,600,000 | 0.016 | 0.009 |
| Bewick's wren | *Thryomanes bewickii* | 14 | 11 | 4,000,000 | <0.001 | <0.001 |
| black-and-white warbler | *Mniotilta varia* | 1931 | 1120 | 20,000,000 | 0.010 | 0.006 |
| Blackburnian warbler | *Setophaga fusca* | 307 | 215 | 10,000,000 | 0.003 | 0.002 |
| black-capped chickadee | *Poecile atricapilla* | 20 | 16 | 41,000,000 | <0.001 | <0.001 |
| black-headed grosbeak | *Pheucticus melanocephalus* | 99 | 78 | 12,000,000 | 0.001 | 0.001 |
| blackpoll warbler | *Setophaga striata* | 2167 | 1412 | 60,000,000 | 0.004 | 0.002 |
| black-tailed gnatcatcher | *Polioptila melanura* | 14 | 11 | 4,000,000 | <0.001 | <0.001 |
| black-throated blue warbler | *Setophaga caerulescens* | 895 | 610 | 2,100,000 | 0.043 | 0.029 |
| black-throated gray warbler | *Setophaga nigrescens* | 184 | 124 | 2,400,000 | 0.008 | 0.005 |
| black-throated green warbler | *Setophaga virens* | 1038 | 646 | 10,000,000 | 0.011 | 0.007 |
| black-throated sparrow | *Amphispiza bilineata* | 79 | 54 | 29,000,000 | <0.001 | <0.001 |
| blue jay | *Cyanocitta cristata* | 701 | 416 | 13,000,000 | 0.006 | 0.003 |
| blue-headed vireo | *Vireo solitarius* | 404 | 288 | 9,000,000 | 0.004 | 0.003 |
| blue-winged warbler | *Vermivora cyanoptera* | 70 | 47 | 810,000 | 0.009 | 0.006 |
| bobolink | *Dolichonyx oryzivorus* | 699 | 485 | 8,000,000 | 0.009 | 0.006 |
| Brewer's blackbird | *Euphagus cyanocephalus* | 1404 | 845 | 20,000,000 | 0.007 | 0.004 |
| Brewer's sparrow | *Spizella breweri* | 433 | 270 | 13,000,000 | 0.003 | 0.002 |
| brown creeper | *Certhia americana* | 1277 | 736 | 8,500,000 | 0.016 | 0.009 |
| brown thrasher | *Toxostoma rufum* | 1603 | 888 | 4,900,000 | 0.035 | 0.019 |
| brown-headed cowbird | *Molothrus ater* | 2163 | 1200 | 110,000,000 | 0.002 | 0.001 |
| Bullock's oriole | *Icterus bullockii* | 28 | 22 | 6,200,000 | <0.001 | <0.001 |
| bushtit | *Psaltriparus minimus* | 534 | 296 | 2,300,000 | 0.025 | 0.014 |
| Canada warbler | *Cardellina canadensis* | 211 | 142 | 4,000,000 | 0.005 | 0.004 |
| Cape May warbler | *Setophaga tigrina* | 956 | 580 | 7,000,000 | 0.014 | 0.009 |
| Cassin's vireo | *Vireo cassinii* | 116 | 75 | 4,000,000 | 0.003 | 0.002 |
| cedar waxwing | *Bombycilla cedrorum* | 1897 | 1107 | 52,000,000 | 0.004 | 0.002 |
| cerulean warbler | *Setophaga cerulea* | 35 | 24 | 600,000 | 0.006 | 0.004 |
| chestnut-collared longspur | *Calcarius ornatus* | 51 | 32 | 3,000,000 | 0.002 | 0.001 |
| chestnut-sided warbler | *Setophaga pensylvanica* | 1616 | 964 | 19,000,000 | 0.009 | 0.005 |
| chipping sparrow | *Spizella passerina* | 269 | 170 | 210,000,000 | <0.001 | <0.001 |
| cliff swallow | *Petrochelidon pyrrhonota* | 1872 | 1063 | 40,000,000 | 0.005 | 0.003 |
| common grackle | *Quiscalus quiscula* | 2158 | 1201 | 61,000,000 | 0.004 | 0.002 |
| common yellowthroat | *Geothlypis trichas* | 4802 | 2732 | 83,000,000 | 0.006 | 0.003 |
| dark-eyed junco | *Junco hyemalis* | 6928 | 3922 | 200,000,000 | 0.004 | 0.002 |
| dickcissel | *Spiza americana* | 1603 | 888 | 20,000,000 | 0.009 | 0.005 |
| eastern bluebird | *Sialia sialis* | 105 | 71 | 19,000,000 | 0.001 | <0.001 |
| eastern kingbird | *Tyrannus tyrannus* | 2406 | 1387 | 27,000,000 | 0.010 | 0.005 |
| eastern meadowlark | *Sturnella magna* | 1603 | 888 | 22,000,000 | 0.008 | 0.004 |
| eastern phoebe | *Sayornis phoebe* | 35 | 24 | 32,000,000 | <0.001 | <0.001 |
| eastern towhee | *Pipilo erythrophthalmus* | 161 | 111 | 28,000,000 | 0.001 | <0.001 |
| eastern wood-pewee | *Contopus virens* | 96 | 73 | 5,500,000 | 0.002 | 0.001 |
| European starling | *Sturnus vulgaris* | 7494 | 4409 | 57,000,000 | 0.014 | 0.008 |
| evening grosbeak | *Coccothraustes vespertinus* | 20 | 16 | 3,900,000 | 0.001 | <0.001 |
| field sparrow | *Spizella pusilla* | 1244 | 710 | 7,600,000 | 0.017 | 0.010 |
| fox sparrow | *Passerella iliaca* | 534 | 296 | 20,000,000 | 0.003 | 0.002 |
| golden-crowned kinglet | *Regulus satrapa* | 10384 | 6174 | 96,000,000 | 0.011 | 0.007 |
| golden-crowned sparrow | *Zonotrichia atricapilla* | 141 | 91 | 4,000,000 | 0.004 | 0.002 |
| grasshopper sparrow | *Ammodramus savannarum* | 2163 | 1200 | 14,000,000 | 0.017 | 0.009 |
| gray catbird | *Dumetella carolinensis* | 1860 | 1062 | 27,000,000 | 0.007 | 0.004 |
| gray flycatcher | *Empidonax wrightii* | 51 | 32 | 3,000,000 | 0.002 | 0.001 |
| gray vireo | *Vireo vicinior* | 25 | 16 | 400,000 | 0.006 | 0.004 |
| gray-cheeked thrush | *Catharus minimus* | 780 | 462 | 15,000,000 | 0.005 | 0.003 |
| green-tailed towhee | *Pipilo chlorurus* | 102 | 64 | 4,100,000 | 0.002 | 0.002 |
| Hammond's flycatcher | *Empidonax hammondii* | 105 | 70 | 19,000,000 | 0.001 | <0.001 |
| hermit thrush | *Catharus guttatus* | 272 | 186 | 40,000,000 | 0.001 | <0.001 |
| hooded warbler | *Setophaga citrina* | 140 | 95 | 4,000,000 | 0.004 | 0.002 |
| horned lark | *Eremophila alpestris* | 29597 | 17643 | 80,000,000 | 0.038 | 0.023 |
| house finch | *Haemorhous mexicanus* | 170 | 113 | 35,000,000 | <0.001 | <0.001 |
| house sparrow | *Passer domesticus* | 908 | 537 | 82,000,000 | 0.001 | 0.001 |
| house wren | *Troglodytes aedon* | 3551 | 1994 | 42,000,000 | 0.009 | 0.005 |
| indigo bunting | *Passerina cyanea* | 76 | 57 | 78,000,000 | <0.001 | <0.001 |
| Kentucky warbler | *Geothlypis formosa* | 70 | 47 | 1,100,000 | 0.006 | 0.004 |
| Lapland longspur | *Calcarius lapponicus* | 1069 | 592 | 60,000,000 | 0.002 | 0.001 |
| lark bunting | *Calamospiza melanocorys* | 25 | 16 | 9,100,000 | <0.001 | <0.001 |
| lark sparrow | *Chondestes grammacus* | 28 | 22 | 6,900,000 | <0.001 | <0.001 |
| Le Conte's sparrow | *Ammodramus leconteii* | 1069 | 592 | 8,000,000 | 0.014 | 0.008 |
| least flycatcher | *Empidonax minimus* | 555 | 312 | 36,000,000 | 0.002 | 0.001 |
| lesser goldfinch | *Carduelis psaltria* | 14 | 11 | 3,900,000 | <0.001 | <0.001 |
| Lincoln's sparrow | *Melospiza lincolnii* | 875 | 523 | 70,000,000 | 0.001 | 0.001 |
| loggerhead shrike | *Lanius ludovicianus* | 591 | 340 | 4,900,000 | 0.013 | 0.007 |
| MacGillivray's warbler | *Geothlypis tolmiei* | 170 | 113 | 12,000,000 | 0.001 | 0.001 |
| magnolia warbler | *Setophaga magnolia* | 3840 | 2379 | 40,000,000 | 0.010 | 0.006 |
| marsh wren | *Cistothorus palustris* | 1069 | 592 | 9,000,000 | 0.013 | 0.007 |
| mountain bluebird | *Sialia currucoides* | 153 | 95 | 4,600,000 | 0.003 | 0.002 |
| mourning warbler | *Geothlypis philadelphia* | 35 | 24 | 17,000,000 | <0.001 | <0.001 |
| Nashville warbler | *Oreothlypis ruficapilla* | 35 | 28 | 32,000,000 | <0.001 | <0.001 |
| northern mockingbird | *Mimus polyglottos* | 634 | 366 | 27,000,000 | 0.002 | 0.001 |
| northern Parula | *Setophaga americana* | 167 | 120 | 13,000,000 | 0.001 | 0.001 |
| northern rough-winged swallow | *Stelgidopteryx serripennis* | 585 | 328 | 14,000,000 | 0.004 | 0.002 |
| northern shrike | *Lanius excubitor* | 25 | 16 | 2,000,000^f^ | 0.001 | 0.001 |
| northern waterthrush | *Parkesia noveboracensis* | 91 | 64 | 19,000,000 | <0.001 | <0.001 |
| oak titmouse | *Baeolophus inornatus* | 14 | 11 | 500,000 | 0.003 | 0.002 |
| orange-crowned warbler | *Oreothlypis celata* | 3379 | 1895 | 80,000,000 | 0.005 | 0.002 |
| orchard oriole | *Icterus spurius* | 534 | 296 | 9,200,000 | 0.006 | 0.003 |
| ovenbird | *Seiurus aurocapilla* | 699 | 485 | 22,000,000 | 0.003 | 0.002 |
| Pacific-slope flycatcher | *Empidonax difficilis* | 68 | 49 | 7,300,000 | 0.001 | 0.001 |
| palm warbler | *Setophaga palmarum* | 140 | 95 | 13,000,000 | 0.001 | 0.001 |
| Philadelphia vireo | *Vireo philadelphicus* | 126 | 87 | 4,000,000 | 0.003 | 0.002 |
| pine siskin | *Carduelis pinus* | 86 | 55 | 40,000,000 | <0.001 | <0.001 |
| pine warbler | *Setophaga pinus* | 41 | 33 | 12,000,000 | <0.001 | <0.001 |
| prairie warbler | *Setophaga discolor* | 20 | 16 | 3,500,000 | 0.001 | <0.001 |
| purple finch | *Haemorhous purpureus* | 87 | 65 | 6,300,000 | 0.001 | 0.001 |
| purple martin | *Progne subis* | 1104 | 616 | 6,000,000 | 0.020 | 0.011 |
| red crossbill | *Loxia curvirostra* | 20 | 16 | 8,000,000 | <0.001 | <0.001 |
| red-breasted nuthatch | *Sitta canadensis* | 508 | 336 | 20,000,000 | 0.003 | 0.002 |
| red-eyed vireo | *Vireo olivaceus* | 10041 | 6713 | 130,000,000 | 0.008 | 0.005 |
| red-winged blackbird | *Agelaius phoeniceus* | 4658 | 2780 | 120,000,000 | 0.004 | 0.002 |
| rock wren | *Salpinctes obsoletus* | 311 | 203 | 2,800,000 | 0.011 | 0.007 |
| rose-breasted grosbeak | *Pheucticus ludovicianus* | 786 | 471 | 4,100,000 | 0.020 | 0.012 |
| ruby-crowned kinglet | *Regulus calendula* | 5479 | 3167 | 90,000,000 | 0.006 | 0.004 |
| sage sparrow^g^ | *Artemisiospiza belli* | 25 | 16 | 4,000,000 | 0.001 | <0.001 |
| sage thrasher | *Oreoscoptes montanus* | 76 | 48 | 5,900,000 | 0.001 | 0.001 |
| savannah sparrow | *Passerculus sandwichensis* | 4444 | 2548 | 170,000,000 | 0.003 | 0.002 |
| Say's phoebe | *Sayornis saya* | 40 | 27 | 4,000,000 | 0.001 | 0.001 |
| scarlet tanager | *Piranga olivacea* | 126 | 87 | 2,200,000 | 0.006 | 0.004 |
| scissor-tailed flycatcher | *Tyrannus forficatus* | 534 | 296 | 8,700,000 | 0.007 | 0.004 |
| sedge wren | *Cistothorus platensis* | 1603 | 888 | 6,200,000 | 0.028 | 0.015 |
| snow bunting | *Plectrophenax nivalis* | 534 | 296 | 130,000,000 | <0.001 | <0.001 |
| song sparrow | *Melospiza melodia* | 1781 | 1008 | 30,000,000 | 0.006 | 0.004 |
| spotted towhee | *Pipilo maculatus* | 676 | 387 | 2,200,000 | 0.033 | 0.018 |
| Steller's jay | *Cyanocitta stelleri* | 25 | 16 | 100,000,000 | <0.001 | <0.001 |
| Swainson's thrush | *Catharus ustulatus* | 572 | 391 | 30,000,000 | 0.002 | 0.001 |
| swamp sparrow | *Melospiza georgiana* | 1104 | 616 | 70,000,000 | 0.002 | 0.001 |
| Tennessee warbler | *Oreothlypis peregrina* | 836 | 502 | 70,000,000 | 0.001 | 0.001 |
| Townsend's solitaire | *Myadestes townsendi* | 51 | 32 | 970,000 | 0.005 | 0.003 |
| Townsend's warbler | *Setophaga townsendi* | 900 | 575 | 17,000,000 | 0.005 | 0.003 |
| tree swallow | *Tachycineta bicolor* | 6913 | 3917 | 17,000,000 | 0.043 | 0.024 |
| tricolored blackbird | *Agelaius tricolor* | 28 | 22 | 300,000^f^ | 0.009 | 0.007 |
| tufted titmouse | *Baeolophus bicolor* | 35 | 24 | 8,000,000 | <0.001 | <0.001 |
| unidentified blackbird |  | 150 | 101 |  |  |  |
| unidentified bluebird |  | 28 | 22 |  |  |  |
| unidentified corvid |  | 166 | 111 |  |  |  |
| unidentified crowned sparrow |  | 57 | 44 |  |  |  |
| unidentified empidonax |  | 1288 | 741 |  |  |  |
| unidentified flycatcher |  | 827 | 504 |  |  |  |
| unidentified kingbird |  | 112 | 81 |  |  |  |
| unidentified kinglet |  | 199 | 128 |  |  |  |
| unidentified meadowlark |  | 534 | 296 |  |  |  |
| unidentified nuthatch |  | 20 | 16 |  |  |  |
| unidentified passerine |  | 8150 | 4825 |  |  |  |
| unidentified sparrow |  | 3152 | 1792 |  |  |  |
| unidentified swallow |  | 599 | 339 |  |  |  |
| unidentified thrasher |  | 28 | 22 |  |  |  |
| unidentified thrush |  | 156 | 107 |  |  |  |
| unidentified vireo |  | 207 | 135 |  |  |  |
| unidentified warbler |  | 2410 | 1440 |  |  |  |
| unidentified wren |  | 46 | 32 |  |  |  |
| varied thrush | *Ixoreus naevius* | 91 | 59 | 20,000,000 | <0.001 | <0.001 |
| veery | *Catharus fuscescens* | 301 | 206 | 11,000,000 | 0.003 | 0.002 |
| vesper sparrow | *Pooecetes gramineus* | 3537 | 1983 | 28,000,000 | 0.013 | 0.007 |
| warbling vireo | *Vireo gilvus* | 1434 | 833 | 49,000,000 | 0.003 | 0.002 |
| western bluebird | *Sialia mexicana* | 25 | 16 | 4,500,000 | 0.001 | <0.001 |
| western flycatcher | *Empidonax difficilis* | 14 | 11 | 9,300,000^h^ | <0.001 | <0.001 |
| western kingbird | *Tyrannus verticalis* | 25 | 16 | 21,000,000 | <0.001 | <0.001 |
| western meadowlark | *Sturnella neglecta* | 5908 | 3698 | 30,000,000 | 0.020 | 0.013 |
| western scrub-jay | *Aphelocoma californica* | 171 | 133 | 1,500,000 | 0.011 | 0.009 |
| western tanager | *Piranga ludoviciana* | 195 | 129 | 11,000,000 | 0.002 | 0.001 |
| western wood-pewee | *Contopus sordidulus* | 40 | 27 | 8,000,000 | <0.001 | <0.001 |
| white-breasted nuthatch | *Sitta carolinensis* | 46 | 32 | 8,300,000 | 0.001 | <0.001 |
| white-crowned sparrow | *Zonotrichia leucophrys* | 1412 | 854 | 60,000,000 | 0.002 | 0.001 |
| white-eyed vireo | *Vireo griseus* | 70 | 47 | 18,000,000 | <0.001 | <0.001 |
| white-throated sparrow | *Zonotrichia albicollis* | 56 | 40 | 140,000,000 | <0.001 | <0.001 |
| white-winged crossbill | *Loxia leucoptera* | 20 | 16 | 20,000,000 | <0.001 | <0.001 |
| Wilson's warbler | *Cardellina pusilla* | 1026 | 640 | 60,000,000 | 0.002 | 0.001 |
| winter wren | *Troglodytes hiemalis* | 1016 | 606 | 11,000,000 | 0.010 | 0.006 |
| wood thrush | *Hylocichla mustelina* | 878 | 592 | 11,000,000 | 0.008 | 0.005 |
| yellow warbler | *Setophaga petechia* | 1249 | 726 | 90,000,000 | 0.001 | 0.001 |
| yellow-bellied flycatcher | *Empidonax flaviventris* | 736 | 440 | 12,000,000 | 0.006 | 0.004 |
| yellow-breasted Chat | *Icteria virens* | 14 | 11 | 11,000,000 | <0.001 | <0.001 |
| yellow-headed blackbird | *Xanthocephalus xanthocephalus* | 1069 | 592 | 11,000,000 | 0.010 | 0.006 |
| yellow-rumped warbler | *Setophaga coronata* | 3502 | 2082 | 130,000,000 | 0.003 | 0.002 |
| yellow-throated vireo | *Vireo flavifrons* | 1139 | 639 | 3,500,000 | 0.035 | 0.019 |
| **Total** | **156 species** | 229,765 | 133,993 |  |  |  |
| ^a^ Average estimated number of fatalities each year adjusted by the bias value that was the lowest and for operating capacity (see text and Appendix S9)  ^b^ Average estimated number of fatalities each year adjusted by the bias value that was the highest and for operating capacity (see text and Appendix S9)  ^c^ Population estimates obtained from the Partners in Flight Landbird Population Estimates Database [1]  ^d^ Percent of population affected annually, using the estimate adjusted by the bias value that was the lowest  ^e^ Percent of population affected annually, using the estimate adjusted by the bias value that was the highest  ^f^ Only a global estimate was available  ^g^ Recently divided into two species: sagebrush sparrow (*Artemisiospiza nevadensis*) and Bell’s sparrow (*A. belli*) [2]  ^h^ Population estimate is the summed values for cordilleran and Pacific-slope flycatcher | | | | | | |

References:

1. Partners in Flight Science Committee (2013) Population Estimates Database, version 2013. Available at <http://rmbo.org/pifpopestimates>. Accessed on 31 December 2013.

2. Chesser RT, Banks RC, Barker FK, Cicero C, Dunn JL, et al. (2013) Fifty-fourth supplement to the American Ornithologists' Union Check-list of North American birds. Auk 130(3): 558-572.
